# Large language models as a diagnostic support tool in neuropathology

**DOI:** 10.1002/2056-4538.70009

**Published:** 2024-11-06

**Authors:** Katherine J Hewitt, Isabella C Wiest, Zunamys I Carrero, Laura Bejan, Thomas O Millner, Sebastian Brandner, Jakob Nikolas Kather

**Affiliations:** ^1^ Else Kröner Fresenius Center for Digital Health, Faculty of Medicine and University Hospital Carl Gustav Carus TUD Dresden University of Technology Dresden Germany; ^2^ Department of Medicine II, Medical Faculty Mannheim, Heidelberg University, Mannheim, Germany. Heidelberg University Mannheim Germany; ^3^ School of Medicine University College London London UK; ^4^ Division of Neuropathology, Queen Square Institute of Neurology University College London London UK; ^5^ Blizard Institute, Barts and The London School of Medicine and Dentistry Queen Mary University of London London UK; ^6^ Department of Medicine I, Faculty of Medicine and University Hospital Carl Gustav Carus TUD Dresden University of Technology Dresden Germany; ^7^ Medical Oncology, National Center for Tumor Disease University Hospital Heidelberg Heidelberg Germany

**Keywords:** large language models, neuropathology, adult‐type diffuse gliomas, decision support tools

## Abstract

The WHO guidelines for classifying central nervous system (CNS) tumours are changing considerably with each release. The classification of CNS tumours is uniquely complex among most other solid tumours as it incorporates not just morphology, but also genetic and epigenetic features. Keeping current with these changes across medical fields can be challenging, even for clinical specialists. Large language models (LLMs) have demonstrated their ability to parse and process complex medical text, but their utility in neuro‐oncology has not been systematically tested. We hypothesised that LLMs can effectively diagnose neuro‐oncology cases from free‐text histopathology reports according to the latest WHO guidelines. To test this hypothesis, we evaluated the performance of ChatGPT‐4o, Claude‐3.5‐sonnet, and Llama3 across 30 challenging neuropathology cases, which each presented a complex mix of morphological and genetic information relevant to the diagnosis. Furthermore, we integrated these models with the latest WHO guidelines through Retrieval‐Augmented Generation (RAG) and again assessed their diagnostic accuracy. Our data show that LLMs equipped with RAG, but not without RAG, can accurately diagnose the neuropathological tumour subtype in 90% of the tested cases. This study lays the groundwork for a new generation of computational tools that can assist neuropathologists in their daily reporting practice.

## Introduction

Large language models (LLMs) have shown promising performance on several healthcare‐related tasks including medical education [1, 2], and administrative tasks such as writing hospital discharge summaries [3] and clinic letters [4] for cancer patients in a variety of scenarios. While LLMs have also been applied to patient‐facing tasks, such as medical chatbots [5], concerns remain regarding hallucinations (where a generative response contains false or misleading information), their inability to provide references for factual information, and the lack of transparency in decision‐making [6].

One potential application of LLMs is as decision support tools to aid practitioners in the interpretation of clinical guidelines [7]. However, to our knowledge this has not been trialled in a neuropathology setting. We performed a PubMed search and found one study that assessed the diagnostic accuracy of vision‐LLMs on neuropathology images of neurodegenerative diseases [8]. A further seven studies examining the utility of LLMs for diagnostic support tasks within the field of neurology were identified, however none were applied in a neuropathology setting. Our search criteria and a summary of our findings can be found in supplementary material, Table S1.

Given the challenges presented by neuropathology diagnostics due to recent changes in the diagnostic approach and the multitude of potential diagnoses [9], we hypothesised that LLMs could potentially benefit neuropathology practitioners.

This work aims to assess the ability of three leading LLMs, one open‐source (Llama3‐70b from Meta) and two proprietary (ChatGPT‐4o from OpenAI, Claude‐3.5‐sonnet from Anthropic), to provide accurate diagnoses in neuropathology. Since adult‐type diffuse gliomas represent a significant proportion of the diagnostic work in adult neuro‐oncology practice [10], we created 30 realistic free‐text neuropathology reports and asked the models to make a diagnosis based on the histopathological description.

Additionally, we evaluated the standard ‘zero‐shot’ responses against Retrieval‐Augmented Generation (RAG) responses, where the models were provided with the latest WHO guidelines. RAG is a framework which limits the LLM to utilising data provided by the user, such as diagnostic guidelines, rather than relying on data acquired during training or from the internet. Moreover, this approach has been shown to mitigate some limitations of standard LLMs, including hallucinations [11].

Both responses were compared to an expert‐generated ground truth and for strict concordance with the latest WHO guidelines. Thereby, we hypothesise that the RAG responses will outperform the zero‐shot responses.

## Materials and methods

### Cohort description

We generated 30 artificial neuropathology cases: 10 each for astrocytoma, oligodendroglioma, and glioblastoma, with varying grade, morphological, and molecular features. For accuracy, these cases were based on real data, chosen semi‐randomly from the pseudonimised University College London Hospitals (UCLH) dataset. Cases were selected to represent a full range of features that a neuropathologist might encounter when reporting these entities. Only the diagnostic details necessary for reaching a diagnosis were kept, such as morphological features (e.g. gemistocytes, presence or absence of necrosis and/or mitoses, microvascular proliferations) and the results of further testing (e.g. immunohistochemistry and/or molecular analysis). Tumour descriptions were otherwise rephrased while trying to preserve different writing styles, including any grammar or spelling errors (e.g. ‘mitoticly’ and ‘cells positive’ instead of ‘cells are positive’). This experiment was conducted in concordance with the Declaration of Helsinki. No patient‐identifiable data were accessed or used during this project.

### RAG approach

Relevant diagnostic criteria are based on the CNS WHO 2021 fifth edition [9]. The chapters on adult‐type diffuse gliomas, along with paragraphs from the foreword and introduction discussing changes to the diagnostic approach from the previous edition, were collated into a Microsoft Word document. Relevant tables were converted to plain text to make the information accessible to the LLMs.

### Large language models

We compared ChatGPT‐4o, Claude‐3.5‐sonnet, and Llama3‐70b‐groq. The Llama3‐70b model with the groq extension was selected for its RAG capabilities, and was used for both the zero‐shot and RAG experiments. All models were accessed via a web interface: ChatGPT through chatgpt.com, Claude through claude.ai, and Llama3 through poe.com. Further details of the prompts used for both the zero‐shot and the RAG experiments are provided in supplementary material, Table S2. The experiments were conducted between 8 May and 26 June 2024.

### Analysis

The full neuropathological diagnosis consists of a histopathological diagnosis, a molecular diagnosis, and a grade [9] (e.g. astrocytoma, *IDH*‐mutant, CNS WHO grade 3). Each response generated by the network was reviewed for these three components, and was deemed correct only if all three completely matched the WHO guidelines. The diagnostic criteria for adult‐type diffuse gliomas can be found in Table 1.

**Table 1:** Diagnostic criteria

	Essential criteria	Additional information
Astrocytoma, *IDH*‐mutant	Diffusely infiltrating glioma AND *IDH*‐mutation AND *ATRX* mutation OR exclusion of whole arm deletions of 1p and 19q	ATRX mutation can be demonstrated as loss of nuclear ATRX expression on immunohistochemistry or *ATRX* mutation on molecular testing.
Oligodendroglioma, *IDH*‐mutant 1p/19q‐codeleted	Diffusely infiltrating glioma AND *IDH*‐mutation AND Whole arm deletions of 1p and 19q	Recommended that 1p/19q assays be able to detect whole‐arm chromosomal losses, such as FISH or molecular genetic testing.
Glioblastoma, *IDH*‐wildtype	Diffuse astrocytic glioma AND *IDH*‐wildtype AND H3‐wildtype	Must additionally demonstrate one or more of: microvascular proliferation, necrosis, *TERT* promoter mutation, *EGFR* gene amplification, chromosome +7/−10 copy number alterations.

This table summarises the diagnostic criteria for the neuropathological diagnosis of adult‐type diffuse gliomas according to WHO CNS fifth edition [9]. Once a diffusely infiltrating glioma has been identified on histopathology, these criteria must be demonstrated either by immunohistochemistry or DNA sequencing. Alternatively, DNA methylation profiling can also be used. WHO CNS5 also recommends that grade is designated in Arabic numerals, rather than Roman numerals, as in previous editions.

## Results

### Zero‐shot LLMs are ineffective at neuropathology diagnosis

**Figure 1:**
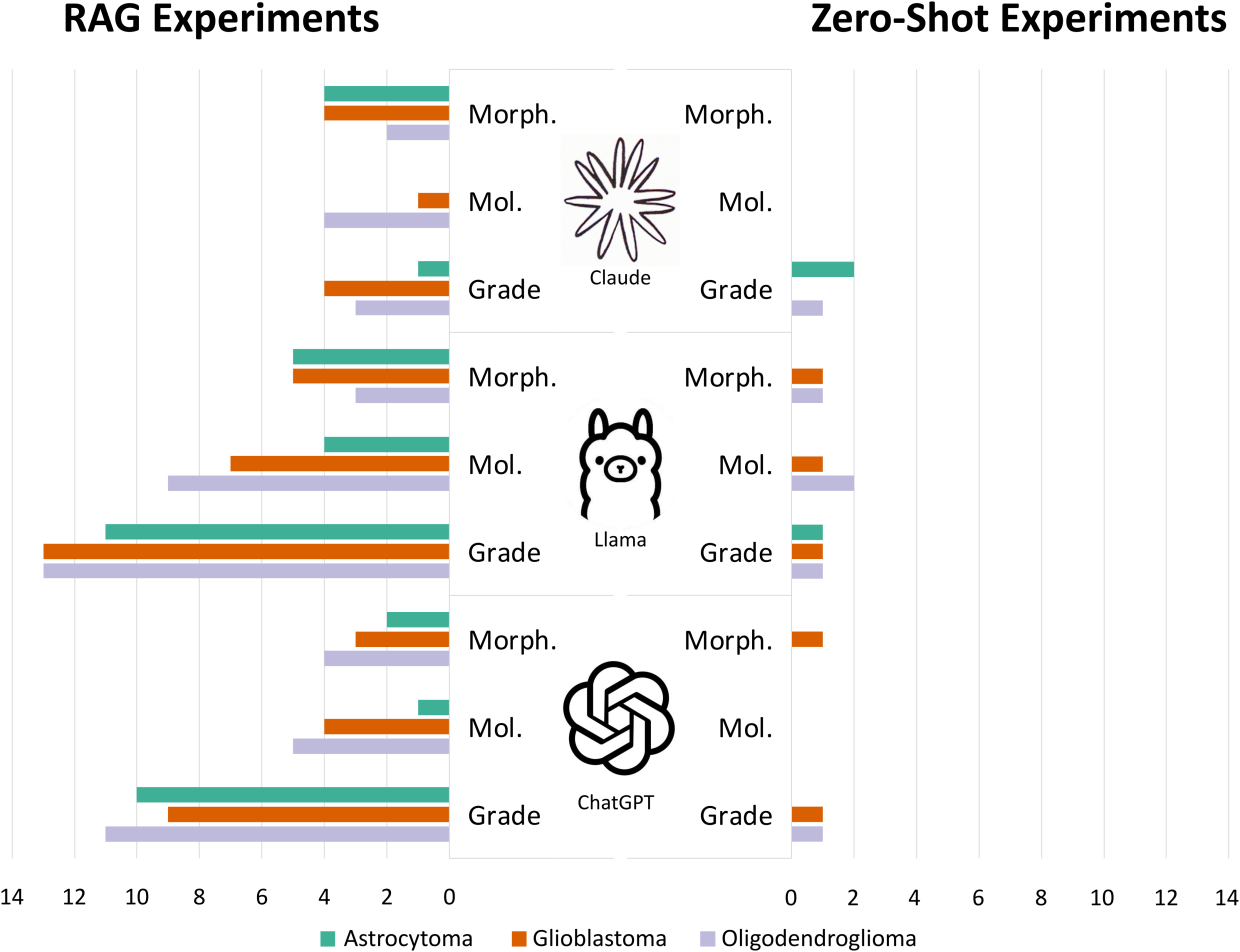
Mistakes by model and tumour type. This bar chart shows the total number of mistakes made by each model for each tumour type, where green represents astrocytoma, orange represents glioblastoma and grey oligodendroglioma. The bars are absent for some of the RAG experiments because no incorrect diagnoses were generated for these cases. The diagnostic accuracy of the models was judged according to three components: the morphological diagnosis, e.g. astrocytoma; the molecular diagnosis, e.g. *IDH*‐mutant; and the grade, e.g. grade 3. Errors related to grade were either that the incorrect grade was given by a model or the grade was given in Roman rather than Arabic numerals. Errors relating to morphology were either that the morphological diagnosis itself was incorrect, or an outdated term such as ‘anaplastic’ was used. Errors relating to molecular status were either that the molecular diagnosis provided was wrong, or a molecular status was not provided at all. In the instances where an outdated term or a grade in Roman numerals was provided, the response was deemed incorrect, regardless of whether the morphological diagnosis or the grade itself was correct. This level of accuracy was chosen because we wanted to hold the models to clinical standards. This figure shows that mistakes of all types were made much more frequently in the zero‐shot experiments than the RAG experiments.

We compared the ‘zero‐shot’ (standard) performance of all three models (zGPT, zLlama, and zClaude) in diagnosing adult‐type diffuse gliomas from neuropathological descriptions. Although the vast majority of LLM‐generated diagnoses were close to the ground truth, because we held the LLM to clinical standards many responses were classified as ‘incorrect’ by human experts (Table 2; Figure 1).

**Table 2:** Example results

Case number: ground truth	Experimental approach	Model and responses		
		ChatGPT	Llama	Claude
Case A2: Astrocytoma *IDH*‐mutant Grade 2	Zero‐shot	Astrocytoma *IDH*‐mutant Grade II	Astrocytoma *IDH*‐mutant Grade II	Astrocytoma *IDH*‐mutant Grade 2
	RAG	Astrocytoma *IDH*‐mutant Grade 2	Astrocytoma *IDH*‐mutant Grade 2	Astrocytoma *IDH*‐mutant Grade 2
Case G10: Glioblastoma *IDH*‐wildtype Grade 4	Zero‐shot	Glioblastoma *IDH*‐wildtype Grade 4	Anaplastic astrocytoma Grade III	Astrocytoma *IDH*‐wildtype Grade 4
	RAG	Glioblastoma *IDH*‐wildtype Grade 4	Glioblastoma *IDH*‐wildtype Grade 4	Glioblastoma *IDH*‐wildtype Grade 4
Case O9: Oligodendroglioma *IDH*‐mutant, 1p/19q co‐deleted Grade 3	Zero‐shot	Anaplastic oligodendrogl. *IDH*‐mutant, 1p/19q co‐del. Grade III	Glioblastoma *IDH*‐mutant Grade IV	Anaplastic oligodendrogl. *IDH*‐mutant, 1p/19q co‐del. Grade II
	RAG	Oligodendrogl. *IDH*‐mutant, 1p/19q co‐del. Grade 3	Astrocytoma *IDH*‐mutant Grade 3	Oligodendrogl. *IDH*‐mutant, 1p/19q co‐del. Grade 3

This table provides examples of the diagnoses given by each model, for each experimental approach and tumour type. One case for each tumour type was chosen and the diagnoses given by each model for both experimental approaches are provided, to illustrate how the models performed. Responses that were classified as incorrect are highlighted in red. In most instances, the diagnosis provided by the model was close to the ground truth, but were deemed incorrect because they failed to meet the current WHO guidance [9]. For example, the ChatGPT response in the zero‐shot experiment for case A2 was deemed incorrect because the grade was given in Roman, rather than in Arabic, numerals. This change to how grade should be noted was implemented by the most recent edition of the WHO CNS 5 guidelines. Many of the diagnostics errors made by the models in the zero‐shot experiments related to changes that were introduced in the WHO CNS 5 guidelines, such as astrocytoma and glioblastoma being exclusively *IDH*‐mutant and *IDH*‐wildtype respectively, and the term anaplastic no longer being used. Full responses are provided in supplementary material, Full responses by case.

In the astrocytoma cases, zClaude provided the correct diagnosis in 6/10 cases and zGPT in 1/10, whereas zLlama was unable to provide any correct diagnoses. For the glioblastoma cases, zClaude provided 5/10 correct diagnoses, zGPT provided 3/10, and zLlama provided no correct diagnoses. As for the oligodendroglioma cases, only zClaude provided correct diagnoses, doing so in 5/10 cases. Neither zGPT nor zLlama provided any correct responses.

**Figure 2:**
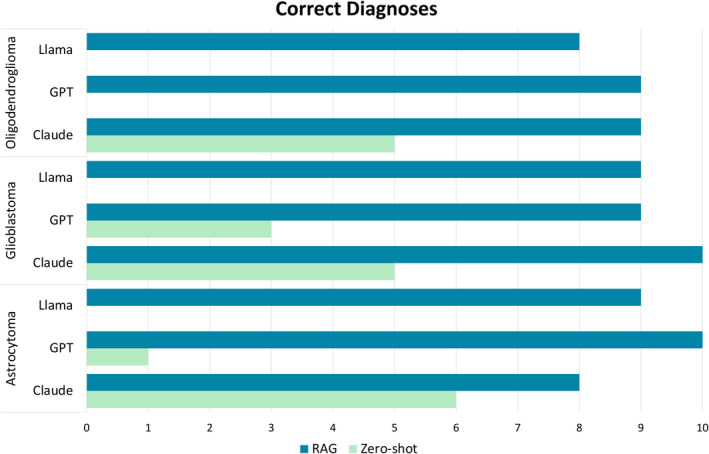
Correct diagnoses by tumour type. This bar chart presents the total number of correct diagnoses made by each model for each tumour type, where dark blue represents zero‐shot correct response and light green RAG correct responses. The bar is absent for some of the zero‐shot experiments because no correct diagnoses were generated for these cases. This figure demonstrates that correct diagnoses were made much more frequently by all models in the RAG experiments than the zero‐shot experiments.

Across all zero‐shot experiments, the correct diagnosis was given in just 20/90 cases (22.2%); 13.3% (*n* = 4) by zGPT and 53.3% (*n* = 16) by zClaude. zLlama provided no correct responses. The most common reason for responses being deemed incorrect was that the grade was given in Roman, rather than Arabic numerals. This was the case in 18/30 astrocytoma cases (60%), 18/30 glioblastoma cases (60%), and 21/30 oligodendroglioma cases (70%). Figures 1 and 2 provide an overview of the mistakes and correct diagnoses made by each model.

### RAG can provide an accurate neuropathology diagnosis

Next, we compared performance of all three models when utilising a RAG approach (rGPT, rLlama, and rClaude).

In the astrocytoma cases, rClaude provided the correct diagnosis in 8/10 cases, rLlama in 9/10 cases, and rGPT in 10/10 cases. For the glioblastoma cases, rClaude provided the correct diagnosis in 10/10 cases, while rGPT and rLlama both provided the correct diagnosis in 9/10 cases. As for the oligodendroglioma cases, both rGPT and rClaude correctly diagnosed 9/10 cases, whereas rLlama correctly diagnosed 8/10 cases.

Across all RAG experiments, the correct diagnosis was provided in 81/90 cases (90%); 93.3% (*n* = 28) by rGPT, 86.7% (*n* = 26) by rLlama, and 90% (*n* = 27) by rClaude. A results summary can be found in Figures 1 and 2.

Summaries of the results given by all three models across both the zero‐shot and RAG experiments for the astrocytoma, glioblastoma and oligodendrogioma case experiments are provided in supplementary material, Tables S3, S4 and S5 respectively.

## Discussion

Our study has evaluated the performance of LLMs on neuropathology cases, providing evidence against the use of zero‐shot LLMs within this field. Despite all three models being capable of providing neuropathology diagnoses based on histological descriptions of brain tumours, the diagnoses frequently used outdated terminology and often failed to meet current diagnostic standards. Conversely, the RAG approach yielded higher‐quality results, providing an accurate diagnosis in 90% of cases. ChatGPT with RAG was the most accurate among the models, with only two misdiagnoses. One misdiagnosis consisted of an incorrect grade in an oligodendroglioma case, which is arguably subjective and prone to inter‐observer variability, even among experts. However the other misdiagnosis was more consequential, as it was a glioblastoma case misdiagnosed as a low‐grade astrocytoma. This has significant impact for both management [12] and outcome [10], but as this case was an ‘early’ glioblastoma, it also presents a diagnostic challenge.

Our findings support RAG's potential to improve the diagnostic capabilities of LLMs. By constraining the natural language processing power of LLMs within clinical guidelines, RAG can mitigate some of the primary concerns associated with LLMs, such as lack of transparency and fabricated results. Through additional prompt engineering, LLMs with RAG could benefit clinicians working in neuropathology.

Limitations of our study include the scope. Although adult‐type diffuse gliomas are a fundamental part of neuropathology, numerous other entities are routinely encountered by neuropathologists on a daily basis. Further work to evaluate LLMs on a wider variety of entities and differentials, obtained from a variety of different data sources, is necessary. This should include rare cases and cases where the diagnosis is uncertain, such as entities which are a diagnosis of exclusion, to assess how the LLMs deal with ambiguity.

Additionally, before any AI model can be used clinically, the issue of safeguarding confidential patient data needs to be addressed. Many approaches for this exist, including computational privacy‐preserving techniques [13] and locally run LLM anonymisation pipelines [14]. Regardless of the approach, the underlying principle remains the same: sensitive data should not be shared with cloud‐based services (including LLMs) unless the provider can ensure that the data is treated according to the relevant legislations, i.e. Health Insurance Portability and Accountability Act in the USA and General Data Protection Regulation in the European Union.

## Author contributions statement

KJH is involved in the conception of the study; data acquisition and analysis; writing of first draft; review and critique; and final approval. ICW is involved in the conception of the study; review and critique; and final approval. ZIC is responsible for review and critique; and final approval. LB is involved in data acquisition; review and critique; and final approval. TOM is involved in the data acquisition; review and critique; and final approval. SB is involved in the data acquisition; review and critique; and final approval. JNK is involved in the conception of the study; review and critique; and final approval.

## Supporting information


**Table S1.** Results of literature review
**Table S2.** Prompts
**Table S3.** Summary of astrocytoma case results
**Table S4.** Summary of glioblastoma case results
**Table S5.** Summary of oligodendroglioma case results
Full responses by case

## Data Availability

The UCLH dataset was acquired through Brain UK (REF: 22/011). Inquiries regarding access to this dataset should be directed to SB. Llama3‐70b‐groq is freely accessible via poe.com, whereas ChatGPT‐4o requires a paid subscription. Anthropic offers limited free access to Claude, but full access requires a paid subscription.
