# Supplementary material for: Large language models as a diagnostic support tool in neuropathology
Source: J Pathol Clin Res. 2024 Nov 6;10(6):e70009. doi: 10.1002/2056-4538.70009 (PMC11540532; doi:10.1002/2056-4538.70009)
Supplement: Supplementary file 1 — Table S1. Results of literature review Table S2. Prompts Table S3. Summary of astrocytoma case results Table S4. Summary of glioblastoma case results Table S5. Summary of oligodendroglioma case results Full responses by case [file CJP2-10-e70009-s001.pdf]

Large Language Models as a Diagnostic Support Tool in Neuropathology

KJ Hewitt *et al J Pathol Clin Res* https://doi.org/10.1002/2056-4538.74009

Supplementary Material

Index

Summary Tables	Page No.
Table S1: Results of Literature Review	2
Table S2: Prompts	3
Table S3: Summary of Astrocytoma Case Results	4
Table S4: Summary of Glioblastoma Case Results	6
Table S5: Summary of Oligodendroglioma Case Results	8

Full Responses by Case

Astrocytoma Case	Page No.	Glioblastoma Case	Page No.	Oligodendroglioma Case	Page No.
A1	10	G1	57	O1	106
A2	15	G2	61	O2	111
A3	20	G3	67	O3	116
A4	24	G4	72	O4	121
A5	28	G5	77	O5	126
A6	33	G6	81	O6	130
A7	37	G7	86	O7	135
A8	42	G8	91	O8	140
A9	47	G9	96	O9	145
A10	52	G10	101	O10	150

Reference	Specialty	Task	Modality
Wada et al[15]	Neuroradiology	Diagnostic accuracy	Images + Text
Ono, Dickson & Koga[9]	Neurodegenerative disease neuropathology	Diagnostic accuracy and image interpretation	Images + Text
Kumar et al[16]	Neurosurgery	Differential diagnosis formulation	Text
Zaki et al[17]	Radiology (incl. neuro-radiology)	Predicting next imaging step for reaching a diagnosis	Text
Madadi et al[18]	Neuro-ophthalmology	Diagnostic accuracy	Text
Chen et al[19]	Neurology	Clinical evaluation	Text
Koga, Martin & Dickson[20]	Neurodegenerative disease	Diagnostic accuracy	Text
Nazario-Johnson, Zaki & Tung[21]	Neuro-radiology	Predicting next imaging step for reaching a diagnosis	Text
**Total:**			**8**

TABLE S1. RESULTS OF LITERATURE REVIEW

This table provides an overview of the results from the literature review we performed as part of this work. Studies were included where neurology patient cases, either real or synthetic, were given to LLMs to assess their utility in the diagnostic process and compared to expert opinion. We only identified one study that used LLMs for diagnostic support in a neuropathology setting, however this work primarily focussed on image interpretation for neurodegenerative diseases. Seven other studies were identified that used LLMs for diagnostic support in other areas of clinical neurology, and a brief outline of their application is provided in the table. The search strategy we used to identify relevant studies is given below.

(((neuropathology[Title/Abstract]) OR ((brain[Title/Abstract]) AND (pathology[Title/Abstract]))) OR (neuro*[Title/Abstract])) AND ((LLM[Title/Abstract]) OR (large language model[Title/Abstract]))
Filters: from 2019 - 2024

Search performed: 28.08.24

**Zero-shot prompt:**	**Attachment**
I will give you a histopathological description of a tumour and you will tell me the type of tumour you think it is, including grade (where relevant).<br><br>(free text description of the histopathological features and IHC/molecular pathology results)<br><br>What do you think the diagnosis would be?	(none)
**RAG prompt:**	
I will give you a histopathological description of a tumour and you will tell me the type of tumour you think it is, including grade (where relevant).<br>I have attached a microsoft Word document that contains the most recent WHO diagnostic guidelines for reaching a histopathological diagnosis in neuropathological tumours. All the information that is required to make a diagnosis can be found within the attached document. Make sure that you use the attached document to reach a diagnosis. Do not use any prior knowledge or the internet to make this diagnosis, use ONLY the document attached to this prompt. Make sure to provide references to the attached document with any conclusions that you make.<br>Are these instructions clear or do you require further information?<br><br>(free text description of the histopathological features and IHC/molecular pathology results submitted)<br><br>What do you think the diagnosis would be?	WHO CNS5 Microsoft Word document

Table S2: PROMPTS

This table provides the prompts that were used for both the zero-shot and RAG experiments, including whether an attachment was provided.
All experiments were completed between 8th May and 26th June 2024. Each case was submitted as a new chat.

Case	Ground Truth	Zero Shot Experiments			RAG Experiments		
		ChatGPT	Meta Llama 3	Claude 3.5 Sonnet	ChatGPT	Meta Llama 3	Claude 3.5 Sonnet
**A1**	Astrocytoma IDH-mutant Grade 2	Astrocytoma IDH-mutant Grade II	Astrocytoma Grade II	Astrocytoma IDH-mutant Grade 2	Astrocytoma IDH-mutant Grade 2	Astrocytoma IDH-mutant Grade 2	Astrocytoma IDH-mutant Grade 2
**A2**	Astrocytoma IDH-mutant Grade 2	Astrocytoma IDH-mutant Grade II	Astrocytoma IDH-mutant Grade II	Astrocytoma IDH-mutant Grade 2	Astrocytoma IDH-mutant Grade 2	Astrocytoma IDH-mutant Grade 2	Astrocytoma IDH-mutant Grade 2
**A3**	Astrocytoma IDH-mutant Grade 2	Astrocytoma IDH-mutant Grade II	Astrocytoma IDH-mutant Grade II	Astrocytoma IDH-mutant Grade 2	Astrocytoma IDH-mutant Grade 2	Astrocytoma IDH-mutant Grade 2	Astrocytoma IDH-mutant Grade 3
**A4**	Astrocytoma IDH-mutant Grade 2	Astrocytoma IDH-mutant Grade 2	Astrocytoma IDH-mutant Grade II	Astrocytoma IDH-mutant Grade 2	Astrocytoma IDH-mutant Grade 2	Astrocytoma IDH-mutant Grade 2	Astrocytoma IDH-mutant Grade 2
**A5**	Astrocytoma IDH-mutant Grade 2	Astrocytoma IDH-mutant Grade II	Astrocytoma Grade II	Astrocytoma IDH-mutant Grade 2	Astrocytoma IDH-mutant Grade 2	Astrocytoma IDH-mutant Grade 2	Astrocytoma IDH-mutant Grade 2
**A6**	Astrocytoma IDH-mutant Grade 3	Astrocytoma IDH-mutant Grade II	Anaplastic oligoastrocytoma Grade III	Anaplastic astrocytoma IDH-mutant Grade 3	Astrocytoma IDH-mutant Grade 3	Astrocytoma IDH-mutant Grade 3	Astrocytoma IDH-mutant Grade 3

**A7**	Astrocytoma IDH-mutant Grade 3	Anaplastic astrocytoma IDH-mutant Grade III	Anaplastic astrocytoma IDH-mutant Grade III	Astrocytoma IDH-mutant Grade 3	Astrocytoma IDH-mutant Grade 3	Astrocytoma IDH-mutant Grade 3	Astrocytoma IDH-mutant Grade 3
**A8**	Astrocytoma IDH-mutant Grade 4	Astrocytoma IDH-mutant Grade IV	Glioblastoma IDH-mutant Grade IV	Anaplastic astrocytoma IDH-mutant WHO Grade 3	Astrocytoma IDH-mutant Grade 4	Astrocytoma IDH-mutant Grade 4	Astrocytoma IDH-mutant Grade 4
**A9**	Astrocytoma IDH-mutant Grade 4	Glioblastoma IDH-wildtype Grade 4	Glioblastoma Grade IV	Glioblastoma IDH-mutant Grade 4	Astrocytoma IDH-mutant Grade 4	Astrocytoma IDH-mutant Grade 4	Astrocytoma IDH-mutant Grade 4
**A10**	Astrocytoma IDH-mutant Grade 4	Astrocytoma IDH-mutant Grade III	Astrocytoma IDH-mutant Grade II	Glioblastoma IDH-mutant Grade 4	Astrocytoma IDH-mutant Grade 4	Astrocytoma IDH-mutant Grade 2	Astrocytoma IDH-mutant Grade 3

TABLE S3. SUMMARY OF ASTROCYTOMA CASE RESULTS

This table provides a summary of the results given by all three models across both the zero-shot and RAG experiments for the Astrocytoma case experiments. Responses that were deemed incorrect are highlighted in red. Only the “bottom-line diagnosis” provided by the model is given in this table. The full text responses generated by the models can be found in the following Astrocytoma Case section.

Case	Ground Truth	Zero Shot Experiments			RAG Experiments		
		ChatGPT	Meta Llama 3	Claude 3.5 Sonnet	ChatGPT	Meta Llama 3	Claude 3.5 Sonnet
**G1**	Glioblastoma IDH-wildtype Grade 4	Glioblastoma (multiforme) Grade IV	Glioblastoma Grade IV	Glioblastoma IDH-wildtype Grade 4	Glioblastoma IDH-wildtype Grade 4	Glioblastoma IDH-wildtype Grade 4	Glioblastoma IDH-wildtype Grade 4
**G2**	Glioblastoma IDH-wildtype Grade 4	Glioblastoma Grade IV	Glioblastoma Grade IV	Glioblastoma IDH-wildtype Grade IV	Glioblastoma IDH-wildtype Grade 4	Glioblastoma IDH-wildtype Grade 4	Glioblastoma IDH-wildtype Grade 4
**G3**	Glioblastoma IDH-wildtype Grade 4	Glioblastoma IDH-wildtype Grade 4	Glioblastoma IDH-wildtype Grade IV	Astrocytoma IDH-wildtype Grade 2	Glioblastoma IDH-wildtype Grade 4	Glioblastoma IDH-wildtype Grade 4	Glioblastoma IDH-wildtype Grade 4
**G4**	Glioblastoma IDH-wildtype Grade 4	Astrocytoma Grade II	Anaplastic astrocytoma Grade III	Astrocytoma Grade 3	Glioblastoma IDH-wildtype Grade 4	Glioblastoma IDH-wildtype Grade 4	Glioblastoma IDH-wildtype Grade 4
**G5**	Glioblastoma IDH-wildtype Grade 4	Glioblastoma Grade IV	Glioblastoma Grade IV	Glioblastoma IDH-wildtype Grade 4	Glioblastoma IDH-wildtype Grade 4	Glioblastoma IDH-wildtype Grade 4	Glioblastoma IDH-wildtype Grade 4
**G6**	Glioblastoma IDH-wildtype Grade 4	Glioblastoma IDH-wildtype Grade IV	Glioblastoma IDH-wildtype Grade IV	Glioblastoma IDH-wildtype Grade 4	Glioblastoma, IDH-wildtype Grade 4	Glioblastoma IDH-wildtype Grade 4	Glioblastoma IDH-wildtype Grade 4
**G7**	Glioblastoma IDH-wildtype Grade 4	Glioblastoma IDH-wildtype Grade IV	Glioblastoma IDH-wildtype Grade IV	Glioblastoma IDH-wildtype Grade 4	Glioblastoma IDH-wildtype Grade 4	Glioblastoma IDH-wildtype Grade 4	Glioblastoma IDH-wildtype Grade 4

**G8**	Glioblastoma IDH-wildtype Grade 4	Glioblastoma IDH-wildtype Grade 4	Glioblastoma Grade IV	Glioblastoma IDH-wildtype Grade 4	Glioblastoma IDH-wildtype Grade 4	Glioblastoma IDH-wildtype Grade 4	Glioblastoma IDH-wildtype Grade 4
**G9**	Glioblastoma IDH-wildtype Grade 4	Astrocytoma IDH-wildtype Grade II	Astrocytoma Grade II	Astrocytoma IDH-wildtype Grade 2	Astrocytoma IDH-wildtype Grade 2	Astrocytoma IDH-mutant Grade 2	Glioblastoma IDH-wildtype Grade 4
**G10**	Glioblastoma IDH-wildtype Grade 4	Glioblastoma IDH-wildtype Grade 4	Anaplastic astrocytoma Grade III	Astrocytoma IDH-wildtype Grade 4	Glioblastoma IDH-wildtype Grade 4	Glioblastoma IDH-wildtype Grade 4	Glioblastoma IDH-wildtype Grade 4

TABLE S4. SUMMARY OF GLIOBLASTOMA CASE RESULTS

This table provides a summary of the results given by all three models across both the zero-shot and RAG experiments for the Glioblastoma case experiments. Responses that were deemed incorrect are highlighted in red. Only the “bottom-line diagnosis” provided by the model is given in this table. The full text responses generated by the models can be found in the following Glioblastoma Case section.

Case	Ground Truth	Zero Shot Experiments			RAG Experiments		
		ChatGPT	Meta Llama 3	Claude 3.5 Sonnet	ChatGPT	Meta Llama 3	Claude 3.5 Sonnet
**O1**	Oligodendroglioma IDH-mutant, 1p/19q-codeleted Grade 2	Oligodendrog. Grade II	Oligodendrog. WHO Grade II	Oligodendrog. IDH-mut., 1p/19q-codel. Grade 2	Oligodendrog. IDH-mut., 1p/19q-codel. Grade 2	Oligodendro. IDH-mut., 1p/19q-codel. Grade 2	Oligodendro. IDH-mut., 1p/19q-codel. Grade 2
**O2**	Oligodendroglioma IDH-mutant, 1p/19q-codeleted Grade 2	Oligodendrog. Grade II or III	Oligodendrog. Grade II	Oligodendrog. Grade 2	Oligodendrog. IDH-mut., 1p/19q-codel. Grade 2 or 3	Oligodendrog. IDH-mut., 1p/19q-codel. Grade 2	Oligodendro. IDH-mut., 1p/19q-codel. Grade 2
**O3**	Oligodendroglioma IDH-mutant, 1p/19q-codeleted Grade 2	Oligodendrog. IDH-mut., 1p/19q co-del. Grade II	Oligodendrog. Grade II	Oligodendrog. IDH-mut., 1p/19q-codel. Grade 2	Oligodendrog. IDH-mut., 1p/19q-codel. Grade 2	Oligodendrog. IDH-mut., 1p/19q-codel. Grade 2	Oligodendro. IDH-mut., 1p/19q-codel. Grade 2
**O4**	Oligodendroglioma IDH-mutant, 1p/19q-codeleted Grade 2	Oligodendrog. IDH-mut., 1p/19q co-del., Grade II	Oligodendrog. Grade II	Oligodendrog. IDH-mut., 1p/19q-codel. Grade 2	Oligodendrog. IDH-mut., 1p/19q-codel. Grade 2	Oligodendrog. IDH-mut., 1p/19q-codel. Grade 2	Oligodendro. IDH-mut., 1p/19q-codel. Grade 2
**O5**	Oligodendroglioma IDH-mutant, 1p/19q-codeleted Grade 2	Oligodendrog. IDH-mut., 1p/19q co-del. Grade II	Oligodendrog. Grade II	Oligodendrog. IDH-mut., 1p/19q-codel. Grade 2	Oligodendrog. IDH-mut., 1p/19q-codel. Grade 2	Oligodendrog. IDH-mut., 1p/19q-codel. Grade 2	Oligodendro. IDH-mut., 1p/19q-codel. Grade 2
**O6**	Oligodendroglioma IDH-mutant, 1p/19q-codeleted Grade 3	Anaplastic oligodendrog. Grade III	Anaplastic oligodendrog. Grade III	Anaplastic oligodendrog. Grade 3	Oligodendrog. IDH-mut., 1p/19q-codel. Grade 3	Oligodendrog. IDH-mut., 1p/19q-codel. Grade 3	Oligodendro. IDH-mut., 1p/19q-codel. Grade 3

**O7**	Oligodendroglioma IDH-mutant, 1p/19q-codeleted Grade 3	Anaplastic Oligodendrog. Grade III	Anaplastic oligodendrog. Grade III	Oligodendrog. Grade 3	Oligodendrog. IDH-mut., 1p/19q-codel. Grade 3	Oligodendrog. IDH-mut., 1p/19q-codel. Grade 3	Oligodendro. IDH-mut., 1p/19q-codel. Grade 3
**O8**	Oligodendroglioma IDH-mutant, 1p/19q-codeleted Grade 3	Oligodendrog. WHO Grade II	Oligodendrog. Grade II	Oligodendrog. IDH-mut., 1p/19q-codel. Grade 2	Oligodendrog. IDH-mut., 1p/19q-codel. Grade 2	Oligodendrog. IDH-mutant Grade 2	Oligodendro. IDH-mut., 1p/19q-codel. Grade 2
**O9**	Oligodendroglioma IDH-mutant, 1p/19q-codeleted Grade 3	Anaplastic oligodendrog. IDH-mut., 1p/19q co-del. Grade III	Glioblastoma Grade IV IDH-mutant	Anaplastic oligodendrog. IDH-mut., 1p/19q-codel. Grade III	Oligodendrog. IDH-mut., 1p/19q-codel. Grade 3	Astrocytoma IDH-mutant Grade 3	Oligodendro. IDH-mut., 1p/19q-codel. Grade 3
**O10**	Oligodendroglioma IDH-mutant, 1p/19q-codeleted Grade 3	Anaplastic oligodendrog. IDH-mut., 1p/19q-codel. Grade III	Oligodendrog. IDH-mutant, 1p/19q-codel. Grade II	Oligodendrog. Grade 2	Oligodendrog. IDH-mut., 1p/19q-codel. Grade 3	Oligodendrog. IDH-mut., 1p/19q-codel. Grade 2	Oligodendro. IDH-mut., 1p/19q-codel. Grade 3

TABLE S5. SUMMARY OF OLIGODENDROGLIOMA CASE RESULTS

This table provides a summary of the results given by all three models across both the zero-shot and RAG experiments for the Oligodendroglioma case experiments. Responses that were deemed incorrect are highlighted in red. Only the “bottom-line diagnosis” provided by the model is given in this table. The full text responses generated by the models can be found in the following Oligodendroglioma Case section.

Astrocytoma Cases

A1: NH14 1353 - A2 IDHmut

Numerous pieces from a monomorphous, and moderately cellular glial tumour. The constituent tumour cells exhibit round nuclei and homogenous chromatin. No mitotic figures are identified, although approximately half of this specimen is haemorrhagic.
Mutant IDH immunohistochemistry reveals weak positive labelling and repeat testing has been requested.
ATRX staining demonstrates a loss in the malignant cell nuclei, with only endothelial cells retaining positivity.
Molecular testing confirmed that the tumour is IDH mutant without 1p19q co-deletion.

What do you think the diagnosis would be?

GT: Astrocytoma, IDH-mutant, CNS WHO grade 2

Llama Response:

Based on the histopathological description and the immunohistochemical results, I think the diagnosis is likely to be a **Grade II Diffuse Astrocytoma**.

Here's my reasoning:

- The tumor cells are described as monomorphous and moderately cellular, which is consistent with a low-grade glioma.
- The lack of mitotic figures suggests a low proliferative activity, which is also consistent with a low-grade glioma.
- The weak positive labeling with mutant IDH immunohistochemistry and the loss of ATRX staining in the tumor cell nuclei are both characteristic features of diffuse astrocytomas.
- The molecular testing confirming an IDH mutation without 1p19q co-deletion is also consistent with a diffuse astrocytoma.

Based on the WHO classification, a Grade II diffuse astrocytoma is a low-grade glioma that can exhibit a range of morphological features, including a monomorphous and moderately cellular appearance. The lack of mitotic figures and the presence of an IDH mutation are consistent with a Grade II rather than a higher-grade diffuse astrocytoma.

Llama RAG response:

Based on the histopathological description and the information provided in the attached document, I believe the diagnosis is **IDH-mutant Astrocytoma, Grade 2**.

Here's my reasoning:

- The tumour is described as "monomorphous" and "moderately cellular", which suggests a glial tumour (Section "CNS Tumour Nomenclature" in the document).
- The presence of round nuclei and homogenous chromatin is consistent with an astrocytoma (Section "CNS Tumour Nomenclature" in the document).
- The absence of mitotic figures suggests a low-grade tumour (Section "CNS Tumour Grading" in the document).
- The positive IDH immunohistochemistry and the loss of ATRX staining in the malignant cell nuclei are consistent with an IDH-mutant astrocytoma (Section "Gene and Protein Nomenclature" and "Combined histological and molecular grading" in the document).
- The molecular testing confirming the IDH mutation without 1p19q co-deletion is also consistent with an IDH-mutant astrocytoma (Section "Combined histological and molecular grading" in the document).
- Based on the above features, I would assign a grade of 2 to this tumour, as it is a low-grade tumour with no mitotic figures, but with IDH mutation and loss of ATRX staining (Section "Grading within Types" in the document).

Reference: WHO Classification of Tumours, 5th edition, Section "CNS Tumour Nomenclature", "CNS Tumour Grading", "Gene and Protein Nomenclature", and "Combined histological and molecular grading".

Claude Response:

Based on the histopathological description and molecular findings you've provided, the diagnosis is most likely:

Diffuse Astrocytoma, IDH-mutant, WHO grade 2

Here's the reasoning behind this diagnosis:

1. Histology: The description of a "monomorphous, and moderately cellular glial tumour" with cells exhibiting "round nuclei and homogenous chromatin" is consistent with a diffuse glioma. The absence of mitotic figures suggests a lower-grade tumor.
2. IDH status: The tumor is IDH-mutant, which is confirmed by both immunohistochemistry (although weak and pending repeat testing) and molecular testing.
3. ATRX status: Loss of ATRX staining in tumor cell nuclei is characteristic of astrocytomas and is not seen in oligodendrogliomas.
4. 1p19q status: The tumor lacks 1p19q co-deletion, which rules out oligodendroglioma.
5. Grade: The absence of mitotic figures, necrosis, or microvascular proliferation suggests a lower-grade tumor. Given the IDH-mutant status and absence of high-grade features, this is most consistent with a WHO grade 2 tumor.

The combination of IDH mutation, ATRX loss, and absence of 1p19q co-deletion is the typical molecular profile of a diffuse astrocytoma. The histological features described also align with this diagnosis.

It's worth noting that if additional high-grade features are found in other areas of the tumor not represented in this sample, or if the Ki-67 proliferation index (not mentioned here) is elevated, the grade

could potentially be higher. However, based on the information provided, a grade 2 diffuse astrocytoma is the most likely diagnosis.

Claude RAG response:

Based on the histopathological description and molecular findings provided, and using only the information from the attached WHO diagnostic guidelines document, the diagnosis appears to be:

Astrocytoma, IDH-mutant, CNS WHO grade 2

Reasoning:

1. The tumor is described as a "monomorphous, and moderately cellular glial tumour" with cells exhibiting "round nuclei and homogenous chromatin." This description is consistent with the histopathology of IDH-mutant astrocytoma as described in the document: "CNS WHO grade 2 tumours are composed of well-differentiated fibrillary glial cells that diffusely infiltrate the CNS parenchyma individually, usually without cellular cohesion, generating a loosely structured, often microcystic matrix. Cellularity is mildly to moderately increased compared with that of normal brain, and mild nuclear atypia is characteristic."
2. The absence of mitotic figures is noted, which aligns with the description for CNS WHO grade 2 astrocytoma: "Mitotic activity is absent or uncommon in CNS WHO grade 2 tumours" (from the "Histopathology" section under "Astrocytoma, IDH-mutant").
3. The molecular testing confirmed IDH mutation without 1p19q co-deletion. This is a defining feature of IDH-mutant astrocytoma, as stated in the document: "Astrocytoma, IDH-mutant, is a diffusely infiltrating IDH1- or IDH2-mutant glioma with frequent ATRX and/or TP53 mutation and absence of 1p/19q codeletion" (from the "Definition" section under "Astrocytoma, IDH-mutant").
4. The loss of ATRX staining in malignant cell nuclei is consistent with IDH-mutant astrocytoma: "Inactivating ATRX alterations commonly co-occur with TP53 mutations in IDH-mutant astrocytomas. These often result in a truncated protein and abrogated protein expression, leading to loss of nuclear ATRX immunoreactivity" (from the "Diagnostic molecular pathology" section under "Astrocytoma, IDH-mutant").
5. The grade 2 designation is supported by the absence of features indicating higher grades, such as significant mitotic activity, microvascular proliferation, or necrosis, which would be required for grades 3 or 4 (as described in the "Grading" section under "Astrocytoma, IDH-mutant").

It's worth noting that while the IDH immunohistochemistry showed weak positive labelling and repeat testing was requested, the molecular testing confirmed the IDH mutation, which is sufficient for the diagnosis.

This diagnosis meets the essential diagnostic criteria outlined in the document for Astrocytoma, IDH-mutant: "Astrocytoma, IDH-mutant must display features of a diffusely infiltrating glioma. In addition, this tumour must have IDH1 codon 132 or IDH2 codon 172 missense mutation and either loss of nuclear ATRX expression, ATRX mutation or exclusion of combined whole-arm deletions of 1p and 19q" (from the "Essential and desirable diagnostic criteria" section under "Astrocytoma, IDH-mutant").

ChatGPT Response:

Based on the histopathological description and molecular findings provided, the diagnosis appears to be a ****diffuse astrocytoma, IDH-mutant****. Here is the rationale for this diagnosis:

1. **Monomorphous and Moderately Cellular Glial Tumour**: This suggests a relatively uniform appearance of the tumour cells, characteristic of a lower-grade glial tumour.
2. **Round Nuclei and Homogenous Chromatin**: These are features often seen in lower-grade gliomas.
3. **No Mitotic Figures**: The absence of mitotic activity supports a lower-grade tumour.
4. **Haemorrhagic Specimen**: While the presence of haemorrhage can be seen in various tumours, it does not specifically indicate a high-grade lesion in this context.
5. **Mutant IDH Immunohistochemistry (Weak Positive)**: This suggests the presence of an IDH mutation, a common feature in lower-grade gliomas, particularly diffuse astrocytomas and oligodendrogliomas.
6. **ATRX Staining (Loss in Malignant Cells)**: Loss of ATRX expression is commonly associated with astrocytomas and is a distinguishing feature from oligodendrogliomas, which retain ATRX expression.
7. **Molecular Testing (IDH Mutant, Without 1p19q Co-deletion)**: The presence of an IDH mutation without the 1p19q co-deletion confirms the tumour is an astrocytoma rather than an oligodendroglioma.

Grade
Given the described histopathological features, including the lack of mitotic figures and the moderately cellular nature, this tumour would typically be classified as a **Grade II** tumour according to the World Health Organization (WHO) classification of central nervous system tumours.

Therefore, the most appropriate diagnosis is:
****Diffuse astrocytoma, IDH-mutant, WHO Grade II.****

ChatGPT RAG response:

Based on the histopathological description and molecular findings, the diagnosis is **Astrocytoma, IDH-mutant, CNS WHO grade 2**.

Here's the reasoning and reference to the WHO guidelines:

Histopathological Features:

- **Monomorphous and moderately cellular glial tumor**: This indicates a relatively uniform appearance and moderate cellularity, characteristic of lower-grade gliomas.
- **Round nuclei and homogeneous chromatin**: These features are consistent with a well-differentiated glioma.
- **No mitotic figures identified**: The absence of mitotic activity is typical of a CNS WHO grade 2 tumor.
- **Approximately half of this specimen is haemorrhagic**: Haemorrhage can be seen in various gliomas but does not specifically define the grade.

Immunohistochemistry:

- **Mutant IDH immunohistochemistry reveals weak positive labelling**: Indicates the presence of IDH mutation.
- **ATRX staining demonstrates a loss in malignant cell nuclei, with only endothelial cells retaining positivity**: Loss of ATRX expression is commonly seen in IDH-mutant astrocytomas and helps differentiate them from oligodendrogliomas, which usually retain ATRX expression.

Molecular Testing:
- **Confirmed IDH mutant without 1p19q co-deletion**: This molecular profile is indicative of an IDH-mutant astrocytoma, as oligodendrogliomas require both an IDH mutation and 1p/19q co-deletion for diagnosis.

According to the WHO guidelines:
- Astrocytoma IDH-mutant is characterized by a **diffusely infiltrating IDH1- or IDH2-mutant glioma** with frequent ATRX and/or TP53 mutations and absence of 1p/19q co-deletion.
- CNS WHO grade 2 tumors are **well-differentiated** with **no significant mitotic activity, microvascular proliferation, or necrosis**【3†source】.

Therefore, considering all the histopathological and molecular data provided, the diagnosis aligns with ****Astrocytoma, IDH-mutant, CNS WHO grade 2****.

A2: NH17 2381 - A2 IDHmut

This is an extensive surgical specimen comprising a diffuse and moderately cellular glial tumour. The tumour cells nuclei exhibit round or polyclonal forms and contain compact, uniform chromatin. The majority of tumour cells display scant eosinophilic cytoplasm with short processes that create a reticular pattern. Occasional maturing neurons are seen entrapped within the tumour. The vascular infrastructure within the lesion displays an extensive network without any notable endothelial thickening or microvascular proliferations. Only rare mitotic figures were observed in this substantial tumour specimen.

Immunohistochemistry:
GFAP expression is diffuse in tumour cell cytoplasm and processes
IDH1 (R132H) expression is positive in tumour cells, indicating IDH1 mutation
ATRX expression is absent in tumour cell nuclei, indicating mutation
Ki67 (proliferation index) is very low <1%

What do you think the diagnosis would be?

GT: astrocytoma, IDH-mutant, CNS WHO grade 2

Llama Response:

What a delightful histopathological description!

Based on the morphological features and immunohistochemical results, I would diagnose this tumour as a:

****Diffuse Astrocytoma, IDH-mutant, WHO Grade II****

Here's why:
- The diffuse and moderately cellular glial tumour with round or polygonal nuclei and scant eosinophilic cytoplasm suggests an astrocytic tumour.
- The presence of entrapped neurons and the reticular pattern created by the tumour cells' short processes further support an astrocytic tumour.
- The absence of endothelial thickening and microvascular proliferations indicates a low-grade tumour.
- The immunohistochemical results are consistent with an IDH-mutant astrocytoma: GFAP expression is expected in astrocytic tumours, and the IDH1 (R132H) mutation is a common genetic alteration in diffuse astrocytomas. The loss of ATRX expression is also consistent with an IDH-mutant astrocytoma.
- The very low Ki67 proliferation index (<1%) is also consistent with a low-grade tumour.

Given the morphology and immunohistochemical results, I would classify this tumour as a WHO Grade II diffuse astrocytoma, IDH-mutant.

Please let me know if I'm correct or not!

Llama RAG response:

Based on the histopathological description and immunohistochemical results, I think the diagnosis would be:

****Diffuse Astrocytoma, IDH-mutant, Grade 2****

Here's my reasoning:

- The tumour is described as a diffuse and moderately cellular glial tumour, which is consistent with a diffuse astrocytoma (Page 1, "Introduction to CNS Tumours").
- The tumour cells have round or polygonal nuclei with compact, uniform chromatin, which is consistent with a low-grade astrocytoma (Page 1, "Introduction to CNS Tumours").
- The presence of occasional maturing neurons entrapped within the tumour is also consistent with a diffuse astrocytoma (Page 1, "Introduction to CNS Tumours").
- The vascular infrastructure within the lesion displays an extensive network without any notable endothelial thickening or microvascular proliferations, which suggests a low-grade tumour (Page 1, "Introduction to CNS Tumours").
- The immunohistochemical results show diffuse GFAP expression in tumour cell cytoplasm and processes, which is consistent with an astrocytic tumour (Page 1, "Introduction to CNS Tumours").
- The IDH1 (R132H) expression is positive in tumour cells, indicating an IDH1 mutation, which is a characteristic feature of diffuse astrocytomas (Page 1, "Introduction to CNS Tumours").
- The ATRX expression is absent in tumour cell nuclei, indicating a mutation, which is also consistent with a diffuse astrocytoma (Page 1, "Introduction to CNS Tumours").
- The Ki67 (proliferation index) is very low (<1%), which suggests a low-grade tumour (Page 1, "Introduction to CNS Tumours").

According to the grading system described in the document (Page 5, "Grading within Types"), the lack of mitotic activity and the low Ki67 index suggest a Grade 2 tumour.

Therefore, based on the above features, I believe the diagnosis is a Diffuse Astrocytoma, IDH-mutant, Grade 2.

Claude Response:

Based on the histopathological description and immunohistochemistry results provided, the diagnosis is most likely:

Diffuse Astrocytoma, IDH-mutant, WHO Grade 2

Key features supporting this diagnosis:
1. Diffuse and moderately cellular glial tumor
2. Round to polyclonal nuclei with uniform chromatin
3. Reticular pattern of tumor cell processes
4. Absence of significant mitotic activity
5. Lack of microvascular proliferation or endothelial thickening
6. Diffuse GFAP expression, indicating astrocytic lineage

7. IDH1 (R132H) mutation, a hallmark of diffuse gliomas
8. Loss of ATRX expression, commonly associated with IDH-mutant astrocytomas
9. Very low Ki67 proliferation index (<1%)

The WHO grade 2 classification is supported by the low mitotic activity and absence of necrosis or microvascular proliferation. The IDH mutation and ATRX loss are typical of diffuse astrocytomas, distinguishing it from oligodendrogliomas (which typically retain ATRX expression but show 1p/19q codeletion).

The presence of entrapped neurons is not uncommon in diffuse gliomas and doesn't change the diagnosis in this context.

This diagnosis aligns with the 2021 WHO Classification of Tumors of the Central Nervous System. However, it's important to note that final diagnosis should always be made by a qualified pathologist with access to all clinical and radiological information.

Claude RAG response:

Based on the histopathological description and immunohistochemistry results provided, and using only the information from the attached WHO diagnostic guidelines document, I believe the diagnosis is:

Astrocytoma, IDH-mutant, CNS WHO grade 2

Here's my reasoning, referencing the attached document:

1. Diffuse growth pattern: The description states "a diffuse and moderately cellular glial tumour," which aligns with the definition of astrocytoma, IDH-mutant as "a diffusely infiltrating IDH1- or IDH2-mutant glioma" (from the Definition section of Astrocytoma, IDH-mutant).
2. IDH mutation: The immunohistochemistry shows "IDH1 (R132H) expression is positive in tumour cells, indicating IDH1 mutation." This is a key diagnostic criterion for Astrocytoma, IDH-mutant, as stated in the Essential and desirable diagnostic criteria section: "this tumour must have IDH1 codon 132 or IDH2 codon 172 missense mutation."
3. ATRX mutation: The immunohistochemistry shows "ATRX expression is absent in tumour cell nuclei, indicating mutation." This supports the diagnosis of Astrocytoma, IDH-mutant, as mentioned in the Diagnostic molecular pathology section: "Loss of nuclear ATRX expression in neoplastic cells strongly supports the diagnosis of IDH-mutant astrocytoma."
4. Low-grade features: The description mentions "Only rare mitotic figures were observed" and "Ki67 (proliferation index) is very low <1%." This aligns with the description of CNS WHO grade 2 tumors in the Grading section: "Mitotic activity is not detected or very low."
5. Absence of high-grade features: The description does not mention microvascular proliferation, necrosis, or high cellularity, which are features of higher-grade tumors. The vascular description specifically states "without any notable endothelial thickening or microvascular proliferations."
6. GFAP expression: The immunohistochemistry shows "GFAP expression is diffuse in tumour cell cytoplasm and processes," which is consistent with the Immunophenotype section stating "Individual tumour cells of IDH-mutant astrocytoma reliably express GFAP, although to varying degrees."

ChatGPT Response:

The histopathological description provided is consistent with a **diffuse astrocytoma**, IDH-mutant (WHO Grade II).

Key features supporting this diagnosis include:
1. **Diffuse and moderately cellular glial tumour**: This is characteristic of diffuse astrocytomas.
2. **Round or polyclonal nuclei with compact, uniform chromatin**: This suggests a low-grade glioma.
3. **Scant eosinophilic cytoplasm with short processes creating a reticular pattern**: This is typical of astrocytic tumours.
4. **Entrapped neurons**: This can be seen in diffuse gliomas.
5. **Vascular infrastructure without notable endothelial thickening or microvascular proliferation**: This indicates a lower grade tumour, as higher grade gliomas often show microvascular proliferation.
6. **Rare mitotic figures**: This further supports a low-grade tumour.

Immunohistochemistry findings:
- **Diffuse GFAP expression**: Confirms glial origin.
- **Positive IDH1 (R132H) expression**: Indicates an IDH1 mutation, common in diffuse astrocytomas.
- **Absent ATRX expression**: Indicates a mutation, which is often seen in IDH-mutant astrocytomas.
- **Very low Ki67 proliferation index (<1%)**: Supports a low-grade tumour.

Combining these histological and immunohistochemical features, the diagnosis of a **diffuse astrocytoma, IDH-mutant (WHO Grade II)**, is the most fitting.

ChatGPT RAG response:

Based on the provided histopathological description and immunohistochemical findings, the diagnosis appears to be consistent with **Astrocytoma, IDH-mutant, CNS WHO grade 2**. Here is the rationale and references from the WHO guidelines:

Diagnostic Features and Rationale:
1. **Histopathology**:
   - **Diffuse and moderately cellular glial tumour**: This indicates a diffusely infiltrating glioma, which is characteristic of astrocytomas.
   - **Tumour cells nuclei exhibit round or polyclonal forms with compact, uniform chromatin**: These are typical features of low-grade astrocytomas.
   - **Scant eosinophilic cytoplasm with short processes creating a reticular pattern**: This morphology aligns with the description of well-differentiated astrocytoma cells.
   - **Occasional maturing neurons entrapped within the tumour**: This is a common finding in diffuse gliomas, including astrocytomas.
   - **Extensive network of vasculature without notable endothelial thickening or microvascular proliferation**: The absence of microvascular proliferation is indicative of a lower grade tumour.
   - **Rare mitotic figures in a substantial tumour specimen**: Low mitotic activity suggests a low-grade (CNS WHO grade 2) astrocytoma.
2. **Immunohistochemistry**:
   - **Diffuse GFAP expression**: This supports the glial origin of the tumour, common in astrocytomas.

- **Positive IDH1 (R132H) expression**: This confirms the presence of an IDH1 mutation, which is a key diagnostic marker for IDH-mutant astrocytomas.
- **Absent ATRX expression in tumour cell nuclei**: Loss of ATRX expression further supports the diagnosis of an IDH-mutant astrocytoma.
- **Very low Ki67 proliferation index (<1%)**: This indicates a low proliferation rate, consistent with a CNS WHO grade 2 astrocytoma.

References from WHO Guidelines:

1. **Histopathology**: "IDH-mutant astrocytomas range from well-differentiated, low-cell-density and slow-growing tumours (CNS WHO grade 2)..."【3†source】.
2. **Immunohistochemistry**:
   - "IDH1 p.R132H immunohistochemistry has become a critical initial test for the classification of gliomas..."【3†source】.
   - "Loss of nuclear ATRX expression in neoplastic cells strongly supports the diagnosis of IDH-mutant astrocytoma..."【3†source】.
   - "The growth fraction as determined by the Ki-67 proliferation index is usually < 4% for CNS WHO grade 2 IDH-mutant astrocytomas..."【3†source】.

Conclusion:

The findings are most consistent with **Astrocytoma, IDH-mutant, CNS WHO grade 2**. The combination of histopathological features and immunohistochemical markers aligns with the characteristics described in the WHO guidelines for this type of tumour.

A3: NH17 936 - A2 IDHmut

The histological specimen features multiple sections of a moderately cellular neoplasm exhibiting mild pleomorphism in the glial component. The tumour cells display rounded to irregular, darkly stained nuclei with indistinct cell borders. The tumour cells are arranged in a patternless manner within a fibrillary background stroma. Mitoses are not easily discernible. Microvascular proliferation or necrosis are not detected in this specimen.

Immunostaining is positive for the presence of mutant IDH1 (R132H) within the tumour cells. ATRX is absent in these cells' nuclei. The Ki67 proliferation index reveals a mild elevation at approximately 5%.

What do you think the diagnosis would be?

GT: astrocytoma, IDH-mutant, CNS WHO grade 2

Llama Response:

Based on the histopathological description, I would diagnose this tumor as a **Diffuse Astrocytoma, IDH-mutant, WHO Grade II**.

The presence of a moderately cellular neoplasm with a fibrillary background, mild pleomorphism, and rounded to irregular nuclei is consistent with an astrocytic tumor. The absence of mitoses, microvascular proliferation, and necrosis suggests a low-grade tumor. The positive staining for mutant IDH1 (R132H) and the loss of ATRX expression are characteristic of IDH-mutant astrocytomas. The Ki67 proliferation index of 5% is also consistent with a low-grade tumor.

The WHO classification of tumors of the central nervous system (2016) recognizes IDH-mutant diffuse astrocytomas as a distinct entity, which is classified as a Grade II tumor.

Llama RAG response:

Based on the histological description and immunostaining results, I believe the diagnosis is an **IDH-mutant astrocytoma, grade 2**.

Here's why:

- The histological description mentions a moderately cellular neoplasm with mild pleomorphism in the glial component, which is consistent with an astrocytic tumour.
- The patternless arrangement of tumour cells within a fibrillary background stroma is also consistent with an astrocytic tumour.
- The presence of mutant IDH1 (R132H) in the tumour cells, as detected by immunostaining, is a hallmark of IDH-mutant astrocytomas (pg. 5, "Introduction to CNS Tumours" and pg. 10, "CNS Tumour Nomenclature" of the attached document).
- The absence of ATRX protein in the tumour cells' nuclei is also consistent with an IDH-mutant astrocytoma.

- The Ki67 proliferation index of approximately 5% is relatively low, which suggests a low-grade tumour.
- Given the presence of mutant IDH1 and the relatively low Ki67 proliferation index, I would grade this tumour as a grade 2 astrocytoma, according to the grading system outlined in the attached document (pg. 12, "CNS Tumour Grading").

Therefore, the diagnosis is an IDH-mutant astrocytoma, grade 2.

Claude Response:

Based on the histopathological description and immunostaining results provided, the diagnosis is most likely:

Diffuse Astrocytoma, IDH-mutant, WHO grade 2

Reasoning:
1. Histology:
   - Moderately cellular neoplasm
   - Mild pleomorphism in the glial component
   - Tumour cells with rounded to irregular, darkly stained nuclei
   - Patternless arrangement in a fibrillary background
   - Absence of easily discernible mitoses
   - No microvascular proliferation or necrosis
2. Immunohistochemistry:
   - Positive for IDH1 (R132H) mutation
   - Loss of ATRX nuclear expression
   - Ki67 proliferation index mildly elevated at approximately 5%

The presence of an IDH1 mutation and loss of ATRX expression are characteristic of diffuse astrocytomas. The absence of significant mitotic activity, microvascular proliferation, and necrosis, along with only mild pleomorphism and a relatively low Ki67 index, support a WHO grade 2 classification rather than a higher grade.

This diagnosis aligns with the 2021 WHO Classification of Tumors of the Central Nervous System, which emphasizes the importance of both histological features and molecular markers in the diagnosis and grading of gliomas.

Would you like me to elaborate on any aspect of this diagnosis or provide additional information?

Claude RAG response:

Based on the histopathological description and immunostaining results provided, and referring exclusively to the WHO diagnostic guidelines document, I believe the diagnosis is:

Astrocytoma, IDH-mutant, CNS WHO grade 3

Here's my reasoning, with references to the document:

1. IDH mutation: The tumour is positive for IDH1 (R132H) mutation. This is a key diagnostic feature of IDH-mutant astrocytomas, as stated in the document: "Astrocytoma, IDH-mutant, is a diffusely infiltrating IDH1- or IDH2-mutant glioma with frequent ATRX and/or TP53 mutation and absence of 1p/19q codeletion (CNS WHO grade 2, 3, or 4)."
2. ATRX status: The document notes that "Inactivating ATRX alterations commonly co-occur with TP53 mutations in IDH-mutant astrocytomas. These often result in a truncated protein and abrogated protein expression, leading to loss of nuclear ATRX immunoreactivity." The absence of ATRX in the nuclei of tumour cells in this case supports the diagnosis of IDH-mutant astrocytoma.
3. Histological features: The description of a "moderately cellular neoplasm exhibiting mild pleomorphism in the glial component" with "rounded to irregular, darkly stained nuclei" arranged in a "patternless manner within a fibrillary background stroma" is consistent with the histopathology of astrocytoma described in the document.
4. Grade determination: The document states for CNS WHO grade 3 astrocytomas: "CNS WHO grade 3 astrocytomas are characterised by a diffusely infiltrative astrocytic glioma with an IDH1 or IDH2 mutation that exhibits focal or dispersed anaplasia and displays significant mitotic activity. Microvascular proliferation, necrosis, and homozygous deletions of CDKN2A and/or CDKN2B are absent."

While mitoses are not easily discernible in this case, the Ki67 proliferation index of approximately 5% suggests increased proliferative activity. The document mentions: "In CNS WHO grade 3 tumours, the Ki-67 proliferation index is usually in the range of 4–10%, but it can overlap with values for CNS WHO grade 2 tumours at one end of the range and CNS WHO grade 4 tumours at the other."
The absence of microvascular proliferation and necrosis rules out CNS WHO grade 4, while the increased Ki67 index and moderate cellularity suggest a higher grade than CNS WHO grade 2.

In conclusion, the combination of IDH mutation, loss of ATRX expression, histological features, and the Ki67 proliferation index most closely aligns with the diagnosis of Astrocytoma, IDH-mutant, CNS WHO grade 3 according to the provided WHO diagnostic guidelines.

ChatGPT Response:

Based on the provided histopathological description and immunohistochemical findings, the most likely diagnosis is **diffuse astrocytoma, IDH-mutant (WHO Grade II)**.

Here's the reasoning:
1. **Histopathological features**:

- **Moderately cellular neoplasm**: Suggestive of a low-grade glioma.
- **Mild pleomorphism**: Indicates some variation in the size and shape of cells, typical of diffuse astrocytomas.
- **Rounded to irregular, darkly stained nuclei with indistinct cell borders**: Common in astrocytomas.
- **Patternless arrangement in a fibrillary background stroma**: Characteristic of diffuse astrocytomas.

- **Absence of microvascular proliferation and necrosis**: Rules out higher-grade gliomas (e.g., anaplastic astrocytoma or glioblastoma).

2. **Immunohistochemistry**:
   - **Positive for mutant IDH1 (R132H)**: A key marker for diffuse gliomas, particularly astrocytomas and oligodendrogliomas.
   - **Absence of ATRX**: Suggests a diagnosis of astrocytoma, as ATRX loss is often seen in astrocytomas but not in oligodendrogliomas.
   - **Ki67 proliferation index of approximately 5%**: Indicative of a low to moderate proliferative activity, consistent with a low-grade tumor.

Considering these points, the combination of histological features and immunohistochemical profile strongly supports the diagnosis of a ****diffuse astrocytoma, IDH-mutant (WHO Grade II)****.

ChatGPT RAG response:

Based on the provided histopathological description and the WHO diagnostic guidelines, the likely diagnosis is "Astrocytoma, IDH-mutant, CNS WHO grade 2."

Here’s the reasoning:

1. **Histopathological Features**:
   - **Moderately cellular neoplasm**: This indicates a tumour that is not highly cellular, which is consistent with lower-grade astrocytomas.
   - **Mild pleomorphism**: This mild variation in cell shape and size is typical for lower-grade astrocytomas.
   - **Rounded to irregular, darkly stained nuclei with indistinct cell borders**: This is characteristic of astrocytic tumours.
   - **Patternless arrangement within a fibrillary background**: This suggests a diffusely infiltrating glial tumour.
   - **Absence of mitoses**: This is consistent with a lower-grade tumour.
   - **No microvascular proliferation or necrosis**: These features are absent in CNS WHO grade 2 astrocytomas.
2. **Immunostaining**:
   - **Positive for mutant IDH1 (R132H)**: This mutation is common in lower-grade diffuse astrocytomas.
   - **Absence of ATRX in nuclei**: This suggests a mutation in ATRX, which supports the diagnosis of an IDH-mutant astrocytoma.
   - **Ki67 proliferation index at approximately 5%**: This mild elevation is within the range for CNS WHO grade 2 astrocytomas.
3. **Diagnostic Criteria from the WHO Guidelines**:
   - Astrocytoma IDH-mutant must display features of a diffusely infiltrating glioma, IDH1 or IDH2 mutation, and either loss of nuclear ATRX expression or exclusion of combined whole-arm deletions of 1p and 19q【3†source】.
   - The tumour described fits the essential diagnostic criteria for "Astrocytoma, IDH-mutant, CNS WHO grade 2" due to its well-differentiated nature and lack of histological features of anaplasia, mitotic activity, microvascular proliferation, and necrosis【3†source】.

   - Therefore, the diagnosis is "Astrocytoma, IDH-mutant, CNS WHO grade 2.

A4: NH13 1711 - A2 IDHmut

The specimen is formed of a moderately cellular, diffusely infiltrative tumour of astroglial origin. The tumour cells demonstrate angulated nuclei with mild pleomorphism and are situated within a dense fibrillar stroma that exhibits focal microcystic changes. No mitoses were identified. No necrosis or microvascular proliferation are seen.
On immunohistochemistry, the tumour cells are positive for mutant IDH1 staining. Tumour cells also show strong immunoreactivity for GFAP in the cytoplasm and processes, and MAP2 with ring-like cytoplasmic immunoreactivity. The Ki67 proliferative index is moderate (approximately 5%) across tumour tissue, but low in the cortex fragments with minimal tumour infiltration. ATRX immunoreactivity is lost in the tumour cell nuclei.

What do you think the diagnosis would be?

GT: astrocytoma, IDH-mutant, CNS WHO grade 2

Llama Response:

Based on the histopathological description, I would diagnose this tumor as a **Diffuse Astrocytoma, IDH-mutant, WHO Grade II**.

The presence of a moderately cellular, diffusely infiltrative tumor with angulated nuclei and a dense fibrillar stroma, along with the immunohistochemical profile (positive for GFAP, MAP2, and mutant IDH1, and loss of ATRX immunoreactivity) are all consistent with a diffuse astrocytoma. The low Ki67 proliferative index and lack of mitoses, necrosis, and microvascular proliferation also support a low-grade tumor.

The IDH-mutant status is a key feature of this tumor, and the lack of high-grade features (such as necrosis, microvascular proliferation, and high mitotic activity) suggest a WHO Grade II tumor.

Llama RAG response:

Based on the histopathological description and immunohistochemical results, I believe the diagnosis is **IDH-mutant astrocytoma, Grade 2**.

Here's the reasoning:

- The tumor is of astroglial origin, and the presence of GFAP and MAP2 positivity supports this.
- The tumor cells demonstrate angulated nuclei with mild pleomorphism, which is consistent with a low-grade astrocytoma.
- The absence of necrosis and microvascular proliferation also supports a low-grade tumor.
- The positive mutant IDH1 staining is a key feature of IDH-mutant astrocytomas.
- The loss of ATRX immunoreactivity in the tumor cell nuclei is also consistent with an IDH-mutant astrocytoma.
- The Ki67 proliferative index of approximately 5% is moderate, which is consistent with a Grade 2 tumor.

According to the WHO Classification of Tumours, IDH-mutant astrocytomas are graded based on their molecular characteristics, and the presence of an IDH mutation is a key feature of Grade 2 tumors (Page 10, CNS Tumour Grading).

Therefore, based on the histopathological and immunohistochemical features, I believe the diagnosis is IDH-mutant astrocytoma, Grade 2.

Claude Response:

Based on the histopathological description and immunohistochemistry results provided, the diagnosis is most likely:

Diffuse Astrocytoma, IDH-mutant, WHO grade 2

Reasoning:

1. Histology:
    - Moderately cellular, diffusely infiltrative tumor of astroglial origin
    - Mild nuclear pleomorphism
    - Dense fibrillar stroma with focal microcystic changes
    - Absence of mitoses, necrosis, and microvascular proliferation
2. Immunohistochemistry:
    - Positive for mutant IDH1 (key diagnostic feature)
    - GFAP positive (confirming astroglial origin)
    - MAP2 positive (often seen in diffuse gliomas)
    - Loss of ATRX immunoreactivity (common in IDH-mutant astrocytomas)
    - Moderate Ki67 proliferative index (approximately 5%)
3. Grade:
    - The absence of significant mitotic activity, microvascular proliferation, and necrosis, combined with the moderate cellularity and Ki67 index, supports a WHO grade 2 designation.

The presence of an IDH1 mutation, along with the diffuse growth pattern and astrocytic morphology, classifies this as a diffuse astrocytoma. The loss of ATRX expression further supports this diagnosis, as it is commonly seen in IDH-mutant astrocytomas.

This diagnosis aligns with the 2021 WHO Classification of Tumors of the Central Nervous System, which emphasizes the importance of molecular features (such as IDH mutation status) in the classification of diffuse gliomas.

Claude RAG response:

Based on the histopathological description and immunohistochemistry results provided, and using only the information from the attached WHO diagnostic guidelines document, the diagnosis appears to be:

Astrocytoma, IDH-mutant, CNS WHO grade 2

Reasoning and references from the document:

1. Diffusely infiltrative astroglial tumor: The description matches the definition of astrocytoma, IDH-mutant as "a diffusely infiltrating IDH1- or IDH2-mutant glioma" (Definition section under "Astrocytoma, IDH-mutant").
2. IDH mutation: The tumor cells are positive for mutant IDH1 staining, which is a key diagnostic criterion. The document states: "Astrocytoma, IDH-mutant, must display features of a diffusely infiltrating glioma. In addition, this tumour must have IDH1 codon 132 or IDH2 codon 172 missense mutation" (Essential and desirable diagnostic criteria section).
3. ATRX loss: The loss of ATRX immunoreactivity in tumor cell nuclei supports the diagnosis. The document notes: "loss of nuclear ATRX expression strongly supports the diagnosis of IDH-mutant astrocytoma" (Diagnostic molecular pathology section).
4. Histological features: The description of moderate cellularity, mild pleomorphism, and absence of mitoses, necrosis, and microvascular proliferation is consistent with a CNS WHO grade 2 tumor. The document states: "CNS WHO grade 2 tumours are characterised by a diffusely infiltrative astrocytic glioma with an IDH1 or IDH2 mutation that is well differentiated and lacks histological features of anaplasia. Mitotic activity is not detected or very low." (Grading subsection under Histopathology).
5. Ki67 index: The moderate Ki67 index of approximately 5% is consistent with a grade 2 tumor. The document mentions: "The growth fraction as determined by the Ki-67 proliferation index is usually < 4% for CNS WHO grade 2 IDH-mutant astrocytomas." While slightly higher than 4%, it's not high enough to warrant a grade 3 designation.
6. GFAP positivity: The strong GFAP immunoreactivity supports the astrocytic nature of the tumor, as mentioned in the Immunophenotype section: "Individual tumour cells of IDH-mutant astrocytoma reliably express GFAP, although to varying degrees."

In conclusion, based solely on the information provided in the WHO diagnostic guidelines document, the histological features and molecular profile are most consistent with an Astrocytoma, IDH-mutant, CNS WHO grade 2.

ChatGPT Response:

Based on the histopathological description provided, the tumour described is consistent with an **astrocytoma, IDH-mutant, WHO Grade 2**.

Here are the key points leading to this diagnosis:

1. **Cellular and Structural Characteristics**:
   - Moderately cellular, diffusely infiltrative tumour of astroglial origin.
   - Angulated nuclei with mild pleomorphism.
   - Dense fibrillar stroma with focal microcystic changes.
   - No mitoses, necrosis, or microvascular proliferation.
2. **Immunohistochemical Profile**:
   - Positive for mutant IDH1 staining.
   - Strong immunoreactivity for GFAP (glial fibrillary acidic protein) in the cytoplasm and processes, indicating astrocytic origin.
   - Strong immunoreactivity for MAP2 with ring-like cytoplasmic staining.

- Ki67 proliferative index is moderate (approximately 5%).
- Loss of ATRX immunoreactivity in tumour cell nuclei, which is often associated with astrocytomas.

These features, particularly the absence of high-grade characteristics such as mitoses, necrosis, and microvascular proliferation, combined with the specific immunohistochemical profile, support the diagnosis of an astrocytoma with IDH mutation and Grade 2 according to the World Health Organization (WHO) classification.

ChatGPT RAG response:

Based on the histopathological description provided and using the World Health Organisation Classification of Tumours (5th edition) guidelines, the diagnosis appears to be **Astrocytoma, IDH-mutant, CNS WHO grade 2**.

Here is the reasoning for this diagnosis:
1. **Histological Features**:
   - **Moderately cellular**: Indicates an increase in cellularity compared to normal brain tissue, but not excessively high.
   - **Diffusely infiltrative**: Characteristic of astrocytomas which infiltrate surrounding brain tissue.
   - **Angulated nuclei with mild pleomorphism**: Consistent with astrocytic tumors which can show some variability in nuclear shape and size.
   - **Dense fibrillar stroma with focal microcystic changes**: This is a common finding in lower-grade astrocytomas.
   - **No mitoses identified**: This supports a lower grade (grade 2) astrocytoma.
2. **Absence of High-Grade Features**:
   - **No necrosis or microvascular proliferation**: These features are absent, which are typically seen in higher-grade tumors (grade 3 or 4).
3. **Immunohistochemistry**:
   - **Positive for mutant IDH1**: Confirms the presence of an IDH1 mutation, which is a hallmark of IDH-mutant astrocytomas.
   - **Strong immunoreactivity for GFAP and MAP2**: Indicates astrocytic lineage.
   - **Ki67 proliferative index of approximately 5%**: Suggests a low to moderate proliferative activity, more in line with grade 2 tumors.
   - **Loss of ATRX immunoreactivity**: ATRX loss is typical of IDH-mutant astrocytomas and helps in differentiating from oligodendrogliomas which usually retain ATRX expression.

Based on these findings, the most appropriate diagnosis is ****Astrocytoma, IDH-mutant, CNS WHO grade 2****.

References:
- WHO Classification of Tumours, Central Nervous System Tumours, 5th Edition:
- "Astrocytoma IDH-mutant" section for histopathology, immunophenotype, and grading criteria (refer to document sections on pages related to Astrocytoma IDH-mutant)【3†source】.

A5: NH15 1067 - A2 IDHmut

This specimen contains various sections of grey and white matter, some displaying typical structures while others show an infiltrative tumour with a moderate increase in cellularity. This tumour is formed by atypical astrocytes, characterised by hyperchromatic, elongated nuclei with occasional multinucleated cells. Entrapped neurons are noted amidst the tumour. No mitoses, microvascular proliferation or necrosis were seen.
GFAP immunostaining reveals widespread cytoplasmic and processes staining in the tumour cell population. The mutant IDH1 immunostain was positive, while nuclear ATRX expression was absent within the tumour cells. The Ki67 labelling index was minimal with only sporadic singly positive cells observed."

What do you think the diagnosis would be?

GT: astrocytoma, IDH-mutant, CNS WHO grade 2

Llama Response:

Based on the histopathological description, I believe the diagnosis is a **Diffuse Astrocytoma, specifically a Grade II** tumor.

Here's why:
- The presence of atypical astrocytes with hyperchromatic, elongated nuclei and occasional multinucleated cells suggests an astrocytic tumor.
- The lack of mitoses, microvascular proliferation, and necrosis indicates a low-grade tumor, which is consistent with a Grade II astrocytoma.
- The positive GFAP immunostaining supports the diagnosis of an astrocytic tumor.
- The presence of entrapped neurons amidst the tumor is also consistent with a diffuse astrocytoma.
- The positive mutant IDH1 immunostain and absence of nuclear ATRX expression are also characteristic of a Grade II diffuse astrocytoma.
- The minimal Ki67 labeling index suggests a low proliferative activity, which is consistent with a low-grade tumor.

So, the diagnosis would be a Grade II Diffuse Astrocytoma.

Llama RAG response:

Based on the description, I think the diagnosis is **IDH-mutant astrocytoma, grade 2**.

Here's my reasoning:
- The description mentions an infiltrative tumour with atypical astrocytes, hyperchromatic nuclei, and entrapped neurons, which suggests an astrocytic tumour (Reference: CNS Tumour Nomenclature section, where astrocytomas are discussed).

- The presence of GFAP immunostaining in the tumour cells supports the diagnosis of an astrocytic tumour (Reference: Gene and Protein Nomenclature section, where GFAP is mentioned as a marker for astrocytes).
- The positive mutant IDH1 immunostain indicates that the tumour is IDH-mutant, which is a characteristic of IDH-mutant astrocytomas (Reference: CNS Tumour Grading section, where IDH-mutant astrocytomas are discussed).
- The absence of mitoses, microvascular proliferation, and necrosis suggests a low-grade tumour, which is consistent with a grade 2 astrocytoma (Reference: CNS Tumour Grading section, where grading criteria for astrocytomas are discussed).
- The minimal Ki67 labelling index also supports a low-grade tumour diagnosis.

Putting it all together, I believe the diagnosis is IDH-mutant astrocytoma, grade 2.

Claude Response:

Based on the histopathological description provided, the diagnosis is most likely:

Diffuse Astrocytoma, IDH-mutant, WHO grade 2

Reasoning:

1. The tumor is described as infiltrative with a moderate increase in cellularity, which is characteristic of diffuse gliomas.
2. The tumor cells are described as atypical astrocytes with hyperchromatic, elongated nuclei, suggesting an astrocytic lineage.
3. The absence of mitoses, microvascular proliferation, and necrosis indicates a lower-grade tumor, ruling out high-grade gliomas like glioblastoma.
4. GFAP immunostaining is positive, confirming the astrocytic nature of the tumor.
5. The tumor is IDH-mutant, as evidenced by the positive mutant IDH1 immunostain. This is a key molecular feature of lower-grade diffuse gliomas.
6. Loss of ATRX expression (absent nuclear ATRX staining) is common in IDH-mutant astrocytomas.
7. The Ki67 labeling index is minimal, indicating a low proliferation rate, which is consistent with a grade 2 tumor.
8. The presence of entrapped neurons within the tumor is typical of diffuse gliomas.

Given these features, particularly the IDH mutation, astrocytic morphology, and absence of high-grade features, the most likely diagnosis is a Diffuse Astrocytoma, IDH-mutant, WHO grade 2. This classification is based on the 2021 WHO Classification of Tumors of the Central Nervous System.

Claude RAG response:

Based on the histopathological description provided and using only the information from the attached WHO diagnostic guidelines document, the most likely diagnosis is:

Astrocytoma, IDH-mutant, CNS WHO grade 2

Here's the reasoning behind this diagnosis, referencing the relevant sections of the document:

1. The tumor is described as infiltrative with atypical astrocytes, which aligns with the definition of astrocytoma, IDH-mutant as "a diffusely infiltrating IDH1- or IDH2-mutant glioma" (see "Definition" under "Astrocytoma, IDH-mutant").
2. The presence of IDH1 mutation is confirmed by the positive mutant IDH1 immunostain, which is a key diagnostic criterion for IDH-mutant astrocytoma (see "Diagnostic molecular pathology" section under "Astrocytoma, IDH-mutant").
3. The loss of nuclear ATRX expression in tumor cells is described as a common feature of IDH-mutant astrocytomas (see "Diagnostic molecular pathology" section).
4. The histological features described, including moderate increase in cellularity, atypical astrocytes with hyperchromatic nuclei, and absence of mitoses, microvascular proliferation, or necrosis, are consistent with a CNS WHO grade 2 tumor (see "Histopathology" and "Grading" sections under "Astrocytoma, IDH-mutant").
5. The Ki67 labeling index is described as minimal, which is consistent with the statement that "The growth fraction as determined by the Ki-67 proliferation index is usually < 4% for CNS WHO grade 2 IDH-mutant astrocytomas" (see "Proliferation" subsection).
6. The widespread GFAP immunostaining in tumor cells is also consistent with the description of IDH-mutant astrocytomas (see "Immunophenotype" section).

This diagnosis meets the essential diagnostic criteria outlined in the document for Astrocytoma, IDH-mutant:
- Features of a diffusely infiltrating glioma
- IDH1 mutation (confirmed by immunostaining)
- Loss of nuclear ATRX expression

The grade 2 designation is supported by the lack of anaplastic features such as significant mitotic activity, microvascular proliferation, or necrosis, which would be required for higher grades (see "Grading" section).

ChatGPT Response:

Based on the histopathological description provided, the diagnosis is most likely **Diffuse Astrocytoma, IDH-mutant** (WHO Grade II).

Here are the key features that support this diagnosis:
1. **Cellularity and Atypical Astrocytes**:
   - The tumour shows a moderate increase in cellularity with atypical astrocytes, which are characteristic of diffuse astrocytomas.
2. **Nuclear Features**:
   - Hyperchromatic, elongated nuclei with occasional multinucleated cells are often seen in astrocytomas.
3. **Entrapped Neurons**:
   - The presence of entrapped neurons amidst the tumour is a common finding in infiltrative gliomas like diffuse astrocytomas.
4. **Lack of Mitotic Activity, Microvascular Proliferation, and Necrosis**:

- The absence of these features suggests a lower grade tumour, specifically WHO Grade II. Higher grade gliomas (e.g., anaplastic astrocytoma or glioblastoma) would typically show mitoses, microvascular proliferation, and/or necrosis.

5. **Immunohistochemistry**:
   - **GFAP (Glial Fibrillary Acidic Protein)**: Positive staining supports the glial origin of the tumour.
   - **IDH1 Mutation**: Positivity indicates an IDH-mutant astrocytoma, which is a favourable prognostic marker compared to IDH-wildtype.
   - **ATRX Loss**: The absence of ATRX expression is frequently seen in IDH-mutant astrocytomas.
   - **Ki67 Labelling Index**: A low proliferative index (Ki67 labelling index minimal with only sporadic positive cells) aligns with a lower grade tumour (Grade II).

Together, these findings are consistent with a diagnosis of **Diffuse Astrocytoma, IDH-mutant (WHO Grade II)**.

ChatGPT RAG response:

Based on the histopathological description provided and using the WHO diagnostic guidelines for neuropathological tumours, the most likely diagnosis is "Astrocytoma, IDH-mutant, CNS WHO grade 2". Here is a detailed breakdown of how this conclusion was reached:

1. **Tumor Characteristics**:
   - **Infiltrative Tumor**: The description mentions an infiltrative tumor with moderate cellularity increase, which is characteristic of diffuse astrocytomas.
   - **Atypical Astrocytes**: Presence of hyperchromatic, elongated nuclei and occasional multinucleated cells aligns with astrocytoma characteristics.
2. **Immunohistochemistry**:
   - **GFAP Positive**: The widespread GFAP immunostaining supports the astrocytic nature of the tumor cells.
   - **Mutant IDH1 Positive**: Indicates the presence of an IDH1 mutation, which is a key marker for IDH-mutant astrocytomas.
   - **Nuclear ATRX Loss**: Loss of ATRX expression is typically associated with IDH-mutant astrocytomas, helping differentiate from oligodendrogliomas which retain ATRX expression.
3. **Additional Features**:
   - **Ki-67 Labeling Index**: The minimal Ki-67 labeling index suggests low proliferative activity, supporting a lower grade tumor.
   - **Absence of Mitoses, Microvascular Proliferation, and Necrosis**: The lack of these features is consistent with CNS WHO grade 2 astrocytomas, as higher grades would show these features.
4. **WHO Guidelines Alignment**:
   - According to the WHO guidelines, CNS WHO grade 2 astrocytomas are characterized by a diffusely infiltrative astrocytic glioma with an IDH1 or IDH2 mutation that is well-differentiated and lacks histological features of anaplasia such as significant mitotic activity, microvascular proliferation, or necrosis【3†source】.

Thus, the diagnosis based on the given information and WHO guidelines would be "**Astrocytoma, IDH-mutant, CNS WHO grade 2**".

A6: NH19 925 - A3 IDHmut

Examination of tissue samples reveals a widespread presence of glial tumour, predominantly situated within the white matter and extending into the bordering cortex. The tumour is significantly disrupted by multiple areas of acute haemorrhage, both within the lesion and its surrounding parenchyma. The tumour cells display a combination of oligodendrocytic-like cells with rounded morphology and astroglial cells with astrocytic or stellate shapes on a background matrix that is variably fibrillary. Focally, there is a transition to hypercellular tumour with increased pleomorphism and occasional mitoses are detectable in these areas. Microvascular proliferation and necrosis are absent from this lesion.

Immunohistochemically, GFAP is extensively expressed within the tumour. However, both IDH1 (R132H) and ATRX are technically unsatisfactory. Further testing has been requested. The ki67 index is moderately elevated, up to 10% in the areas of increased cellularity.

Molecular pathology results show an IDH1 C394T mutation and ATRX mutation. IDH2, TERT and H3F3 mutations were not detected and there was no loss of heterozygosity of 1p19q.

What do you think the diagnosis would be?

GT: astrocytoma, IDH-mutant, CNS WHO grade 3

Llama Response:

Based on the histopathological description and molecular pathology results, I believe the diagnosis is an **Anaplastic Oligoastrocytoma, Grade III**.

Here's my reasoning:

- The presence of oligodendrocytic-like cells and astroglial cells with astrocytic or stellate shapes, along with a fibrillary background matrix, suggests a mixed glioma.
- The focal areas of increased cellularity, pleomorphism, and occasional mitoses are consistent with an anaplastic tumor.
- The absence of microvascular proliferation and necrosis is not typical of a glioblastoma (WHO Grade IV), which would be the most likely alternative diagnosis.
- The immunohistochemical expression of GFAP supports a glial origin.
- The IDH1 C394T mutation and ATRX mutation, in the absence of other mutations (IDH2, TERT, H3F3) and loss of heterozygosity of 1p19q, are consistent with an anaplastic oligoastrocytoma.
- The ki67 index of up to 10% in areas of increased cellularity is also consistent with an anaplastic tumor.

Given the molecular profile and histopathological features, I believe the diagnosis is an Anaplastic Oligoastrocytoma, Grade III.

Llama RAG response:

Based on the provided histopathological description and molecular pathology results, I think the diagnosis would be **IDH-mutant astrocytoma, grade 3**.

Here's the reasoning:

1. The tumour's location in the white matter and cortex, along with the presence of glial cells, suggests a glial tumour (page 2, "Introduction to CNS Tumours").
2. The combination of oligodendrocytic-like and astroglial cells, along with the variably fibrillary background matrix, is consistent with an astrocytic tumour (page 2, "Introduction to CNS Tumours").
3. The presence of acute haemorrhage, focal hypercellularity, and elevated ki67 index (up to 10%) suggest a higher-grade tumour (page 5, "Grading within Types").
4. The IDH1 C394T mutation is a key molecular feature of IDH-mutant astrocytomas (page 7, "Combined histological and molecular grading").
5. The ATRX mutation is also consistent with an IDH-mutant astrocytoma (page 7, "Combined histological and molecular grading").
6. The absence of IDH2, TERT, and H3F3 mutations, as well as no loss of heterozygosity of 1p19q, further supports the diagnosis (page 7, "Combined histological and molecular grading").
7. Given the histological features, molecular profile, and grading considerations, I would assign a grade 3 to this tumour (page 5, "Grading within Types").

Please note that this diagnosis is based solely on the provided information and the attached WHO classification document.

Claude Response:

Based on the histopathological description and molecular findings provided, the most likely diagnosis is:

Anaplastic Astrocytoma, IDH-mutant, WHO grade 3

Here's the reasoning behind this diagnosis:

1. Histology:
   - The tumor shows a mixture of oligodendrocytic-like and astroglial cells.
   - There are areas of increased cellularity, pleomorphism, and occasional mitoses.
   - Importantly, there's an absence of microvascular proliferation and necrosis, which would be indicative of a grade 4 tumor (glioblastoma).
2. Immunohistochemistry:
   - Extensive GFAP expression supports the astrocytic nature of the tumor.
   - The Ki-67 index is moderately elevated (up to 10%), suggesting an increased proliferation rate consistent with a grade 3 tumor.
3. Molecular findings:
   - IDH1 C394T mutation is present, which is characteristic of IDH-mutant gliomas.
   - ATRX mutation is detected, which is commonly associated with astrocytic tumors.
   - Absence of 1p19q codeletion rules out an oligodendroglioma.
   - No TERT promoter mutation, which is more commonly seen in glioblastomas or oligodendrogliomas.

The combination of astrocytic morphology, increased cellularity and mitotic activity, moderately elevated Ki-67 index, presence of IDH1 mutation, ATRX mutation, and absence of 1p19q codeletion all point towards an Anaplastic Astrocytoma, IDH-mutant, WHO grade 3.

This diagnosis aligns with the 2021 WHO Classification of Tumors of the Central Nervous System, which emphasizes the importance of both histological and molecular features in glioma classification.

Claude RAG response:

Based on the histopathological description and molecular findings provided, and referring to the WHO diagnostic guidelines in the attached document, the diagnosis appears to be:

Astrocytoma, IDH-mutant, CNS WHO grade 3

Reasoning:

1. Diffuse growth pattern: The description states "widespread presence of glial tumour, predominantly situated within the white matter and extending into the bordering cortex," which is consistent with a diffusely infiltrating glioma as described in the WHO guidelines for astrocytoma, IDH-mutant (Definition section).
2. IDH mutation: The molecular pathology results show an IDH1 C394T mutation, which is a key diagnostic criterion for IDH-mutant astrocytoma. The WHO guidelines state that astrocytoma, IDH-mutant must have "IDH1 codon 132 or IDH2 codon 172 missense mutation" (Essential and desirable diagnostic criteria section).
3. ATRX mutation: The molecular pathology results indicate an ATRX mutation, which is another characteristic feature of IDH-mutant astrocytoma. The WHO guidelines mention that "Inactivating ATRX alterations commonly co-occur with TP53 mutations in IDH-mutant astrocytomas" (Diagnostic molecular pathology section).
4. Absence of 1p/19q codeletion: The results show no loss of heterozygosity of 1p19q, which helps distinguish this tumor from oligodendroglioma. The WHO guidelines state that IDH-mutant astrocytoma has an "absence of 1p/19q codeletion" (Definition section).
5. Grade 3 features: The description mentions "Focally, there is a transition to hypercellular tumour with increased pleomorphism and occasional mitoses are detectable in these areas." This, combined with the moderately elevated Ki-67 index of up to 10%, suggests a grade 3 tumor. The WHO guidelines state for CNS WHO grade 3 astrocytomas: "Cellularity is mildly to moderately increased compared with that of normal brain, and mild nuclear atypia is characteristic" and "In CNS WHO grade 3 tumours, the Ki-67 proliferation index is usually in the range of 4–10%" (Histopathology section).
6. Absence of grade 4 features: The description explicitly states that "Microvascular proliferation and necrosis are absent from this lesion," which rules out a grade 4 diagnosis according to the WHO guidelines.

Therefore, based on the diffuse growth pattern, IDH1 mutation, ATRX mutation, absence of 1p/19q codeletion, and histological features consistent with grade 3, the most appropriate diagnosis is Astrocytoma, IDH-mutant, CNS WHO grade 3.

ChatGPT Response:

Based on the histopathological and molecular findings described, the tumour is most likely a **Diffuse Astrocytoma, IDH-mutant (Grade II)**.

Here is the reasoning:
1. **Histopathological Characteristics**:
- The tumour is described as glial and located within the white matter extending into the cortex, which is typical for gliomas.
- The presence of a mix of oligodendrocytic-like cells and astrocytic cells suggests a glial origin, specifically within the astrocytic lineage due to the prominent astroglial component.
- No microvascular proliferation or necrosis is noted, which are criteria for higher-grade gliomas (e.g., Grade III Anaplastic Astrocytoma or Grade IV Glioblastoma).
- Increased pleomorphism and occasional mitoses without high mitotic activity or necrosis are consistent with Grade II rather than a higher-grade glioma.

2. **Immunohistochemical Findings**:
- Extensive GFAP expression supports the glial origin.
- The Ki-67 index up to 10% indicates a low to moderate proliferative activity, aligning more with a Grade II tumour.

3. **Molecular Pathology**:
- The presence of an IDH1 mutation (C394T) supports the classification as an IDH-mutant glioma.
- The absence of 1p/19q co-deletion rules out an oligodendroglioma.
- ATRX mutation supports astrocytic lineage as ATRX loss/mutation is typically seen in astrocytomas.
- The absence of TERT and H3F3 mutations further supports that this is not a higher-grade glioma like Glioblastoma.

Diagnosis: **Diffuse Astrocytoma, IDH-mutant (WHO Grade II)**

This classification aligns with the 2021 WHO Classification of Tumours of the Central Nervous System, where the presence of IDH mutations and the specific histopathological and immunohistochemical features are critical for diagnosis and grading.

ChatGPT RAG response:

Based on the histopathological description and molecular pathology results provided, the tumor appears to be an **astrocytoma, IDH-mutant, CNS WHO grade 3**. Here is the detailed reasoning based on the WHO guidelines:

Histopathological Features:
1. **Location**: The tumor is predominantly situated within the white matter and extends into the bordering cortex, typical for diffuse gliomas.
2. **Cell Morphology**: The presence of both oligodendrocytic-like cells and astrocytic cells with stellate shapes suggests a diffuse glioma with mixed features.
3. **Mitoses**: The occasional mitoses in hypercellular areas are consistent with higher-grade astrocytomas.
4. **Microvascular Proliferation and Necrosis**: The absence of these features rules out glioblastoma (CNS WHO grade 4).

5. **Ki-67 Index**: A moderately elevated Ki-67 index up to 10% is consistent with CNS WHO grade 3 astrocytomas.

Immunohistochemistry and Molecular Pathology:
1. **GFAP**: Extensive GFAP expression is characteristic of astrocytic tumors.
2. **IDH1 and ATRX**: The molecular pathology results showing an IDH1 C394T mutation and ATRX mutation confirm the diagnosis of an IDH-mutant astrocytoma. Although the IDH1 (R132H) and ATRX immunohistochemical tests were technically unsatisfactory, the molecular results are definitive.
3. **1p/19q Codeletion**: The absence of 1p/19q codeletion confirms that the tumor is not an oligodendroglioma, IDH-mutant, and 1p/19q-codeleted.
4. **Other Mutations**: The absence of IDH2, TERT, and H3F3 mutations aligns with the diagnosis of an IDH-mutant astrocytoma.

Diagnosis and Grading:
According to the WHO guidelines:
- **Astrocytoma, IDH-mutant CNS WHO grade 2**: Characterized by low mitotic activity and the absence of microvascular proliferation and necrosis.
- **Astrocytoma, IDH-mutant CNS WHO grade 3**: Characterized by significant mitotic activity and the absence of microvascular proliferation and necrosis.

Given the histological description of occasional mitoses and increased cellularity with pleomorphism, along with a Ki-67 index up to 10%, the tumor fits the criteria for **CNS WHO grade 3** rather than grade 2. The definitive molecular findings of IDH1 and ATRX mutations support the classification as an IDH-mutant astrocytoma.

Reference from the WHO Document:
Astrocytoma, IDH-mutant CNS WHO grade 3: Defined as a diffusely infiltrative astrocytic glioma with an IDH1 or IDH2 mutation that exhibits focal or dispersed anaplasia and displays significant mitotic activity, with microvascular proliferation, necrosis, and homozygous deletions of CDKN2A and/or CDKN2B being absent【3†source】.

Thus, the diagnosis is **astrocytoma, IDH-mutant, CNS WHO grade 3**.

A7: NH14 1712 - A3 IDHmut

This brain tissue specimen consists of a mixture of cortex and white matter infiltrated by a diffuse astrocytic neoplasm characterised by hyperchromatic angulated nuclei with a background of fibrillary matrix. Evidence of perineuronal satellitosis is observed among tumour cells. Occasional mitotic figures are seen, however some regions exhibit increased cellularity with marked nuclear pleomorphism and heightened mitotic activity. Although numerous blood vessels are present, no unequivocal vascular endothelial proliferation or tumour necrosis has been identified. The Ki67 proliferative index is moderate (approximately 20%). Mutant IDH1 is positive and ATRX is absent in tumour nuclei.

What do you think the diagnosis would be?

GT: astrocytoma, IDH-mutant, CNS WHO grade 3

Llama Response:

What a great question!

Based on the histopathological description, I think the diagnosis would be:

****Anaplastic Astrocytoma, IDH-mutant, Grade III****

Here's my reasoning:

- The description of a diffuse astrocytic neoplasm with hyperchromatic angulated nuclei and a fibrillary matrix is consistent with an astrocytic tumor.
- The presence of perineuronal satellitosis suggests that the tumor is an astrocytoma rather than an oligodendroglioma.
- The moderate Ki67 proliferative index (approximately 20%) and the presence of occasional mitotic figures suggest a higher-grade tumor.
- The absence of vascular endothelial proliferation and tumor necrosis suggests that the tumor is not a glioblastoma (Grade IV).
- The presence of mutant IDH1 and absence of ATRX in tumor nuclei are consistent with an IDH-mutant astrocytoma.
- The increased cellularity, nuclear pleomorphism, and heightened mitotic activity in some regions suggest a higher-grade tumor, consistent with an anaplastic astrocytoma (Grade III).

Please let me know if I'm correct!

Llama RAG response:

Based on the histopathological description, I believe the diagnosis is an **IDH-mutant astrocytoma, grade 3**.

Here's my reasoning, supported by references to the attached document:

- The description of "hyperchromatic angulated nuclei" and "fibrillary matrix" suggests an astrocytic neoplasm (p. 2, CNS Tumour Nomenclature).
- The presence of "perineuronal satellitosis" is consistent with an astrocytoma (p. 2, CNS Tumour Nomenclature).
- The "moderate Ki67 proliferative index (approximately 20%)" indicates a higher grade tumour (p. 5, Grading within Types).
- The presence of "mitotic figures" and "increased cellularity with marked nuclear pleomorphism and heightened mitotic activity" in some regions also support a higher grade tumour (p. 5, Grading within Types).
- The absence of "vascular endothelial proliferation" and "tumour necrosis" suggests that the tumour is not a glioblastoma (p. 5, Grading within Types).
- The positivity for "mutant IDH1" and negativity for "ATRX" in tumour nuclei further support the diagnosis of an IDH-mutant astrocytoma (p. 6, Combined Histological and Molecular Grading).

Given the above features, I believe the diagnosis is an IDH-mutant astrocytoma, grade 3.

Claude Response:

Based on the histopathological description provided, the most likely diagnosis is:

Diffuse Astrocytoma, IDH-mutant, WHO grade 3 (Anaplastic Astrocytoma)

Here's the reasoning behind this diagnosis:

1. Diffuse astrocytic neoplasm: The description mentions a diffuse astrocytic tumor infiltrating both cortex and white matter.
2. IDH mutation: The tumor is positive for mutant IDH1, which is characteristic of diffuse gliomas.
3. ATRX loss: The absence of ATRX in tumor nuclei is commonly associated with astrocytic tumors, particularly those with IDH mutations.
4. Moderate proliferation: The Ki67 index of approximately 20% suggests a moderate proliferation rate, higher than typical for a grade 2 astrocytoma but not as high as often seen in glioblastomas.
5. Anaplastic features: The description notes areas of increased cellularity, marked nuclear pleomorphism, and heightened mitotic activity. These features suggest progression to a grade 3 tumor.
6. Absence of high-grade features: The lack of unequivocal vascular endothelial proliferation or tumor necrosis argues against a grade 4 diagnosis (glioblastoma).

The combination of diffuse astrocytic morphology, IDH mutation, ATRX loss, and anaplastic features without clear high-grade characteristics is most consistent with an IDH-mutant anaplastic astrocytoma (WHO grade 3).

It's worth noting that the 2021 WHO classification of CNS tumors emphasizes molecular features alongside histological appearance. The IDH mutation and ATRX loss are key molecular features supporting this diagnosis.

Claude RAG response:

Based on the histopathological description provided and using only the information from the attached WHO diagnostic guidelines document, I believe the diagnosis would be:

Astrocytoma, IDH-mutant, CNS WHO grade 3

Here's my reasoning, referencing the relevant sections of the document:

1. The tumor is described as a "diffuse astrocytic neoplasm," which aligns with the definition of astrocytoma, IDH-mutant as "a diffusely infiltrating IDH1- or IDH2-mutant glioma" (see "Definition" under "Astrocytoma, IDH-mutant").
2. The presence of "hyperchromatic angulated nuclei" and "perineuronal satellitosis" are consistent with the histopathological features described for astrocytomas (see "Histopathology" section under "Astrocytoma, IDH-mutant").
3. The tumor is positive for mutant IDH1 and shows loss of ATRX expression, which are key molecular features of IDH-mutant astrocytoma (see "Diagnostic molecular pathology" section under "Astrocytoma, IDH-mutant").
4. The description mentions "increased cellularity with marked nuclear pleomorphism and heightened mitotic activity" in some regions, which suggests progression to a higher grade. This, combined with the moderate Ki67 proliferative index of approximately 20%, supports a grade 3 designation (see "Grading" section under "Astrocytoma, IDH-mutant").
5. The absence of "unequivocal vascular endothelial proliferation or tumour necrosis" rules out a grade 4 designation, as these features are required for CNS WHO grade 4 (see "Grading" section under "Astrocytoma, IDH-mutant").
6. The document states that "CNS WHO grade 3 astrocytomas are characterised by a diffusely infiltrative astrocytic glioma with an IDH1 or IDH2 mutation that exhibits focal or dispersed anaplasia and displays significant mitotic activity" (see "Grading" section), which aligns with the description provided.

Therefore, based on the histological features, immunohistochemical findings, and grading criteria described in the WHO diagnostic guidelines document, the most appropriate diagnosis for this tumor appears to be Astrocytoma, IDH-mutant, CNS WHO grade 3.

ChatGPT Response:

Based on the histopathological description provided, the brain tumour described appears to be a **Diffuse Astrocytoma, IDH-mutant**. Here are the key features supporting this diagnosis:

1. **Diffuse Astrocytic Neoplasm**: Indicates a type of glioma, specifically within the astrocytoma category.
2. **Hyperchromatic Angulated Nuclei with Fibrillary Matrix**: Consistent with astrocytoma histology.
3. **Perineuronal Satellitosis**: A common feature in diffuse astrocytomas.
4. **Mitotic Figures and Regions with Increased Cellularity**: Suggests a higher grade, potentially anaplastic astrocytoma.
5. **No Vascular Endothelial Proliferation or Tumour Necrosis**: Rules out Glioblastoma, which typically shows these features.

6. **Moderate Ki67 Proliferative Index (approximately 20%)**: Indicates an intermediate level of proliferation, supporting a grade higher than Grade II but lower than Grade IV.
7. **Mutant IDH1 Positive and ATRX Absence**: Molecular markers characteristic of IDH-mutant astrocytomas.

Considering these findings, the tumour is best classified as an ****Anaplastic Astrocytoma, IDH-mutant (WHO Grade III)****. This diagnosis is supported by the increased mitotic activity and cellularity without the presence of necrosis or microvascular proliferation, aligning with the features of a WHO Grade III tumour.

ChatGPT RAG response:

Based on the provided histopathological description and referencing the WHO diagnostic guidelines for central nervous system (CNS) tumors, the diagnosis can be made as follows:

1. **Diffuse Astrocytic Neoplasm**: The description mentions a "diffuse astrocytic neoplasm," indicating an astrocytic origin, which infiltrates both the cortex and white matter. The hyperchromatic angulated nuclei with a fibrillary matrix are characteristic of astrocytomas.
2. **Perineuronal Satellitosis**: This feature supports the diagnosis of a diffuse infiltrative tumor, commonly seen in astrocytomas.
3. **Mitotic Activity and Nuclear Pleomorphism**: The presence of occasional mitotic figures, regions with increased cellularity, and marked nuclear pleomorphism suggest a higher grade within the astrocytoma category.
4. **Ki67 Proliferative Index**: A moderate Ki67 index of approximately 20% is indicative of higher proliferative activity, supporting a higher grade tumor.
5. **Molecular Findings**:
   - Positive for mutant IDH1: This suggests the tumor is an IDH-mutant astrocytoma.
   - Loss of ATRX expression: This supports the diagnosis of an IDH-mutant astrocytoma and helps exclude oligodendroglioma (which would typically retain ATRX expression and show 1p/19q codeletion).
6. **Absence of Microvascular Proliferation and Necrosis**: These features are necessary to exclude CNS WHO grade 4 astrocytoma. Their absence supports a grade lower than 4.

Diagnosis:

Astrocytoma, IDH-mutant, CNS WHO Grade 3

Justification:

- **Histopathological Features**: The description fits within the spectrum of IDH-mutant astrocytomas, characterized by infiltrative growth, hyperchromatic nuclei, and perineuronal satellitosis. The absence of microvascular proliferation and necrosis rules out CNS WHO grade 4.
- **Mitotic Activity and Cellular Pleomorphism**: Increased mitotic activity and marked pleomorphism are indicative of a higher grade, specifically CNS WHO grade 3, which is supported by the WHO guidelines.
- **Molecular Characteristics**: The presence of mutant IDH1 and loss of ATRX is consistent with IDH-mutant astrocytoma.

Referencing the WHO Classification of Tumours of the Central Nervous System, 5th Edition:

Astrocytoma, IDH-mutant, CNS WHO Grade 3 is characterized by IDH1 or IDH2 mutation, loss of ATRX expression, increased mitotic activity, and significant anaplasia without microvascular proliferation or necrosis【3†source】.

Thus, the most accurate diagnosis based on the provided information is **Astrocytoma, IDH-mutant, CNS WHO Grade 3**.

A8: NH17 2968 - A4 IDHmut

This brain specimen comprises cortex, white matter and leptomeninges, all of which are infiltrated by a diffuse astroglial tumour. The neoplastic cells demonstrate a moderate degree of pleomorphism, commonly featuring both multinucleated and binucleation forms with elongated and hyperchromatic nuclei. Occasional eosinophilic granular bodies are present, as are areas of neurocalcifications. The mitotic index is elevated. Notably, there is significant microvascular endothelial proliferation and areas of pseudopalisading necrosis.

Immunohistochemistry demonstrates that the malignant cells express mutant IDH1 R132H while nuclear ATRX expression is absent. Molecular testing confirms IDH1 mutation, ATRX mutation and homozygous deletion of CDKN2A.

What do you think the diagnosis would be?

GT: astrocytoma, IDH-mutant, CNS WHO grade 4

Llama Response:

What a fascinating case!
Based on the histopathological description and the immunohistochemical and molecular findings, I would diagnose this tumour as a ****Glioblastoma, IDH-mutant** (WHO Grade IV)**.

Here's why:

1. **Diffuse astroglial tumour** with pleomorphic, multinucleated, and binucleated cells suggests a high-grade glioma.
2. **Elevated mitotic index** and **pseudopalisading necrosis** are characteristic features of glioblastoma.
3. **Microvascular endothelial proliferation** is another hallmark of glioblastoma.
4. **IDH1 R132H mutation** and **ATRX mutation** are common genetic alterations in glioblastoma, IDH-mutant.
5. **Loss of ATRX expression** and **homozygous deletion of CDKN2A** are also consistent with glioblastoma, IDH-mutant.

The presence of **eosinophilic granular bodies** and **neurocalcifications** are not specific to any particular tumour type, but they do not contradict the diagnosis of glioblastoma.

Given the presence of an IDH1 mutation, this tumour would be classified as a glioblastoma, IDH-mutant, which is a distinct molecular subtype of glioblastoma.

Llama RAG response:

Based on the histopathological description and immunohistochemistry results, I believe the diagnosis is **IDH-mutant astrocytoma, grade 4**.

According to the attached document, IDH-mutant astrocytomas are characterized by the presence of IDH1 or IDH2 mutations, which is consistent with the molecular testing results. The absence of ATRX expression also supports this diagnosis (Introduction to CNS Tumours, page 2).

The histopathological features, such as the presence of multinucleated and binucleated cells, eosinophilic granular bodies, and neurocalcifications, are also consistent with an astrocytoma (CNS Tumour Nomenclature, page 3).

The elevated mitotic index, microvascular endothelial proliferation, and areas of pseudopalisading necrosis suggest a high-grade tumour, which is consistent with a grade 4 diagnosis (CNS Tumour Grading, page 5).

The homozygous deletion of CDKN2A, a tumour suppressor gene, is also a characteristic feature of high-grade astrocytomas (Combined Histological and Molecular Grading, page 7).

Therefore, based on the histopathological and molecular features, I believe the diagnosis is IDH-mutant astrocytoma, grade 4.

Claude Response:

Based on the histopathological description and molecular findings provided, the diagnosis is most likely:

Anaplastic Astrocytoma, IDH-mutant, WHO Grade 3

Key features supporting this diagnosis:
1. Diffuse astroglial tumor infiltrating cortex, white matter, and leptomeninges
2. Moderate cellular pleomorphism with multinucleated and binucleated forms
3. Elevated mitotic index
4. Presence of microvascular proliferation and pseudopalisading necrosis
5. IDH1 R132H mutation (both immunohistochemically and molecularly confirmed)
6. Loss of ATRX expression and confirmed ATRX mutation
7. Homozygous deletion of CDKN2A

The presence of microvascular proliferation and pseudopalisading necrosis typically suggests a Grade 4 glioblastoma. However, the confirmed IDH mutation and ATRX loss are more consistent with an astrocytoma lineage. The 2021 WHO classification of CNS tumors emphasizes molecular features for classification, and in this case, the IDH-mutant status is a key defining feature.

The homozygous deletion of CDKN2A is associated with progression to a higher grade in IDH-mutant astrocytomas. This, combined with the elevated mitotic index and presence of microvascular proliferation and necrosis, supports a Grade 3 (anaplastic) designation rather than a lower grade astrocytoma.

It's worth noting that the distinction between Grade 3 and Grade 4 IDH-mutant astrocytomas can sometimes be challenging, and some pathologists might consider this a borderline case. However, based

on the molecular profile and the emphasis on IDH status in current classifications, Anaplastic Astrocytoma, IDH-mutant, WHO Grade 3 is the most appropriate diagnosis.

Claude RAG response:

Based on the histopathological description and immunohistochemistry/molecular findings provided, the diagnosis is:

Astrocytoma, IDH-mutant, CNS WHO grade 4

Rationale:

1. Diffuse infiltration: The description states "all of which are infiltrated by a diffuse astroglial tumour," which aligns with the definition of astrocytoma as a "diffusely infiltrating glioma" (Astrocytoma, IDH-mutant section).
2. IDH mutation: The tumor cells express mutant IDH1 R132H, and molecular testing confirms IDH1 mutation. This is a key diagnostic criterion for IDH-mutant astrocytoma (Astrocytoma, IDH-mutant section, Essential diagnostic criteria).
3. ATRX mutation: Nuclear ATRX expression is absent, and molecular testing confirms ATRX mutation. This is consistent with IDH-mutant astrocytoma (Astrocytoma, IDH-mutant section, Essential diagnostic criteria).
4. High-grade features: The description includes "significant microvascular endothelial proliferation and areas of pseudopalisading necrosis," which are characteristic of CNS WHO grade 4 tumors (Astrocytoma, IDH-mutant section, Grading subsection).
5. CDKN2A homozygous deletion: Molecular testing confirms homozygous deletion of CDKN2A. The document states: "CNS WHO grade 4 astrocytomas are characterised by a diffusely infiltrative astrocytic glioma with an IDH1 or IDH2 mutation that exhibits microvascular proliferation or necrosis or homozygous deletion of CDKN2A and/or CDKN2B, or any combination of these features" (Astrocytoma, IDH-mutant section, Grading subsection).

The combination of IDH mutation, ATRX mutation, microvascular proliferation, necrosis, and CDKN2A homozygous deletion firmly supports the diagnosis of Astrocytoma, IDH-mutant, CNS WHO grade 4. This diagnosis integrates both the histological features and the molecular findings as recommended in the WHO classification.

ChatGPT Response

Based on the histopathological description and molecular findings provided, the diagnosis is most consistent with an **IDH-mutant Astrocytoma, Grade IV** (formerly known as Glioblastoma, IDH-mutant).

Here's the reasoning:

1. **Histopathological Features**:
    - **Diffuse astroglial tumour**: Indicates an astrocytic lineage.
    - **Moderate degree of pleomorphism with multinucleated and binucleated forms**: Suggests a high-grade tumour.

- **Elongated and hyperchromatic nuclei**: Common in high-grade astrocytomas.
- **Eosinophilic granular bodies and neurocalcifications**: Often seen in astrocytomas.
- **Elevated mitotic index**: Indicates a high proliferation rate.
- **Microvascular endothelial proliferation**: A hallmark of high-grade gliomas.
- **Pseudopalisading necrosis**: A classic feature of glioblastoma.

2. **Immunohistochemistry and Molecular Findings**:
   - **Mutant IDH1 R132H expression**: Indicates IDH1 mutation, typical of lower-grade astrocytomas (Grade II and III) and a subset of glioblastomas (IDH-mutant).
   - **Absent nuclear ATRX expression**: Suggests ATRX mutation, commonly seen in IDH-mutant astrocytomas.
   - **Molecular testing confirming IDH1 and ATRX mutations**: Supports the diagnosis of an IDH-mutant astrocytoma.
   - **Homozygous deletion of CDKN2A**: Common in higher-grade astrocytomas.
3. **Grade IV Features**:
   - The presence of significant microvascular proliferation and pseudopalisading necrosis are defining features of Grade IV gliomas, specifically glioblastoma.

Thus, the combination of histopathological features and molecular findings leads to the diagnosis of an **IDH-mutant Astrocytoma, Grade IV**.

ChatGPT RAG response:

Based on the provided histopathological description and the molecular findings, the diagnosis can be determined as follows:

Diagnosis: **Astrocytoma, IDH-mutant, CNS WHO grade 4**

Reasoning:
1. **Histopathological Features**:
   - **Diffuse infiltration**: The tumor infiltrates the cortex, white matter, and leptomeninges, consistent with diffuse astroglial tumors.
   - **Moderate pleomorphism**: The presence of multinucleated and binucleated forms with elongated and hyperchromatic nuclei aligns with the description of higher-grade astrocytomas.
   - **Eosinophilic granular bodies**: These are occasionally present in astrocytomas.
   - **Neurocalcifications**: These are compatible with astrocytomas.
   - **Elevated mitotic index**: Indicates higher-grade tumor.
   - **Microvascular proliferation and pseudopalisading necrosis**: Both are hallmark features of grade 4 astrocytomas.
2. **Immunohistochemical and Molecular Features**:
   - **IDH1 R132H mutation**: Confirms the tumor is an IDH-mutant astrocytoma.
   - **Absence of nuclear ATRX expression**: Correlates with ATRX mutation, common in IDH-mutant astrocytomas.
   - **Homozygous deletion of CDKN2A**: This finding alone elevates the tumor to CNS WHO grade 4 regardless of other histological features.

References:

- The essential diagnostic criteria for Astrocytoma IDH-mutant include features of a diffusely infiltrating glioma with IDH1 codon 132 or IDH2 codon 172 missense mutation and either loss of nuclear ATRX expression or exclusion of combined whole-arm deletions of 1p and 19q【3†source】.
- The presence of microvascular proliferation or necrosis in addition to other features confirms a CNS WHO grade 4 designation【3†source】.
- The homozygous deletion of CDKN2A in an IDH-mutant astrocytoma automatically classifies it as CNS WHO grade 4 even without other grade 4 histological features【3†source】.

Based on these criteria from the WHO classification document, the diagnosis is Astrocytoma, IDH-mutant, CNS WHO grade 4.

A9: NH15 1510 - A4 IDHmut

Histology shows multiple large pieces of CNS tissue which are variably infiltrated by a hypercellular glial tumour. Areas of this tumour display a gemistocytic phenotype, while others areas exhibit a more condensed diffuse growth pattern. Multiple instances of microvascular proliferation and both palisading and geographic necrosis are observed. The immunostaining for mutant IDH1 (R132H) was inconclusive. ATRX expression was lost in all tumour cell nuclei. Ki-67 proliferation index showed pronounced proliferative activity within the poorly differentiated nodules throughout the sample. The tumour area was sent for DNA extraction.

Molecular pathology results showed an IDH2 G515T and ATRX mutations. Mutations of IDH1, H3F3 and TERT were not detected. There was no loss of heterozygosity in 1p19q.

What do you think the diagnosis would be?

GT: astrocytoma, IDH-mutant, CNS WHO grade 4

Llama Response:

What a great challenge!

After analyzing the histopathological description and molecular pathology results, I'm going to take a stab at the diagnosis.

Based on the histological features, I think the tumor is a high-grade glioma, specifically a **Glioblastoma (WHO grade IV)**. The presence of microvascular proliferation, palisading and geographic necrosis, and high Ki-67 proliferation index are all consistent with this diagnosis.

The gemistocytic phenotype in some areas and condensed diffuse growth pattern in others suggest a heterogeneous tumor with different growth patterns, which is not uncommon in glioblastomas.

The molecular pathology results further support this diagnosis. The IDH2 G515T mutation is commonly found in glioblastomas, and the ATRX mutation is also consistent with this diagnosis. The lack of IDH1, H3F3, and TERT mutations and no loss of heterozygosity in 1p19q further support the diagnosis of a glioblastoma.

So, my diagnosis would be: Glioblastoma (WHO grade IV).
Please let me know if I'm correct or not!

Llama RAG response:

Based on the histopathological description and molecular pathology results, I would diagnose this tumour as an **IDH-mutant astrocytoma, grade 4 (Glioblastoma, IDH-mutant)**.

Here's my reasoning:

- The histopathological description mentions a hypercellular glial tumour with gemistocytic and diffuse growth patterns, which is consistent with an astrocytic tumour.
- The presence of microvascular proliferation, palisading necrosis, and high Ki-67 proliferation index suggest a high-grade tumour.
- The loss of ATRX expression in tumour cell nuclei is consistent with an astrocytic tumour.
- The molecular pathology results show an IDH2 G515T mutation, which is a characteristic genetic alteration in IDH-mutant astrocytomas.
- The lack of mutations in IDH1, H3F3, and TERT, and the absence of 1p19q loss of heterozygosity, further support the diagnosis of an IDH-mutant astrocytoma.

According to the WHO Classification of Tumours (fifth edition), an IDH-mutant astrocytoma, grade 4, is characterized by the presence of IDH1 or IDH2 mutations, and often exhibits ATRX loss and high proliferative activity (Ref: CNS Tumour Grading section, page 5).

Please note that this diagnosis is based solely on the information provided in the attached document and the given histopathological description and molecular pathology results.

Claude Response:

Based on the histopathological description and molecular pathology results provided, the diagnosis appears to be:

Glioblastoma, IDH-mutant, WHO grade 4

Reasoning:
1. Histological features:
   - Hypercellular glial tumor
   - Gemistocytic phenotype in some areas
   - Microvascular proliferation
   - Palisading and geographic necrosis
   - High Ki-67 proliferation index
2. Immunohistochemistry:
   - Loss of ATRX expression in tumor cell nuclei
3. Molecular pathology:
   - IDH2 G515T mutation (IDH-mutant)
   - ATRX mutation
   - No 1p19q codeletion
   - No TERT promoter mutation

The presence of microvascular proliferation and necrosis, along with the high proliferation index, strongly suggests a grade 4 glioma. The IDH2 mutation classifies this as an IDH-mutant glioma, which is typically associated with a slightly better prognosis compared to IDH-wildtype glioblastomas.

The loss of ATRX expression and the presence of an ATRX mutation, combined with the absence of 1p19q codeletion, rules out oligodendroglioma and supports the diagnosis of an astrocytic tumor.

While IDH-mutant gliomas can be either grade 2, 3, or 4, the presence of significant necrosis and microvascular proliferation in this case points towards a grade 4 diagnosis, making this an IDH-mutant glioblastoma.

Claude RAG response:

Based on the provided histopathological description and molecular pathology results, and using only the information from the attached WHO diagnostic guidelines document, I believe the diagnosis would be:

Astrocytoma, IDH-mutant, CNS WHO grade 4

Here's the reasoning for this diagnosis, with references to the attached document:

1. Histology:
   - The tumor shows hypercellularity, microvascular proliferation, and both palisading and geographic necrosis. These features are characteristic of a high-grade glioma, specifically a CNS WHO grade 4 tumor (Reference: "CNS WHO grade 4 tumours must manifest necrosis and/or microvascular proliferation in addition to the features of CNS WHO grade 3 lesions").
2. Immunohistochemistry:
   - Loss of ATRX expression in tumor cell nuclei is consistent with an IDH-mutant astrocytoma (Reference: "Inactivating ATRX alterations commonly co-occur with TP53 mutations in IDH-mutant astrocytomas. These often result in a truncated protein and abrogated protein expression, leading to loss of nuclear ATRX immunoreactivity.").
3. Molecular pathology:
   - The presence of an IDH2 G515T mutation confirms that this is an IDH-mutant tumor (Reference: "Astrocytoma, IDH-mutant, is a diffusely infiltrating IDH1- or IDH2-mutant glioma with frequent ATRX and/or TP53 mutation and absence of 1p/19q codeletion (CNS WHO grade 2, 3, or 4).").
   - The ATRX mutation further supports the diagnosis of IDH-mutant astrocytoma.
   - The absence of 1p/19q codeletion rules out oligodendroglioma (Reference: "Diagnosis of oligodendrogliomas requires demonstration of IDH mutation by IDH1 p.R132H immunohistochemistry and/or sequencing of the IDH1 or IDH2 gene, as well as demonstration of 1p/19q codeletion").
4. Grading:
   - The presence of microvascular proliferation and necrosis, along with the high Ki-67 proliferation index, supports a grade 4 designation (Reference: "CNS WHO grade 4 astrocytomas are characterised by a diffusely infiltrative astrocytic glioma with an IDH1 or IDH2 mutation that exhibits microvascular proliferation or necrosis").

In conclusion, the combination of IDH2 mutation, ATRX mutation, absence of 1p/19q codeletion, and histological features of microvascular proliferation and necrosis all point to a diagnosis of Astrocytoma, IDH-mutant, CNS WHO grade 4.

ChatGPT Response:

Based on the provided histopathological and molecular findings, the diagnosis is likely ****glioblastoma, IDH-wildtype (WHO grade 4)****.

Key Features Supporting the Diagnosis:
1. **Histopathological Features**:
   - **Hypercellular glial tumour**: Suggestive of a high-grade glioma.
   - **Gemistocytic phenotype and diffuse growth pattern**: Common in gliomas.
   - **Microvascular proliferation and necrosis (both palisading and geographic)**: These are hallmark features of glioblastoma.
2. **Immunohistochemistry and Molecular Pathology**:
   - **ATRX expression lost**: Indicates a high-grade glioma.
   - **IDH1 (R132H) inconclusive but IDH2 mutation (G515T)**: The presence of an IDH2 mutation, though rare, can still be found in glioblastomas. The absence of IDH1 mutation suggests it is not an IDH-mutant glioma.
   - **High Ki-67 proliferation index**: Indicates high proliferative activity typical of glioblastoma.
   - **No loss of heterozygosity in 1p/19q**: This rules out oligodendroglioma.
   - **Absence of TERT promoter mutation**: While common in glioblastoma, its absence does not exclude the diagnosis.
   - **Absence of H3F3 mutation**: Rules out a histone-mutant glioma, which is more common in pediatric high-grade gliomas.

Summary:
The combination of histological features (necrosis, microvascular proliferation) and molecular findings (ATRX mutation, IDH2 mutation, absence of 1p/19q co-deletion) is characteristic of **glioblastoma, IDH-wildtype**, according to the World Health Organization (WHO) classification of Central Nervous System (CNS) tumors. The IDH2 mutation, though less common, still fits within the spectrum of glioblastomas, as these tumors are predominantly defined by their histopathological features rather than the specific IDH mutation when it comes to IDH-wildtype classification.

ChatGPT RAG response:

Based on the provided histopathological and molecular description, the diagnosis can be made as follows:

Histopathological Features:
- **Hypercellular glial tumour**: Indicates a high-grade glioma.
- **Gemistocytic phenotype**: Often seen in astrocytic tumours.
- **Microvascular proliferation and necrosis**: These are indicative of high-grade malignancy, consistent with WHO grade 4 tumours.
- **Inconclusive IDH1 (R132H) staining**: Requires further molecular testing to identify IDH status.
- **Loss of ATRX expression**: Suggestive of an astrocytic tumour, particularly in the context of IDH mutations.
- **High Ki-67 proliferation index**: Supports a high-grade tumour.

Molecular Pathology Results:
- **IDH2 G515T mutation**: Confirms the tumour is IDH-mutant.

- **ATRX mutation**: Consistent with the loss of ATRX expression seen histologically.
- **No mutations in IDH1, H3F3, and TERT**: Rules out glioblastoma IDH-wildtype and H3-mutant gliomas.
- **No 1p/19q codeletion**: Excludes oligodendroglioma.

Diagnosis:
Given these findings, the tumour is best classified as an **astrocytoma, IDH-mutant, CNS WHO grade 4**. This is supported by the presence of an IDH2 mutation, loss of ATRX expression, and high-grade features such as microvascular proliferation and necrosis.

Supporting References from the WHO Guidelines:
- **IDH-mutant astrocytomas** are characterized by the presence of an IDH1 or IDH2 mutation, and ATRX mutations often co-occur with TP53 mutations (although TP53 status is not provided here)【3†source】.
- **CNS WHO grade 4 astrocytomas** must show features such as microvascular proliferation, necrosis, or CDKN2A/B homozygous deletion【3†source】.
- The **loss of ATRX expression** further supports the diagnosis of an IDH-mutant astrocytoma, as this is a common finding in such tumours【3†source】.
- **High Ki-67 proliferation index** correlates with higher tumour grade【3†source】.

Conclusion:
The most appropriate diagnosis for this tumour, based on the provided histopathological and molecular data, is ****astrocytoma, IDH-mutant, CNS WHO grade 4****. This diagnosis aligns with the observed histological and molecular characteristics described in the WHO classification of CNS tumours.

A10: NH19 2160 - A4 IDHmut

In the presented specimen, we identify numerous tissue sections containing an astrocytic cellular neoplasm. The nuclei of these tumour cells exhibit uniformity in shape and size, with a rounded nuclei and moderate chromatin condensation. The tumour cells either display a gemistocytic appearance or have perinuclear clearing. The tumour stroma is characterised by a fibrillary structure that often forms microcysts. Occasional microvascular proliferations are observed. Areas of necrosis are present throughout and occasionally contain haemosiderin deposits.
On immunohistochemistry, the tumour cells are positive for mutant IDH1 (R132H). Expression of ATRX is absent within the malignant cell nuclei but present in those of endothelial and underlying glial cells. Furthermore, histone H3K27me3 expression remains unaltered, which correlates with the observed loss of ATRX expression. Ki-67 approximates 5-10% in some areas of the tumour. The IHC findings were confirmed on molecular analysis (IDH1 and ATRX mutant, H3 wildtype).

What do you think the diagnosis would be?

GT: astrocytoma, IDH-mutant, CNS WHO grade 4

Llama Response:

What a great question!

Based on the histopathological description, I believe the diagnosis is a ****Diffuse Astrocytoma, IDH-mutant, Grade II****.

Here's why:

- The presence of an astrocytic cellular neoplasm with uniform nuclei and gemistocytic appearance points towards an astrocytoma.
- The fibrillary stroma with microcysts and occasional microvascular proliferations are consistent with a low-grade astrocytoma.
- The presence of areas of necrosis and haemosiderin deposits suggests a more aggressive tumor, but the overall morphology and immunohistochemistry findings suggest a grade II tumor rather than a higher grade.
- The positivity for mutant IDH1 (R132H) and loss of ATRX expression are characteristic of diffuse astrocytomas, particularly those with IDH mutations.
- The Ki-67 index of 5-10% is consistent with a low-grade tumor.
- The molecular analysis confirming the IDH1 mutation, ATRX mutation, and H3 wildtype further supports the diagnosis.

Overall, the combination of morphological and immunohistochemical features, along with the molecular analysis, points towards a Diffuse Astrocytoma, IDH-mutant, Grade II.

Llama RAG response:

Based on the provided histopathological description, I believe the diagnosis is an **IDH-mutant astrocytoma, grade 2**.

Here's the reasoning:

1. The description of the tumour cells as having uniform nuclei with moderate chromatin condensation, gemistocytic appearance, and perinuclear clearing is consistent with an astrocytic tumour (Page 2, CNS Tumour Nomenclature).
2. The presence of a fibrillary stroma with microcysts and occasional microvascular proliferations supports the diagnosis of an astrocytoma (Page 2, CNS Tumour Nomenclature).
3. The presence of necrosis with haemosiderin deposits is indicative of a higher-grade tumour, but the overall description suggests a relatively well-differentiated tumour (Page 5, Grading within Types).
4. The immunohistochemical profile, including positivity for mutant IDH1 (R132H), loss of ATRX expression, and unaltered histone H3K27me3 expression, is consistent with an IDH-mutant astrocytoma (Page 7, Combined histological and molecular grading).
5. The Ki-67 index of 5-10% suggests a relatively low proliferative activity, which is consistent with a lower-grade tumour (Page 5, Grading within Types).
6. The molecular analysis confirming IDH1 and ATRX mutations and H3 wildtype further supports the diagnosis of an IDH-mutant astrocytoma.

Based on the above, I would diagnose this tumour as an IDH-mutant astrocytoma, grade 2, which is in line with the WHO classification guidelines (Page 5, Grading within Types).

Please let me know if I'm correct or if I've missed something!

Claude Response:

Based on the histopathological description and immunohistochemistry findings, the diagnosis is most likely:

Diffuse Astrocytoma, IDH-mutant, WHO grade 2

Here's the rationale:

1. Astrocytic cellular neoplasm: The description indicates an astrocytic tumor.
2. Nuclear features: Uniformity in shape and size with rounded nuclei suggests a lower-grade tumor.
3. Cellular features: Gemistocytic appearance and perinuclear clearing are consistent with astrocytoma.
4. Stroma: Fibrillary structure with microcysts is typical of diffuse astrocytoma.
5. Microvascular proliferation: Occasional presence is noted, which can be seen in grade 2 astrocytomas, though more common in higher grades.
6. Necrosis: While present, it's not described as extensive, which is more consistent with a grade 2 rather than a grade 3 or 4 tumor.
7. IDH1 mutation: Positive for R132H mutation, which is characteristic of diffuse gliomas.
8. ATRX loss: Absent expression in tumor cells is typical of IDH-mutant astrocytomas.
9. H3K27me3: Unaltered expression, consistent with ATRX loss and IDH mutation.
10. Ki-67: 5-10% proliferation index is more consistent with a grade 2 tumor.

11. Molecular confirmation: IDH1 and ATRX mutations with H3 wildtype status confirm the diagnosis.

The presence of some microvascular proliferation and areas of necrosis might raise consideration for a grade 3 tumor (anaplastic astrocytoma). However, the overall description, including nuclear uniformity and the Ki-67 index, is more consistent with a grade 2 diffuse astrocytoma. If there's any doubt, additional review or molecular testing might be warranted to definitively rule out a higher grade.

Claude RAG response:

Based on the histopathological description and immunohistochemistry results provided, the diagnosis would be:

Astrocytoma, IDH-mutant, CNS WHO grade 3

Reasoning and references to the WHO diagnostic guidelines:

1. Astrocytic nature: The description mentions "an astrocytic cellular neoplasm" with fibrillary stroma, which is consistent with astrocytoma (Reference: "Astrocytoma, IDH-mutant" section).
2. IDH mutation: The tumor cells are positive for mutant IDH1 (R132H) on immunohistochemistry, confirmed by molecular analysis. This is a key feature of IDH-mutant astrocytomas (Reference: "Diagnostic molecular pathology" subsection under "Astrocytoma, IDH-mutant").
3. ATRX loss: The absence of ATRX expression in tumor cell nuclei, while present in endothelial and glial cells, is characteristic of IDH-mutant astrocytomas (Reference: "Diagnostic molecular pathology" subsection).
4. Histological features: The description mentions uniformity in nuclear shape and size, gemistocytic appearance, and microcystic changes, which are consistent with astrocytoma (Reference: "Histopathology" subsection).
5. Grade determination: The presence of occasional microvascular proliferations and areas of necrosis, along with a Ki-67 index of 5-10% in some areas, suggests a higher grade. These features are more consistent with CNS WHO grade 3 (Reference: "Grading" subsection under "Astrocytoma, IDH-mutant").
6. H3 wildtype: The unaltered histone H3K27me3 expression and molecular confirmation of H3 wildtype status help exclude other entities like diffuse midline glioma (Reference: "Essential and desirable diagnostic criteria" subsection).

The combination of IDH mutation, ATRX loss, and the histological features described, including microvascular proliferation and necrosis, support the diagnosis of Astrocytoma, IDH-mutant, CNS WHO grade 3. This diagnosis fulfills the essential diagnostic criteria outlined in the document for IDH-mutant astrocytoma, with features indicating a higher grade within this tumor type.

ChatGPT Response:

Based on the provided histopathological description and immunohistochemical findings, the most likely diagnosis is an ****IDH-mutant Astrocytoma, WHO grade III****.

Key Points Supporting This Diagnosis:
1. **Histopathology:**
   - The tumour is astrocytic with cells showing gemistocytic appearance or perinuclear clearing.
   - The presence of a fibrillary stroma, microcysts, and occasional microvascular proliferation.
   - Areas of necrosis and haemosiderin deposits, which are suggestive of a more aggressive tumour.
2. **Immunohistochemistry:**
   - Positive for mutant IDH1 (R132H), a marker commonly found in lower-grade astrocytomas and oligodendrogliomas.
   - Loss of ATRX expression in tumour cells, which is associated with astrocytic tumours.
   - Histone H3K27me3 expression remains unaltered (H3 wildtype), ruling out high-grade gliomas like H3K27M-mutant gliomas.
   - Ki-67 labeling index of 5-10% indicates a moderate proliferative activity, supportive of a grade III tumour rather than grade II.

Conclusion:
The described features fit well with an IDH-mutant astrocytoma of WHO grade III, considering the cellularity, necrosis, microvascular proliferation, and molecular profile (IDH1 and ATRX mutations). This type of tumour typically shows an intermediate grade of malignancy with more aggressive features than grade II astrocytomas but does not meet the criteria for grade IV (glioblastoma) due to the absence of microvascular proliferation and necrosis typically required for such a classification.

ChatGPT RAG response:
Based on the histopathological description provided and referring to the WHO Classification of Tumours document, the diagnosis can be determined as follows:

Diagnosis
****Astrocytoma, IDH-mutant, CNS WHO Grade 4****

Reasoning
1. **Histopathological Features**:
   - **Astrocytic Cellular Neoplasm**: The description of uniform nuclei with rounded shape and moderate chromatin condensation, along with gemistocytic appearance and perinuclear clearing, aligns with the characteristics of astrocytomas.
   - **Microvascular Proliferation and Necrosis**: Presence of microvascular proliferation and necrosis are critical features. According to the WHO guidelines, the presence of necrosis and/or microvascular proliferation are necessary for the designation of CNS WHO Grade 4 astrocytoma.
2. **Immunohistochemistry Findings**:
   - **IDH1 (R132H) Positive**: Confirms the tumor as IDH-mutant.
   - **ATRX Loss**: Consistent with the diagnosis of IDH-mutant astrocytoma.
   - **H3K27me3 Expression Unaltered**: Indicates H3-wildtype status, which rules out other potential high-grade gliomas like diffuse midline gliomas.
3. **Molecular Analysis**:
   - **IDH1 and ATRX Mutant, H3 Wildtype**: Confirms the molecular characteristics required for IDH-mutant astrocytoma and excludes other differential diagnoses like oligodendroglioma (which requires 1p/19q codeletion).
4. **Ki-67 Proliferation Index**:

- **5-10%**: Indicates a higher proliferative activity, which can be associated with higher-grade tumors but is not the sole determining factor for grading.

Reference from WHO Classification of Tumours
- **Astrocytoma IDH-mutant CNS WHO Grade 4**: "CNS WHO grade 4 tumors must manifest necrosis and/or microvascular proliferation in addition to the features of CNS WHO grade 3 lesions" (page reference not available due to document truncation).

Given the presence of microvascular proliferation, necrosis, and the described histopathological and molecular features, the most fitting diagnosis within the WHO classification system is **Astrocytoma, IDH-mutant, CNS WHO Grade 4**.

Glioblastoma Cases

G1: N19 996 - GBM IDHwt

Pathology shows an infiltrating, hypercellular astrocytic neoplasm with hyperchromatic, elongated nuclei and irregular nuclear membranes. The tumour is mitoticly active with microvascular endothelial proliferation and pseudopalisading necrosis. The tumours cells are negative for IDH1 and retained nuclear ATRX expression. Ki67 expression is high.

What do you think the diagnosis would be?

GT: glioblastoma, IDH-wildtype

Llama Response:

Based on the histopathological description, I would diagnose this tumour as a **Glioblastoma, WHO Grade IV**.

Here's why:
- The description of an infiltrating, hypercellular astrocytic neoplasm with hyperchromatic, elongated nuclei and irregular nuclear membranes is consistent with a high-grade glioma.
- The presence of mitotic activity, microvascular endothelial proliferation, and pseudopalisading necrosis are all features that are typical of Glioblastoma.
- The negativity for IDH1 and retained nuclear ATRX expression suggest that this is not a lower-grade glioma, which would be more likely to have IDH1 mutations.
- The high Ki67 expression is also consistent with a high-grade, rapidly proliferating tumour like Glioblastoma.

Overall, the combination of these features points towards a diagnosis of Glioblastoma, WHO Grade IV.

Llama RAG response:

Based on the provided histopathological description, I believe the diagnosis is **Glioblastoma, IDH-wildtype, WHO grade 4.**

The features that support this diagnosis include:
- Infiltrating, hypercellular astrocytic neoplasm, which is consistent with a high-grade glioma (p. 2 of the attached document).
- Hyperchromatic, elongated nuclei and irregular nuclear membranes, which indicate high nuclear atypia (p. 2).
- Mitotic activity, microvascular endothelial proliferation, and pseudopalisading necrosis, which are all features of high-grade gliomas (p. 2).

- Negative IDH1 staining and retained nuclear ATRX expression, which are consistent with an IDH-wildtype glioblastoma (p. 14).
- High Ki67 expression, which indicates a high proliferative index, consistent with a high-grade glioma (p. 2).

According to the WHO Classification of Tumours, Glioblastoma, IDH-wildtype, is a WHO grade 4 tumour (p. 14).
Please let me know if you have any further questions or if you'd like me to clarify any aspects of the diagnosis.

Claude Response:

Based on the histopathological description provided, the diagnosis is most likely:

Glioblastoma, IDH-wildtype (WHO grade 4)

Key features supporting this diagnosis:
1. Infiltrating, hypercellular astrocytic neoplasm: Indicative of a high-grade astrocytoma.
2. Hyperchromatic, elongated nuclei with irregular nuclear membranes: Typical of high-grade gliomas.
3. Mitotic activity: Suggests rapid cell division, common in high-grade tumors.
4. Microvascular endothelial proliferation: A hallmark feature of glioblastoma.
5. Pseudopalisading necrosis: Another characteristic feature of glioblastoma.
6. IDH1 negative: Indicates IDH-wildtype status, which is more common in primary glioblastomas.
7. Retained nuclear ATRX expression: Consistent with IDH-wildtype glioblastoma (ATRX mutations are more common in IDH-mutant astrocytomas).
8. High Ki67 expression: Indicates a high proliferation rate, typical of glioblastomas.

The combination of these features, particularly the presence of microvascular proliferation and necrosis in an astrocytic tumor, is diagnostic of glioblastoma. The IDH-wildtype status and the absence of other typical lower-grade glioma mutations (like ATRX loss) further support this diagnosis.

Claude RAG response:

Based on the histopathological description provided and using only the information from the attached WHO diagnostic guidelines document, the diagnosis appears to be:

Glioblastoma, IDH-wildtype (CNS WHO grade 4)

Here's the rationale for this diagnosis, with references to the document:
1. Histological features:
- The tumor is described as an "infiltrating, hypercellular astrocytic neoplasm," which aligns with the definition of glioblastoma as a "diffusely infiltrating, highly cellular glioma composed of astrocytic, usually poorly differentiated tumour cells" (Glioblastoma, IDH-wildtype section, Histopathology subsection).

- The presence of "microvascular endothelial proliferation and pseudopalisading necrosis" are characteristic diagnostic features of glioblastoma (same subsection).

2. Molecular features:
- The tumor cells are "negative for IDH1," which is consistent with the IDH-wildtype status required for this diagnosis (Glioblastoma, IDH-wildtype section, Definition).
- "Retained nuclear ATRX expression" is typical in the "vast majority" of IDH-wildtype glioblastomas (Immunophenotype subsection).

3. Proliferation:
- The tumor is described as "mitoticly active" with "high" Ki67 expression, which is consistent with the "readily identifiable" and often "brisk" mitotic activity described for glioblastomas (Histopathology subsection).

4. Grade:
- Glioblastoma, IDH-wildtype is defined as CNS WHO grade 4 (Definition subsection).

The diagnosis of Glioblastoma, IDH-wildtype can be made based on the presence of microvascular proliferation and/or necrosis in an IDH- and H3-wildtype diffuse glioma, as stated in the Essential and desirable diagnostic criteria section: "Glioblastoma, IDH-wildtype is an IDH-wildtype and H3-wildtype diffusely astrocytic glioma. Furthermore, this tumour must display at least one or more of the following: microvascular proliferation, necrosis..."

This case meets these criteria with the presence of both microvascular proliferation and necrosis, along with the IDH-wildtype status. The H3 status is not mentioned in the description, but absence of H3 mutation can be assumed unless otherwise stated, as H3-mutant gliomas are typically specified separately in the classification.

ChatGPT Response:

Based on the provided histopathological description, the diagnosis is most likely **glioblastoma (GBM)**, also known as glioblastoma multiforme. Here is the reasoning:

1. **Infiltrating, hypercellular astrocytic neoplasm**: Indicates a tumor derived from astrocytes and is highly cellular.
2. **Hyperchromatic, elongated nuclei and irregular nuclear membranes**: Features suggestive of malignancy and cellular atypia.
3. **Mitotic activity**: Indicative of a high-grade tumor.
4. **Microvascular endothelial proliferation and pseudopalisading necrosis**: Classic features of glioblastoma.
5. **Negative for IDH1**: Most primary (de novo) glioblastomas are IDH1 negative. IDH1 mutations are more commonly seen in lower-grade gliomas and secondary glioblastomas.
6. **Retained nuclear ATRX expression**: Retention of ATRX is more typical of glioblastoma compared to other astrocytomas which often show loss of ATRX expression.
7. **High Ki67 expression**: Indicates high proliferative activity, consistent with a high-grade malignancy.

Glioblastoma is classified as a Grade IV tumor by the World Health Organization (WHO) due to its aggressive nature and poor prognosis.

ChatGPT RAG response:

Based on the provided histopathological description and the guidelines from the WHO document, the diagnosis is most likely **Glioblastoma, IDH-wildtype (CNS WHO grade 4)**. Here is the reasoning:

1. **Histopathological Features**:
- **Hypercellular astrocytic neoplasm**: This indicates a glioma of astrocytic origin.
- **Hyperchromatic, elongated nuclei and irregular nuclear membranes**: These features are consistent with malignant astrocytic tumors.
- **Mitotic activity**: This is a sign of high-grade malignancy.
- **Microvascular endothelial proliferation**: This is a characteristic feature of glioblastoma.
- **Pseudopalisading necrosis**: This is also a hallmark of glioblastoma.

2. **Molecular Features**:
- **Negative for IDH1 mutation**: This indicates an IDH-wildtype tumor.
- **Retained nuclear ATRX expression**: This further supports the diagnosis of a glioblastoma IDH-wildtype rather than an IDH-mutant astrocytoma.

3. **High Ki67 Expression**:
- This indicates a high proliferative index, which is typical for glioblastoma.

These features align with the criteria for glioblastoma, IDH-wildtype as outlined in the WHO classification:

- "Glioblastoma IDH-wildtype is a diffuse astrocytic glioma that is IDH-wildtype and H3-wildtype and has one or more of the following histological or genetic features: microvascular proliferation, necrosis, TERT promoter mutation, EGFR gene amplification, +7/−10 chromosome copy-number changes (CNS WHO grade 4)" (WHO Classification, Section on Glioblastoma IDH-wildtype).
- "In an IDH- and H3-wildtype diffuse glioma, at least one of these features (i.e., microvascular proliferation or necrosis) is sufficient for the diagnosis of glioblastoma" (WHO Classification, Section on Glioblastoma IDH-wildtype).

Therefore, the diagnosis is ****Glioblastoma, IDH-wildtype (CNS WHO grade 4)****【3†source】.

G2: N19 2728 - GBM IDHwt

Cores of white matter with hypercellular brain tissue. There is an infiltrating fibrillary astrocytic tumour which is mitotically active with small areas of necrosis and palisades. Vascular proliferation is present. The immunoprofile of the tumour is as follows. IDH1 negative and ATRX is expressed in the nuclei. Ki67 index is approximately 25%.

What do you think the diagnosis would be?

GT: glioblastoma, IDH-wildtype

Llama Response:

Based on the histopathological description provided, the diagnosis is most likely:

Glioblastoma, IDH-wildtype (WHO grade 4)

Key features supporting this diagnosis:

1. Infiltrating, hypercellular astrocytic neoplasm: Indicative of a high-grade astrocytoma.
2. Hyperchromatic, elongated nuclei with irregular nuclear membranes: Typical of high-grade gliomas.
3. Mitotic activity: Suggests rapid cell division, common in high-grade tumors.
4. Microvascular endothelial proliferation: A hallmark feature of glioblastoma.
5. Pseudopalisading necrosis: Another characteristic feature of glioblastoma.
6. IDH1 negative: Indicates IDH-wildtype status, which is more common in primary glioblastomas.
7. Retained nuclear ATRX expression: Consistent with IDH-wildtype glioblastoma (ATRX mutations are more common in IDH-mutant astrocytomas).
8. High Ki67 expression: Indicates a high proliferation rate, typical of glioblastomas.

The combination of these features, particularly the presence of microvascular proliferation and necrosis in an astrocytic tumor, is diagnostic of glioblastoma. The IDH-wildtype status and the absence of other typical lower-grade glioma mutations (like ATRX loss) further support this diagnosis.

Llama RAG response:

Based on the histopathological description provided and using only the information from the attached WHO diagnostic guidelines document, the diagnosis appears to be:

Glioblastoma, IDH-wildtype (CNS WHO grade 4)

Here's the rationale for this diagnosis, with references to the document:
1. Histological features:

- The tumor is described as an "infiltrating, hypercellular astrocytic neoplasm," which aligns with the definition of glioblastoma as a "diffusely infiltrating, highly cellular glioma composed of astrocytic,

usually poorly differentiated tumour cells" (Glioblastoma, IDH-wildtype section, Histopathology subsection).
- The presence of "microvascular endothelial proliferation and pseudopalisading necrosis" are characteristic diagnostic features of glioblastoma (same subsection).

2. Molecular features:
- The tumor cells are "negative for IDH1," which is consistent with the IDH-wildtype status required for this diagnosis (Glioblastoma, IDH-wildtype section, Definition).
- "Retained nuclear ATRX expression" is typical in the "vast majority" of IDH-wildtype glioblastomas (Immunophenotype subsection).

3. Proliferation:
- The tumor is described as "mitoticly active" with "high" Ki67 expression, which is consistent with the "readily identifiable" and often "brisk" mitotic activity described for glioblastomas (Histopathology subsection).

4. Grade:
- Glioblastoma, IDH-wildtype is defined as CNS WHO grade 4 (Definition subsection).

The diagnosis of Glioblastoma, IDH-wildtype can be made based on the presence of microvascular proliferation and/or necrosis in an IDH- and H3-wildtype diffuse glioma, as stated in the Essential and desirable diagnostic criteria section: "Glioblastoma, IDH-wildtype is an IDH-wildtype and H3-wildtype diffusely astrocytic glioma. Furthermore, this tumour must display at least one or more of the following: microvascular proliferation, necrosis..."

This case meets these criteria with the presence of both microvascular proliferation and necrosis, along with the IDH-wildtype status. The H3 status is not mentioned in the description, but absence of H3 mutation can be assumed unless otherwise stated, as H3-mutant gliomas are typically specified separately in the classification.

Based on the histopathological description, I would diagnose this tumour as a Glioblastoma (WHO Grade IV).

Here's my reasoning:

- The presence of an infiltrating fibrillary astrocytic tumour with mitotic activity, necrosis, and palisades suggests a high-grade glioma.
- The vascular proliferation further supports this diagnosis.
- The IDH1 negativity and ATRX expression are consistent with a glioblastoma, as IDH1 mutations are more commonly seen in lower-grade gliomas.
- The high Ki67 index of 25% suggests a rapidly proliferating tumour, which is also consistent with a glioblastoma.

Given the presence of necrosis, mitotic activity, and vascular proliferation, I would grade this tumour as a **Glioblastoma, WHO Grade IV**.

Llama RAG Response:

Based on the histopathological description and immunoprofile, I believe the diagnosis is **Glioblastoma, IDH-wildtype, WHO grade 4**.

Here's the reasoning:

- The description of an infiltrating fibrillary astrocytic tumour with mitotic activity, necrosis, and palisades is consistent with a high-grade glioma, such as Glioblastoma (page 2 of the attached document).
- The presence of vascular proliferation further supports this diagnosis (page 2 of the attached document).
- The IDH1 negative immunoprofile indicates that the tumour is IDH-wildtype, which is consistent with Glioblastoma, IDH-wildtype (page 12 of the attached document).
- The ATRX expression in the nuclei is also consistent with Glioblastoma, IDH-wildtype (page 12 of the attached document).
- The Ki67 index of approximately 25% is consistent with a high-grade glioma, such as Glioblastoma (page 12 of the attached document).
- The WHO grade 4 is assigned based on the presence of necrosis, mitotic activity, and vascular proliferation, which are all features of high-grade gliomas (page 14 of the attached document).

Therefore, based on the histopathological description and immunoprofile, I believe the diagnosis is Glioblastoma, IDH-wildtype, WHO grade 4.

Claude Response:

Based on the histopathological description provided, the diagnosis is most likely:

Glioblastoma, WHO grade IV (GBM, IDH-wildtype)

Here's the reasoning behind this diagnosis:

1. Infiltrating fibrillary astrocytic tumor: This indicates an astrocytic origin, which is characteristic of gliomas.
2. Mitotically active: High proliferative activity is typical of high-grade gliomas.
3. Necrosis with palisading: This is a hallmark feature of glioblastoma.
4. Vascular proliferation: Another key feature of glioblastoma.
5. Immunoprofile:
    - IDH1 negative: Suggests IDH-wildtype status, which is more common in primary (de novo) glioblastomas.
    - ATRX expressed: ATRX retention is consistent with IDH-wildtype glioblastoma.
    - Ki67 index of approximately 25%: Indicates a high proliferation rate, typical of high-grade gliomas.
6. The combination of these features, especially the presence of necrosis and vascular proliferation, along with the high Ki67 index, strongly supports a diagnosis of glioblastoma.
7. The WHO grade IV is assigned to glioblastomas due to their aggressive nature and poor prognosis.

The IDH-wildtype status is inferred from the negative IDH1 immunostaining, which is the most common subtype of glioblastoma in adults.

This diagnosis aligns with the 2021 WHO Classification of Tumors of the Central Nervous System, which emphasizes the importance of both histological and molecular features in glioma classification.

Claude RAG Response:
Based on the histopathological description and immunoprofile provided, and using only the information from the attached WHO diagnostic guidelines document, the diagnosis appears to be:

Glioblastoma, IDH-wildtype (CNS WHO grade 4)

Here's the reasoning based on the WHO guidelines:
1. Histological features:
- Infiltrating fibrillary astrocytic tumor
- Mitotically active
- Areas of necrosis with palisades
- Vascular proliferation

These features are consistent with the description of glioblastoma in the document: "Glioblastoma, IDH-wildtype, is typically a diffusely infiltrating, highly cellular glioma composed of astrocytic, usually poorly differentiated tumour cells that show nuclear atypia and often marked pleomorphism. Mitotic activity is readily identifiable in most cases and is often brisk. Microvascular proliferation and necrosis, with or without perinecrotic palisading, are characteristic diagnostic features." (from the "Histopathology" section of Glioblastoma, IDH-wildtype)
2. Immunoprofile:
- IDH1 negative: This supports the IDH-wildtype status.
- ATRX expressed in nuclei: The document states, "Nuclear immunostaining for ATRX is retained in the vast majority of tumours" for IDH-wildtype glioblastomas (from the "Immunophenotype" section).
- Ki67 index of approximately 25%: This high proliferation index is consistent with a high-grade glioma like glioblastoma.

3. Diagnostic criteria:
The essential diagnostic criteria for Glioblastoma, IDH-wildtype include: "Glioblastoma, IDH-wildtype is an IDH-wildtype and H3-wildtype diffusely astrocytic glioma. Furthermore, this tumour must display at least one or more of the following: microvascular proliferation, necrosis, TERT promotor mutation, EGFR gene amplification, +7/-10 chromosome copy-number alterations." (from the "Essential and desirable diagnostic criteria" section)

The provided description meets these criteria with the presence of microvascular proliferation and necrosis.

Given the histological features, immunoprofile, and the fact that it meets the essential diagnostic criteria outlined in the WHO guidelines, the most appropriate diagnosis based solely on the provided information is Glioblastoma, IDH-wildtype (CNS WHO grade 4).

ChatGPT Response:
Based on the histopathological description and molecular findings provided, the tumour is likely to be a **glioblastoma (GBM), IDH-wildtype**. Here’s the rationale:

1. **Histopathological Features**:

- **Moderately pleomorphic glial cells**: Glioblastomas often show pleomorphism.
- **Increased tumour cell density**: This is consistent with glioblastoma.
- **Irregular isochromic nuclei**: Characteristic of malignant gliomas.
- **Fibrillary stroma**: Commonly seen in gliomas.
- **Scattered microcalcifications**: Can be present in glioblastomas.
- **Mitotic activity inconspicuous**: This could be due to the small size of the biopsy, but it doesn't exclude a high-grade glioma.

2. **Molecular Findings**:
- **Negative for mutant IDH1 (R132H)** and **IDH2 mutations**: This suggests the tumour is IDH-wildtype.
- **Negative for mutant H3F3A (K27M)**: This rules out diffuse midline glioma.
- **ATRX retained**: Loss of ATRX is commonly seen in astrocytomas, but its retention does not rule out glioblastoma.
- **EGFR amplification**: This is a common feature of glioblastoma, particularly the IDH-wildtype subtype.

3. **Absence of Microvascular Proliferation and Necrosis**: These features are characteristic of glioblastomas, but their absence in a small biopsy does not exclude the diagnosis. Larger samples often reveal these features.
4. **Negative for IDH mutations**: According to the 2016 and 2021 WHO classifications, glioblastomas can be classified as IDH-wildtype or IDH-mutant, with IDH-wildtype being the more common form and often associated with a worse prognosis. The presence of EGFR amplification further supports the diagnosis of an IDH-wildtype glioblastoma.

Given the combination of histopathological and molecular features, the most likely diagnosis is ****glioblastoma, IDH-wildtype****. The absence of microvascular proliferation and necrosis in the small biopsy specimen does not definitively rule out glioblastoma as these features might be present in other parts of the tumour.

ChatGPT RAG response:

Based on the provided histopathological description and the WHO diagnostic guidelines for CNS tumours, the diagnosis can be determined as follows:

Tumour Type
The presence of moderately pleomorphic glial cells, increased tumour cell density, irregular nuclei, and fibrillary stroma are consistent with a diffuse glioma. The lack of IDH1 and IDH2 mutations, along with the retention of ATRX and absence of H3 K27M mutation, suggest an IDH-wildtype glioma. The presence of EGFR amplification further supports this diagnosis.
Tumour Classification
According to the WHO classification of CNS tumours, the description and molecular findings are indicative of a **glioblastoma, IDH-wildtype**. Glioblastoma, IDH-wildtype, is characterized by the presence of microvascular proliferation, necrosis, or genetic features such as TERT promoter mutation, EGFR gene amplification, or +7/−10 chromosome copy-number changes (CNS WHO grade 4).
Grade
The absence of microvascular proliferation and necrosis typically seen in a small biopsy does not rule out glioblastoma if other criteria, such as EGFR amplification, are present. Therefore, even in the absence of

these histological features, the molecular finding of EGFR amplification is sufficient to classify this as glioblastoma, IDH-wildtype, CNS WHO grade 4.

Conclusion
The diagnosis is **glioblastoma, IDH-wildtype, CNS WHO grade 4**.

This diagnosis is supported by the following references from the WHO Classification of Tumours:

- **Glioblastoma IDH-wildtype** is a diffuse astrocytic glioma that is IDH-wildtype and has one or more of the following histological or genetic features: microvascular proliferation, necrosis, TERT promoter mutation, EGFR gene amplification, +7/−10 chromosome copy-number changes (CNS WHO grade 4)【3†source】.
- The description of the tumour fits the histopathological and molecular profile provided in the WHO guidelines, including the characteristic features of glioblastoma, IDH-wildtype【3†source】.

G3: N18 2124 - A2 IDHwt

Histology shows two small biopsy specimens of brain parenchyma diffusely infiltrated by moderately pleomorphic glial cells. The tumour cell density is mildly increased within the specimens. The tumour cells have irregular isochromic nuclei and indistinct cell boundaries. The stroma is fibrillary and contains scattered microcalcification. Mitotic activity is inconspicuous however, the specimen is small. Neither microvascular proliferation nor necrosis are observed.
The referring pathology report states that the tumour is negative for mutant IDH1 (R132H) and mutant H3F3A (K27M), but ATRX is retained on immunohistochemistry. Sequencing was negative for IDH1 and IDH2 mutations, however an EGFR amplification was noted to be present.
What do you think the diagnosis would be?

GT: glioblastoma, IDH-wildtype

Llama Response:

What a great question!

Based on the histopathological description, I would diagnose this tumor as a ****Glioblastoma, IDH-wildtype, WHO grade IV****.

Here's my reasoning:

- The presence of moderately pleomorphic glial cells with irregular isochromic nuclei and indistinct cell boundaries suggests a high-grade glioma.
- The absence of mutant IDH1 (R132H) and mutant H3F3A (K27M) suggests that this is an IDH-wildtype glioblastoma.
- The retention of ATRX staining is consistent with a glioblastoma, as ATRX is typically retained in these tumors.
- The presence of EGFR amplification is a common feature of glioblastomas, particularly IDH-wildtype glioblastomas.
- The absence of microvascular proliferation and necrosis may be due to the small size of the biopsy specimens, but it's also possible that these features may be present in other areas of the tumor.
- The lack of mitotic activity in the specimen may be due to the small size of the biopsy, but it's also possible that the tumor may have a relatively low proliferative index.

Overall, based on the combination of histological features and molecular markers, I believe the diagnosis is a Glioblastoma, IDH-wildtype, WHO grade IV.

Llama RAG response:

Based on the histopathological description and molecular findings, I would diagnose this tumour as a **Glioblastoma, IDH-wildtype, grade 4**.

Here's how I reached this diagnosis using the attached document:

1. **Morphology**: The description of moderately pleomorphic glial cells with irregular isochromic nuclei and indistinct cell boundaries suggests a high-grade glioma (p. 2, CNS Tumour Grading).
2. **Absence of IDH mutation**: The negative result for IDH1 (R132H) and IDH2 mutations, as well as retained ATRX expression, supports the diagnosis of an IDH-wildtype tumour (p. 3, Gene and Protein Nomenclature).
3. **EGFR amplification**: The presence of EGFR amplification is a characteristic molecular feature of Glioblastoma, IDH-wildtype (p. 5, Combined histological and molecular grading).
4. **Grade 4**: The presence of EGFR amplification, lack of IDH mutation, and the morphological features described suggest a high-grade glioma, which corresponds to a grade 4 tumour in the CNS WHO grading system (p. 4, CNS Tumour Grading).

Based on these findings, I conclude that the diagnosis is Glioblastoma, IDH-wildtype, grade 4.

Please let me know if I'm correct or not!

Claude Response:

Based on the histopathological description and molecular findings provided, the most likely diagnosis is:

Diffuse Astrocytoma, IDH-wildtype, WHO Grade 2

Reasoning:
1. Histology:
   - Diffuse infiltration of brain parenchyma by glial cells
   - Moderate pleomorphism
   - Mildly increased cell density
   - Fibrillary stroma with microcalcifications
   - Absence of significant mitotic activity, microvascular proliferation, and necrosis
2. Molecular findings:
   - IDH1 (R132H) mutation negative by immunohistochemistry
   - IDH1 and IDH2 mutations negative by sequencing
   - H3F3A (K27M) mutation negative
   - ATRX retained
   - EGFR amplification present

The histological features are consistent with a diffuse glioma. The absence of significant mitotic activity, microvascular proliferation, and necrosis suggests a lower-grade tumor (WHO Grade 2) rather than a high-grade glioma.

The lack of IDH mutations (both by immunohistochemistry and sequencing) classifies this as an IDH-wildtype tumor. The presence of EGFR amplification is more commonly associated with IDH-wildtype gliomas.

It's important to note that the 2021 WHO Classification of CNS Tumors introduced new molecular criteria for glioblastoma, IDH-wildtype. However, the information provided does not mention all the required

molecular features (such as TERT promoter mutation, combined gain of chromosome 7 and loss of chromosome 10, or EGFR gene amplification) to definitively classify this as a glioblastoma, IDH-wildtype.

Given the limited specimen size and the absence of high-grade features, the most appropriate diagnosis based on the provided information would be a Diffuse Astrocytoma, IDH-wildtype, WHO Grade 2. However, close clinical follow-up and potentially additional molecular testing might be warranted, as some IDH-wildtype lower-grade gliomas can behave more aggressively than their histological grade suggests.

Claude RAG response:

Based on the histopathological description and molecular findings provided, and using only the information from the attached WHO diagnostic guidelines document, I believe the diagnosis is:

Glioblastoma, IDH-wildtype, CNS WHO grade 4

Here's my reasoning, referencing the attached document:

1. Histology: The description indicates a diffusely infiltrating glial tumor with moderate pleomorphism and mildly increased cellularity. This is consistent with a diffuse astrocytic glioma, as described in the "Histopathology" section of the Glioblastoma, IDH-wildtype entry.
2. IDH status: The tumor is negative for IDH1 R132H immunohistochemistry and sequencing was negative for IDH1 and IDH2 mutations. This confirms the tumor is IDH-wildtype, which is a key feature of Glioblastoma, IDH-wildtype.
3. H3 status: The tumor is negative for H3F3A K27M mutation, ruling out diffuse midline glioma, H3 K27-altered.
4. ATRX status: ATRX is retained on immunohistochemistry, which is typical for most IDH-wildtype glioblastomas, as stated in the "Immunophenotype" section: "Nuclear immunostaining for ATRX is retained in the vast majority of tumours".
5. EGFR amplification: The presence of EGFR amplification is crucial for the diagnosis. According to the "Essential and desirable diagnostic criteria" section for Glioblastoma, IDH-wildtype, one of the criteria is "EGFR gene amplification". This molecular feature allows for the diagnosis of glioblastoma even in the absence of microvascular proliferation or necrosis, which were not observed in this small biopsy specimen.

The "Definition" section for Glioblastoma, IDH-wildtype states: "Glioblastoma, IDH-wildtype, is a diffuse, astrocytic glioma that is IDH-wildtype and H3-wildtype and has one or more of the following histological or genetic features: microvascular proliferation, necrosis, TERT promoter mutation, EGFR gene amplification, +7/−10 chromosome copy-number changes (CNS WHO grade 4)."

While the biopsy doesn't show microvascular proliferation or necrosis, the presence of EGFR amplification in an IDH-wildtype diffuse astrocytic glioma is sufficient for the diagnosis of glioblastoma, as stated in the "Diagnostic molecular pathology" section: "The presence of at least one of these aberrations [including EGFR gene amplification] in an IDH- and H3-wildtype diffuse glioma allows for the diagnosis of IDH-wildtype glioblastoma even in the absence of microvascular proliferation and/or necrosis."

Therefore, based on the histological features of a diffuse astrocytic glioma combined with the molecular profile (IDH-wildtype, H3-wildtype, EGFR amplification), the diagnosis is Glioblastoma, IDH-wildtype, CNS WHO grade 4.

ChatGPT Response:

Based on the histopathological description and molecular findings provided, the tumour is likely to be a **glioblastoma (GBM), IDH-wildtype**. Here's the rationale:

1. **Histopathological Features**:
- **Moderately pleomorphic glial cells**: Glioblastomas often show pleomorphism.
- **Increased tumour cell density**: This is consistent with glioblastoma.
- **Irregular isochromic nuclei**: Characteristic of malignant gliomas.
- **Fibrillary stroma**: Commonly seen in gliomas.
- **Scattered microcalcifications**: Can be present in glioblastomas.
- **Mitotic activity inconspicuous**: This could be due to the small size of the biopsy, but it doesn't exclude a high-grade glioma.

2. **Molecular Findings**:
- **Negative for mutant IDH1 (R132H)** and **IDH2 mutations**: This suggests the tumour is IDH-wildtype.
- **Negative for mutant H3F3A (K27M)**: This rules out diffuse midline glioma.
- **ATRX retained**: Loss of ATRX is commonly seen in astrocytomas, but its retention does not rule out glioblastoma.
- **EGFR amplification**: This is a common feature of glioblastoma, particularly the IDH-wildtype subtype.

3. **Absence of Microvascular Proliferation and Necrosis**: These features are characteristic of glioblastomas, but their absence in a small biopsy does not exclude the diagnosis. Larger samples often reveal these features.
4. **Negative for IDH mutations**: According to the 2016 and 2021 WHO classifications, glioblastomas can be classified as IDH-wildtype or IDH-mutant, with IDH-wildtype being the more common form and often associated with a worse prognosis. The presence of EGFR amplification further supports the diagnosis of an IDH-wildtype glioblastoma.

Given the combination of histopathological and molecular features, the most likely diagnosis is ****glioblastoma, IDH-wildtype****. The absence of microvascular proliferation and necrosis in the small biopsy specimen does not definitively rule out glioblastoma as these features might be present in other parts of the tumour.

ChatGPT RAG response:

Based on the provided histopathological description and the WHO diagnostic guidelines for CNS tumours, the diagnosis can be determined as follows:

Tumour Type
The presence of moderately pleomorphic glial cells, increased tumour cell density, irregular nuclei, and fibrillary stroma are consistent with a diffuse glioma. The lack of IDH1 and IDH2 mutations, along with the retention of ATRX and absence of H3 K27M mutation, suggest an IDH-wildtype glioma. The presence of EGFR amplification further supports this diagnosis.

Tumour Classification
According to the WHO classification of CNS tumours, the description and molecular findings are indicative of a **glioblastoma, IDH-wildtype**. Glioblastoma, IDH-wildtype, is characterized by the presence of microvascular proliferation, necrosis, or genetic features such as TERT promoter mutation, EGFR gene amplification, or +7/−10 chromosome copy-number changes (CNS WHO grade 4).
Grade
The absence of microvascular proliferation and necrosis typically seen in a small biopsy does not rule out glioblastoma if other criteria, such as EGFR amplification, are present. Therefore, even in the absence of these histological features, the molecular finding of EGFR amplification is sufficient to classify this as glioblastoma, IDH-wildtype, CNS WHO grade 4.
Conclusion
The diagnosis is **glioblastoma, IDH-wildtype, CNS WHO grade 4**.

This diagnosis is supported by the following references from the WHO Classification of Tumours:

- **Glioblastoma IDH-wildtype** is a diffuse astrocytic glioma that is IDH-wildtype and has one or more of the following histological or genetic features: microvascular proliferation, necrosis, TERT promoter mutation, EGFR gene amplification, +7/−10 chromosome copy-number changes (CNS WHO grade 4)【3†source】.
- The description of the tumour fits the histopathological and molecular profile provided in the WHO guidelines, including the characteristic features of glioblastoma, IDH-wildtype【3†source】.

G4: N13 1120 - A3 IDHwt

Large resection specimen displaying a moderately cellular glial tumour. Cells have rather pleomorphic nuclei. Many of the neoplastic astrocytes have a gemistocytic appearance and focally, an oligodendroglial pattern emerges. There is a variable, but in places extensive, fibrillary matrix. True vascular endothelial proliferations are not seen but vessels with thickened endothelium and a tendency to the formation of microvascular proliferation are noted in several places. Small areas of cystic degenerative changes are noted in the tumour but there is no bona fide necrosis. The tumour strongly expresses GFAP and patchy MAP2. The ki67 labelling index nears 10%. Immunostaining for mutant IDH1 is negative.

What do you think the diagnosis would be?

GT: glioblastoma, IDH-wildtype

Llama Response:

What a great question!

Based on the histopathological description, I believe the diagnosis is an **Anaplastic Astrocytoma, WHO Grade III.**

Here's why:

- The tumor is described as "moderately cellular" and "pleomorphic nuclei" suggests an anaplastic feature, which is consistent with a high-grade glioma.
- The presence of gemistocytic astrocytes and focal oligodendroglial patterns is also consistent with an anaplastic astrocytoma.
- The variable, but extensive, fibrillary matrix is also characteristic of an astrocytic tumor.
- The absence of true vascular endothelial proliferations, but presence of vessels with thickened endothelium and microvascular proliferation, suggests an anaplastic astrocytoma rather than a glioblastoma (which would typically show more prominent vascular proliferation).
- The cystic degenerative changes, but lack of necrosis, is also consistent with an anaplastic astrocytoma.
- The strong expression of GFAP and patchy MAP2 is consistent with an astrocytic tumor.
- The ki67 labelling index of near 10% suggests a relatively high proliferative activity, consistent with an anaplastic astrocytoma.
- The negative staining for mutant IDH1 is also consistent with an anaplastic astrocytoma, as IDH mutations are more commonly seen in lower-grade gliomas.

Overall, the combination of anaplastic features, astrocytic differentiation, and high proliferative activity leads me to believe that the diagnosis is an Anaplastic Astrocytoma, WHO Grade III.

Llama RAG response:

Based on the histopathological description, I believe the diagnosis would be an **IDH-wildtype glioblastoma, grade 4**.

Here's my reasoning:

- The tumour is described as a moderately cellular glial tumour, which is consistent with a high-grade glioma.
- The presence of pleomorphic nuclei and a variable, but extensive, fibrillary matrix suggests a high-grade glioma.
- The presence of gemistocytic astrocytes and oligodendroglial patterns is also consistent with a high-grade glioma.
- The absence of true vascular endothelial proliferations, but the presence of vessels with thickened endothelium and microvascular proliferation, suggests a high-grade glioma.
- The presence of cystic degenerative changes, but no bona fide necrosis, is also consistent with a high-grade glioma.
- The strong expression of GFAP and patchy MAP2 is consistent with a glial tumour.
- The ki67 labelling index nears 10%, which suggests a high proliferative index, consistent with a high-grade glioma.
- The negative immunostaining for mutant IDH1 suggests that the tumour is IDH-wildtype.

According to the WHO classification of CNS tumours (5th edition), an IDH-wildtype glioblastoma is characterized by a high-grade glial tumour with a high proliferative index, and the absence of IDH1/2 mutations. The presence of a fibrillary matrix, gemistocytic astrocytes, and oligodendroglial patterns are also consistent with this diagnosis.

Reference: WHO Classification of Tumours of the Central Nervous System, 5th edition, page 24-25.

Claude Response:

Based on the histopathological description provided, the diagnosis appears to be:

Diffuse Astrocytoma, WHO grade 3 (Anaplastic Astrocytoma)

Key features supporting this diagnosis:

1. Moderately cellular glial tumor with pleomorphic nuclei
2. Presence of gemistocytic astrocytes and focal oligodendroglial pattern
3. Fibrillary matrix
4. Absence of true vascular endothelial proliferation, but presence of thickened endothelium and tendency towards microvascular proliferation
5. Absence of bona fide necrosis
6. Strong GFAP expression (typical for astrocytic tumors)
7. Ki67 labeling index of nearly 10% (indicating increased proliferative activity)
8. Negative IDH1 mutation (suggesting IDH-wildtype status)

The absence of frank necrosis and definitive endothelial proliferation rules out a grade 4 glioblastoma. However, the elevated Ki67 index, nuclear pleomorphism, and tendency towards microvascular proliferation suggest a higher grade than a typical grade 2 diffuse astrocytoma.

The WHO 2021 classification emphasizes molecular features for glioma classification. The IDH-wildtype status in this case is an important factor, as IDH-wildtype diffuse astrocytic gliomas in adults are often considered to have a behavior closer to glioblastoma. However, without additional molecular information (such as CDKN2A/B homozygous deletion or EGFR amplification), and given the histological features described, a grade 3 designation seems most appropriate based on the information provided.

It's worth noting that additional molecular testing could potentially alter this diagnosis, particularly given the IDH-wildtype status. If you have any additional molecular information or if further testing is planned, it could help refine the diagnosis further.

Claude RAG response:

Based on the histopathological description provided and using only the information from the attached WHO diagnostic guidelines document, I believe the diagnosis is most likely:

Glioblastoma, IDH-wildtype, CNS WHO grade 4

Here's my reasoning, referencing the attached document:

1. The tumor is described as a "moderately cellular glial tumour" with "pleomorphic nuclei" and "neoplastic astrocytes," which aligns with the description of glioblastoma in the document: "Glioblastoma, IDH-wildtype, is typically a diffusely infiltrating, highly cellular glioma composed of astrocytic, usually poorly differentiated tumour cells that show nuclear atypia and often marked pleomorphism."
2. The presence of "vessels with thickened endothelium and a tendency to the formation of microvascular proliferation" is significant. The document states that "microvascular proliferation" is one of the characteristic diagnostic features of glioblastoma.
3. While "true vascular endothelial proliferations are not seen" and there is "no bona fide necrosis," the document mentions that "In an IDH- and H3-wildtype diffuse glioma, at least one of these features (i.e. microvascular proliferation or necrosis) is sufficient for the diagnosis of glioblastoma."
4. The Ki67 labelling index of nearly 10% is consistent with glioblastoma. The document states that for CNS WHO grade 3 tumors, "the Ki-67 proliferation index is usually in the range of 4–10%, but it can overlap with values for CNS WHO grade 2 tumours at one end of the range and CNS WHO grade 4 tumours at the other."
5. The immunostaining for mutant IDH1 is negative, which is consistent with the IDH-wildtype status. The document states: "By definition, IDH-wildtype glioblastomas lack immunostaining for IDH1 p.R132H."
6. The presence of gemistocytic astrocytes and focal oligodendroglial pattern is not inconsistent with glioblastoma. The document mentions that "Gemistocytes may be present in IDH-wildtype glioblastoma" and that "Occasional glioblastomas contain oligodendrocyte-like clear cells with round nuclei that mimic oligodendroglioma."

While some features might suggest a lower grade (absence of "true" vascular endothelial proliferation and necrosis), the combination of microvascular proliferation, high cellularity, nuclear atypia, and IDH-wildtype status in a diffuse astrocytic tumor supports the diagnosis of glioblastoma, IDH-wildtype, CNS WHO grade 4.

However, it's worth noting that additional molecular testing (such as for TERT promoter mutation, EGFR amplification, or +7/-10 chromosome copy-number changes) could further confirm this diagnosis, as these are also sufficient for diagnosing IDH-wildtype glioblastoma according to the document.

ChatGPT Response:

Based on the histopathological description provided, the diagnosis is likely **Diffuse Astrocytoma, WHO Grade II**.

Here's how this conclusion is reached:

1. **Moderately Cellular Glial Tumour**: Indicates a tumour arising from glial cells, which includes astrocytes.
2. **Pleomorphic Nuclei**: This feature is seen in diffuse astrocytomas but without the marked atypia that would suggest a higher-grade glioma.
3. **Gemistocytic Appearance**: The presence of gemistocytic astrocytes (astrocytes with abundant eosinophilic cytoplasm) is noted, which can be seen in diffuse astrocytomas.
4. **Oligodendroglial Pattern**: While there is a focal oligodendroglial pattern, the dominant glial type seems to be astrocytic. Mixed patterns can be seen in diffuse gliomas.
5. **Fibrillary Matrix**: A variable fibrillary matrix supports the diagnosis of a diffuse astrocytoma.
6. **Vascular Endothelial Proliferation**: True microvascular proliferation is not seen, which helps in excluding higher-grade gliomas (e.g., glioblastoma).
7. **Cystic Degenerative Changes without Bona Fide Necrosis**: The absence of necrosis suggests a lower-grade tumour (necrosis is typically a feature of higher-grade gliomas like glioblastoma).
8. **GFAP and MAP2 Expression**: GFAP (Glial Fibrillary Acidic Protein) positivity confirms glial origin. MAP2 patchy expression is consistent with glial tumours.
9. **Ki67 Labelling Index**: Near 10% indicates a moderate proliferative activity, consistent with Grade II.
10. **IDH1 Mutant Negative**: IDH1 mutation is often seen in lower-grade gliomas and some Grade III gliomas. Its absence might suggest an IDH wild-type astrocytoma, which can have a worse prognosis compared to IDH-mutant ones.

Putting these together, the most fitting diagnosis is ****Diffuse Astrocytoma, WHO Grade II****, particularly due to the absence of features that would suggest a higher grade, such as marked vascular proliferation and necrosis, and the moderate proliferative index (Ki67 near 10%).

ChatGPT RAG response:

Based on the provided histopathological description and using the attached WHO diagnostic guidelines, the diagnosis can be narrowed down. Here is the step-by-step analysis according to the document:

1. **Moderately Cellular Glial Tumor**:
   - The tumor is described as glial, indicating it originates from glial cells.
2. **Pleomorphic Nuclei with Gemistocytic Appearance**:
   - Gemistocytic astrocytes suggest a subtype of astrocytoma.
3. **Oligodendroglial Pattern**:

- Presence of oligodendroglial pattern might suggest a mixed glial tumor, but we must consider molecular features for a definitive classification.

4. **Absence of True Vascular Endothelial Proliferations and Necrosis**:
   - Absence of these features generally suggests it is not a high-grade glioblastoma (IDH-wildtype), which would typically show these characteristics.
5. **Immunostaining Results**:
   - **GFAP Positive**: Indicates glial origin.
   - **MAP2 Patchy**: MAP2 is usually seen in neuronal differentiation but can be patchy in glial tumors.
   - **IDH1 Mutant Negative**: Indicates the tumor is IDH-wildtype.
6. **Ki67 Labeling Index**:
   - Nears 10%, indicating a moderate proliferative index, which is more suggestive of an intermediate to high-grade tumor, but not definitive for the highest grade without other high-grade features like necrosis or significant microvascular proliferation.

Diagnosis
Based on the provided information and references from the WHO document:

Likely Diagnosis:
Glioblastoma, IDH-wildtype (CNS WHO grade 4)

Justification:
- **IDH-wildtype Status**: The tumor is IDH1 negative, fitting the classification of IDH-wildtype glioblastoma.
- **Histopathology Features**: Moderate cellularity with pleomorphic nuclei and gemistocytic cells are common in glioblastomas.
- **Microvascular Changes**: While true endothelial proliferations are absent, thickened endothelium and microvascular proliferation tendencies are observed, which are indicative of a glioblastoma.
- **Ki67 Index**: Nearing 10% is high but not uncommon in glioblastomas.
- **Absence of Necrosis**: While necrosis is often present in glioblastomas, its absence doesn't rule out glioblastoma in the presence of other supporting features.

Refer to the provided WHO document sections on glioblastoma, IDH-wildtype, which describe these histological features and immunohistochemical profiles (Section on Glioblastoma IDH-wildtype: Definition and Histopathology).

Reference:

- World Health Organisation Classification of Tumours, Central Nervous System Tumours, 5th Edition,

Section on Glioblastoma IDH-wildtype【3†source】.

G5: N15 692 - GBM IDHwt

Histology shows several foci of solid growth of an astroglial pleomorphic tumour with infiltration into adjacent brain cortex. Mitoses and pseudopalisading necrosis are frequent and foci of vascular proliferation.
The tumour cells and matrix are immunoreactive for GFAP, ATRX (retained) and negative for mutant IDH1. KI67 labels approximately 15% of the tumour cells.

What do you think the diagnosis would be?

GT: Glioblastoma, IDH-wildtype

Llama Response:

Based on the histopathological description, I would diagnose this tumour as a **Glioblastoma, WHO Grade IV**.

The presence of solid growth, infiltration into adjacent brain cortex, frequent mitoses, and pseudopalisading necrosis are all consistent with a high-grade glioma. The frequent foci of vascular proliferation also support this diagnosis. The immunoreactivity for GFAP and retained ATRX expression are also consistent with a glioblastoma.

The lack of mutant IDH1 expression and the high Ki-67 proliferation index (15%) further support the diagnosis of a high-grade glioma, specifically glioblastoma.

Glioblastoma is the most malignant and aggressive type of astrocytoma, and it is typically classified as WHO Grade IV.

Llama RAG response:

Based on the histopathological description, I believe the diagnosis would be **Glioblastoma, IDH-wildtype, WHO grade 4**.

Here's the reasoning:

- The presence of solid growth, mitoses, and pseudopalisading necrosis are all features consistent with a high-grade glioma (WHO grade 4) [1].
- The immunoreactivity for GFAP and ATRX (retained) is consistent with an astroglial tumour [2].
- The negativity for mutant IDH1 suggests that the tumour is IDH-wildtype [3].
- The KI67 labelling of approximately 15% of the tumour cells indicates a high proliferative activity, which is consistent with a high-grade glioma [4].
- The combination of these features, particularly the presence of pseudopalisading necrosis and frequent mitoses, points towards a diagnosis of Glioblastoma, IDH-wildtype, WHO grade 4 [5].

References:

[1] Page 5, "Grading within Types" section: "In the 2016 WHO classification of CNS tumours, solitary fibrous tumour / haemangiopericytoma was graded in the latter manner, using a single name but with the option of three grades."
[2] Page 3, "Gene and Protein Nomenclature" section: "Gene symbols are presented in italics, but proteins and gene groups (e.g. the family of IDH genes) are not italicized."
[3] Page 5, "Grading within Types" section: "In this context, for example, IDH-mutant astrocytomas extend from grade 2 to grade 4..."
[4] Page 7, "Clinicopathological grading" section: "CNS tumour grading has for decades been linked to overall expected clinical–biological behaviours..."
[5] Page 11, "Combined histological and molecular grading" section: "Examples in the fifth edition include CDKN2A and/or CDKN2B homozygous deletion in IDH-mutant astrocytomas, as well as TERT promoter mutation, EGFR amplification, and +7/−10 copy-number changes in IDH-wildtype glioblastoma."

Note: The references provided are based on the attached document "World Health Organisation Classification of Tumours.docx".

Claude Response:

Based on the histopathological description and immunohistochemistry results provided, the diagnosis is most likely:

Glioblastoma, IDH-wildtype, WHO grade 4

Reasoning:
1. Histological features:
   - Astroglial pleomorphic tumor
   - Infiltrative growth into adjacent brain cortex
   - Frequent mitoses
   - Pseudopalisading necrosis
   - Vascular proliferation
2. Immunohistochemistry:
   - GFAP positive (confirming glial origin)
   - ATRX retained (not mutated)
   - IDH1 mutation negative (wildtype)
   - High Ki-67 labeling index (15%, indicating high proliferative activity)

The combination of infiltrative growth, high-grade features (frequent mitoses, pseudopalisading necrosis, and vascular proliferation), and the immunohistochemical profile (especially IDH-wildtype status) are characteristic of glioblastoma, WHO grade 4.
The absence of IDH1 mutation and the presence of other typical histological features classify this as an IDH-wildtype glioblastoma, which is the most common and aggressive form of glioblastoma in adults.

Would you like me to elaborate on any aspect of this diagnosis or explain the significance of any particular features?

Claude RAG response:

Based on the histopathological description provided and using only the information from the attached WHO diagnostic guidelines document, the diagnosis appears to be:

Glioblastoma, IDH-wildtype, CNS WHO grade 4

Here's the reasoning based on the document:

1. Histological features:
   - The description mentions "astroglial pleomorphic tumour with infiltration into adjacent brain cortex," which aligns with the definition of a diffusely infiltrating astrocytic glioma (as described in the Glioblastoma, IDH-wildtype section).
   - "Mitoses and pseudopalisading necrosis are frequent" - These are characteristic diagnostic features of glioblastoma, as mentioned in the document: "Microvascular proliferation and necrosis, with or without perinecrotic palisading, are characteristic diagnostic features."
   - "Foci of vascular proliferation" - This corresponds to microvascular proliferation, another key feature of glioblastoma.
2. Immunohistochemistry:
   - "Immunoreactive for GFAP" - This is consistent with glioblastoma, as the document states: "Glioblastomas often express GFAP, but the degree of reactivity differs markedly between cases."
   - "ATRX (retained)" - The document mentions: "Nuclear immunostaining for ATRX is retained in the vast majority of tumours."
   - "Negative for mutant IDH1" - This is crucial for the IDH-wildtype diagnosis. The document states: "By definition, IDH-wildtype glioblastomas lack immunostaining for IDH1 p.R132H."
   - "KI67 labels approximately 15% of the tumour cells" - While not specific, this high proliferation index is consistent with a high-grade glioma.
3. Diagnosis:
   - The document states in the "Essential and desirable diagnostic criteria" section for Glioblastoma, IDH-wildtype:
   - "Glioblastoma, IDH-wildtype is an IDH-wildtype and H3-wildtype diffusely astrocytic glioma. Furthermore, this tumour must display at least one or more of the following: microvascular proliferation, necrosis, TERT promotor mutation, EGFR gene amplification, +7/-10 chromosome copy-number alterations."
   - The histological description provided meets these criteria with the presence of microvascular proliferation and necrosis.
4. Grading:
   - Glioblastoma is always assigned CNS WHO grade 4, as it is the highest grade for astrocytic gliomas.

In conclusion, based solely on the information provided in the WHO diagnostic guidelines document, the histological and immunohistochemical features described are most consistent with a diagnosis of Glioblastoma, IDH-wildtype, CNS WHO grade 4.

ChatGPT Response:

Based on the histopathological description provided, the tumour appears to be a Glioblastoma (also known as Glioblastoma Multiforme, GBM). This is suggested by several key features:

1. **Solid Growth of an Astroglial Pleomorphic Tumour**: Indicates a high-grade astrocytic tumour.
2. **Infiltration into Adjacent Brain Cortex**: Characteristic of malignant gliomas, particularly GBM.
3. **Frequent Mitoses and Pseudopalisading Necrosis**: These are hallmark features of GBM.
4. **Vascular Proliferation**: Another key feature of GBM.
5. **Immunoreactivity**:
   - **GFAP**: Indicates glial origin.
   - **ATRX (retained)**: Often retained in primary (de novo) GBM.
   - **Negative for mutant IDH1**: Suggests a primary GBM as IDH mutations are more common in secondary GBM.
6. **KI67 Index (15%)**: Indicates a high proliferative rate consistent with high-grade tumours like GBM.

Diagnosis: **Glioblastoma (WHO Grade IV)**

ChatGPT RAG response:

Based on the provided histopathological description and the WHO diagnostic guidelines for neuropathological tumors, the most likely diagnosis is **Glioblastoma, IDH-wildtype**. Here is the detailed reasoning:

1. **Histological Features**:
   - **Solid Growth of Astroglial Pleomorphic Tumour**: This suggests a high-grade glioma.
   - **Infiltration into Adjacent Brain Cortex**: Indicates an aggressive and invasive tumor.
   - **Frequent Mitoses and Pseudopalisading Necrosis**: These are hallmark features of glioblastoma.
   - **Vascular Proliferation**: Another characteristic feature of glioblastoma.
2. **Immunohistochemical Findings**:
   - **GFAP Positive**: Confirms glial origin.
   - **ATRX Retained**: This indicates the absence of ATRX mutation, which is more common in IDH-wildtype glioblastomas.
   - **IDH1 Negative (wildtype)**: Indicates that the tumor is not an IDH-mutant astrocytoma.
3. **Proliferation Index**:
   - **KI67 Approximately 15%**: This high proliferation index supports the diagnosis of a high-grade tumor such as glioblastoma.
4. **WHO Classification Criteria**:
   - According to the WHO guidelines, glioblastoma IDH-wildtype is defined as a diffuse astrocytic glioma that is IDH-wildtype and H3-wildtype with one or more of the following features: microvascular proliferation, necrosis, TERT promoter mutation, EGFR gene amplification, or +7/−10 chromosome copy-number changes (CNS WHO grade 4)【3†source】.

Given the presence of microvascular proliferation, pseudopalisading necrosis, and negative IDH1 status, the tumor fits the criteria for **Glioblastoma, IDH-wildtype (CNS WHO grade 4)**【3†source】.

G6: NH14 194 - GBM IDHwt

Histology shows a dense astroglial tumour diffusely infiltrating the neuroparenchyma. The tumour is organised as compact fascicles with clusters of epithelioid forms. The tumour cells are pleomorphic, with irregular and hyperchromatic nuclei. Variable amounts of cytoplasm are noted, with occasional cytoplasmic clearing. Tumoural stroma is dense and fibrillar. Frequent mitoses and occasional apoptotic bodies are seen in the tumour. Focal areas of microvascular proliferation and non-pseudopalisading necrosis are present.

GFAP and MAP2 staining reveals intense labelling throughout the tumour, with MAP2 emphasising perinuclear ring-like accentuation in a subset of cells. IDH1 (R132H) immunostaining is negative. Neuronal elements are represented by scattered NeuN-positive entrapped neurons and synaptophysin labelling, which highlights both the neuropil and some tumour cells with focal dot-like expression."

What do you think the diagnosis would be?

GT: Glioblastoma, IDH-wildtype

Llama Response:

Based on the histopathological description, I would diagnose this tumour as a **Glioblastoma, IDH-wildtype, Grade IV**.

Here's my reasoning:
* The dense astroglial tumour with pleomorphic nuclei, frequent mitoses, and areas of microvascular proliferation and necrosis are all consistent with a high-grade glioma.
* The presence of GFAP and MAP2 staining throughout the tumour, including perinuclear ring-like accentuation in some cells, supports an astrocytic origin.
* The absence of IDH1 (R132H) immunostaining suggests that this is an IDH-wildtype tumour, which is more common in Glioblastomas.
* The presence of scattered entrapped neurons and synaptophysin labelling, including focal dot-like expression in some tumour cells, indicates that the tumour is infiltrating the neuroparenchyma.
* The overall morphology and immunophenotype are consistent with a Glioblastoma, which is the most aggressive and malignant type of astrocytoma, corresponding to WHO Grade IV.

What do you think?

Llama RAG response:

Based on the histopathological description provided, I think the diagnosis would be **Glioblastoma, IDH-wildtype, grade 4.**

Here's my reasoning:

* The tumour's compact fascicular organization, pleomorphic nuclei, and frequent mitoses suggest a high-grade tumour (WHO grade 4).
* The presence of microvascular proliferation and non-pseudopalisading necrosis are characteristic features of Glioblastoma (WHO Classification of Tumours, page 14).
* The intense labelling of GFAP and MAP2 supports the diagnosis of a glial tumour, and the perinuclear ring-like accentuation with MAP2 is consistent with astrocytic differentiation.
* The negativity for IDH1 (R132H) immunostaining rules out an IDH-mutant glioblastoma, making IDH-wildtype glioblastoma a more likely diagnosis (WHO Classification of Tumours, page 14).
* The presence of scattered NeuN-positive entrapped neurons and synaptophysin labelling in the tumour cells with focal dot-like expression suggests that the tumour is infiltrating the neuroparenchyma, which is consistent with a Glioblastoma.

References:

* WHO Classification of Tumours, page 14 (Glioblastoma, IDH-wildtype)
* WHO Classification of Tumours, page 16 (IDH-mutant and IDH-wildtype glioblastoma)

Please let me know if my diagnosis is correct or if I need further clarification!

Claude Response

Based on the histopathological description provided, the diagnosis is most likely:

Glioblastoma, IDH-wildtype, WHO grade 4

Key features supporting this diagnosis:
1. Dense astroglial tumor diffusely infiltrating the neuroparenchyma
2. Pleomorphic cells with irregular and hyperchromatic nuclei
3. Frequent mitoses and occasional apoptotic bodies
4. Microvascular proliferation
5. Necrosis (non-pseudopalisading in this case)
6. GFAP positivity, indicating glial origin
7. IDH1 (R132H) immunostaining is negative, suggesting IDH-wildtype status
8. High-grade features overall

The WHO grade 4 designation is based on the presence of microvascular proliferation and necrosis, which are hallmark features of glioblastoma.

Additional notes:
- The epithelioid forms and cytoplasmic clearing might suggest an epithelioid glioblastoma variant, but more information would be needed to confirm this.
- The MAP2 staining with perinuclear ring-like accentuation is interesting and can be seen in some high-grade gliomas.
- The synaptophysin labeling in some tumor cells might indicate some neuronal differentiation, but this can occur in glioblastomas and doesn't change the primary diagnosis.

Would you like me to elaborate on any aspect of this diagnosis or the histological features?

Claude RAG response:

Based on the histopathological description provided and using only the information from the attached WHO diagnostic guidelines document, the most likely diagnosis is:

Glioblastoma, IDH-wildtype (CNS WHO grade 4)

Here's the reasoning for this diagnosis, with references to the attached document:
1. Diffuse infiltration: The description states "a dense astroglial tumour diffusely infiltrating the neuroparenchyma." This is consistent with the definition of glioblastoma as "a diffuse, astrocytic glioma" (Glioblastoma, IDH-wildtype section).
2. High-grade features: The presence of "Frequent mitoses," "microvascular proliferation," and "necrosis" are key diagnostic features of glioblastoma. The document states, "Microvascular proliferation and necrosis, with or without perinecrotic palisading, are characteristic diagnostic features" (Histopathology subsection under Glioblastoma, IDH-wildtype).
3. Cellular morphology: The description of "pleomorphic" cells with "irregular and hyperchromatic nuclei" is consistent with glioblastoma. The document mentions, "Poorly differentiated, fusiform, round, or pleomorphic cells may prevail" (Cellular heterogeneity and glioblastoma patterns subsection).
4. GFAP positivity: The "intense labelling" for GFAP is consistent with glioblastoma. The document states, "Glioblastomas often express GFAP" (Immunophenotype subsection).
5. IDH-wildtype status: The negative IDH1 (R132H) immunostaining suggests an IDH-wildtype tumor. The document specifies, "IDH-wildtype glioblastomas lack immunostaining for IDH1 p.R132H" (Diagnostic molecular pathology subsection).
6. Epithelioid features: The description mentions "clusters of epithelioid forms," which could suggest epithelioid glioblastoma, a histological subtype of glioblastoma (Epithelioid glioblastoma subsection).

While the description provides strong evidence for a glioblastoma diagnosis, it's important to note that additional molecular testing might be beneficial to confirm the IDH-wildtype status, especially if the patient is under 55 years old. The document states, "In patients aged < 55 years, or in patients with a history of lower-grade glioma and/or whose tumours show immunohistochemical loss of nuclear ATRX expression, negative IDH1 p.R132H immunostaining should be followed by DNA sequencing for less common IDH1 or IDH2 mutations" (Diagnostic molecular pathology subsection).

ChatGPT Response:

Based on the histopathological description provided, the diagnosis appears to be **glioblastoma (GBM)**, specifically **IDH-wildtype glioblastoma**. Here's a breakdown of the key features leading to this diagnosis:

1. **Astroglial Tumour:** The tumour is described as an astroglial tumour, which indicates its origin from astrocytes, a type of glial cell.

2. **Diffuse Infiltration:** Glioblastomas are known for their diffuse infiltration into the surrounding neuroparenchyma.
3. **Cellular Features:** The presence of pleomorphic cells with irregular, hyperchromatic nuclei, variable amounts of cytoplasm, and occasional cytoplasmic clearing are indicative of high-grade astrocytomas.
4. **Mitotic Activity and Necrosis:** Frequent mitoses and occasional apoptotic bodies, along with focal areas of microvascular proliferation and necrosis, are hallmark features of glioblastoma.
5. **Staining Patterns:**
- **GFAP and MAP2:** Intense labelling with GFAP (Glial Fibrillary Acidic Protein) and MAP2 (Microtubule-Associated Protein 2) is consistent with a glial origin.
- **IDH1 (R132H) Negative:** The lack of IDH1 mutation (IDH1 wildtype) is significant as it distinguishes IDH-wildtype glioblastomas from IDH-mutant gliomas, which generally have a better prognosis.
- **NeuN and Synaptophysin:** The presence of scattered NeuN-positive neurons and synaptophysin labelling indicates entrapped neurons within the tumour and some neuronal differentiation, which can be seen in glioblastomas.
6. **Microvascular Proliferation and Necrosis:** These features are crucial for diagnosing glioblastoma, as they signify a high-grade tumour (Grade IV according to the World Health Organization (WHO) classification of central nervous system tumours).

Given the combination of diffuse infiltration, high mitotic index, microvascular proliferation, necrosis, and negative IDH1 (R132H) staining, the most likely diagnosis is **glioblastoma (GBM), IDH-wildtype**, which is a WHO Grade IV tumour.

ChatGPT RAG response:

Based on the histopathological description and using the provided WHO classification document, the diagnosis appears to be **Glioblastoma, IDH-wildtype (CNS WHO grade 4)**.

Here's the rationale for this diagnosis:
1. **Histological Features**:
- **Dense astroglial tumour**: Indicates astrocytic origin.
- **Diffuse infiltration of neuroparenchyma**: Characteristic of diffuse gliomas.
- **Pleomorphic cells with irregular and hyperchromatic nuclei**: Suggestive of a high-grade tumour.
- **Frequent mitoses and occasional apoptotic bodies**: Indicative of high proliferative activity.
- **Microvascular proliferation and necrosis**: Key features of glioblastoma.
2. **Immunohistochemical Staining**:
- **Negative IDH1 (R132H) immunostaining**: Indicates IDH-wildtype status.
- **Intense GFAP and MAP2 staining**: Consistent with glial origin.
- **Scattered NeuN-positive neurons and synaptophysin labelling**: Suggestive of entrapped neuronal elements, common in glioblastomas due to their infiltrative nature.
3. **Specific Features Matching Glioblastoma, IDH-wildtype**:
- **Microvascular proliferation and necrosis** are defining features of glioblastoma (refer to the section on Glioblastoma IDH-wildtype in the WHO classification).
- The tumour's negative IDH1 (R132H) status fits the classification of Glioblastoma, IDH-wildtype.
4. **Reference to WHO Document**:
- The WHO document specifies that Glioblastoma, IDH-wildtype is a diffuse astrocytic glioma that is IDH-wildtype and has one or more of the following features: microvascular proliferation, necrosis, TERT

promoter mutation, EGFR gene amplification, or +7/−10 chromosome copy-number changes【3†source】.

Therefore, given the described features and immunohistochemical profile, the most appropriate diagnosis is ****Glioblastoma, IDH-wildtype (CNS WHO grade 4**)**【3†source】.

G7: NH15 2691 - GBM IDHwt

This is a resection specimen including CUSA. The specimen shows a diffusely infiltrative hypercellular pleomorphic astroglial composed of cells with hyperchromatic and angulated nuclei in a fibrillary matrix. In areas there are prominent perivascular arrangement of cells with dense processes centered on the blood vessels. Mitoses including abnormal mitotic figures are frequently seen. Sporadic multinucleate and large bizarre cells are present. There is microvascular endothelial proliferation and tumour necrosis of both pseudopalisading and geographic types.

Immunohistochemistry demonstrates that tumour cells are extensively positive for GFAP, and do not express mutant IDH1 (R132H). The ATRX stain is technically unsatisfactory and a repeat has been requested. H3K27 is negative. Ki-67 proliferation index is deemed high, at more than 20%.

What do you think the diagnosis would be?

GT: Glioblastoma, IDH-wildtype

Llama Response:

What a great question!

Based on the histopathological description, I would suspect that the tumor is a ****Glioblastoma, IDH-wildtype, grade IV****.

Here's why:
* The description of a "diffusely infiltrative hypercellular pleomorphic astroglial" tumor with "hyperchromatic and angulated nuclei" and "fibrillary matrix" suggests a high-grade glioma.
* The presence of "prominent perivascular arrangement of cells" and "microvascular endothelial proliferation" are features commonly seen in glioblastomas.
* The high mitotic activity, including abnormal mitotic figures, and the presence of multinucleate and large bizarre cells support a high-grade diagnosis.
* The extensive positivity for GFAP, a marker for astrocytic tumors, further supports a glioma diagnosis.
* The absence of mutant IDH1 (R132H) expression suggests that the tumor is IDH-wildtype, which is a characteristic feature of glioblastomas.
* The high Ki-67 proliferation index (>20%) is also consistent with a high-grade glioma.
* The presence of tumor necrosis, including pseudopalisading and geographic types, is a hallmark feature of glioblastomas.

Given these features, I believe the diagnosis is likely a Glioblastoma, IDH-wildtype, grade IV.

Llama RAG Response:

Based on the histopathological description and immunohistochemical results, I think the diagnosis would be **Glioblastoma, IDH-wildtype, WHO Grade 4**.

Here's my reasoning:
* The diffusely infiltrative and hypercellular astroglial tumor with pleomorphic cells, frequent mitoses, and abnormal mitotic figures suggest a high-grade glioma (p. 3, CNS Tumour Grading).
* The presence of microvascular endothelial proliferation and tumor necrosis, including pseudopalisading and geographic types, are characteristic features of glioblastoma (p. 3, CNS Tumour Grading).
* The immunohistochemical results show that the tumor cells are positive for GFAP, which is consistent with an astrocytic tumor (p. 5, Introduction to CNS Tumours).
* The lack of expression of mutant IDH1 (R132H) suggests that the tumor is IDH-wildtype (p. 5, CNS Tumour Nomenclature).
* The high Ki-67 proliferation index (>20%) is consistent with a high-grade glioma (p. 3, CNS Tumour Grading).
* The diagnosis of glioblastoma, IDH-wildtype, WHO Grade 4, is supported by the combining histological and molecular information (p. 7, Integrated and Layered Diagnoses).

References:
* Page 3, CNS Tumour Grading
* Page 5, Introduction to CNS Tumours
* Page 5, CNS Tumour Nomenclature
* Page 7, Integrated and Layered Diagnoses

Claude Response:

Based on the histopathological description and immunohistochemistry results provided, the diagnosis is most likely:

Glioblastoma, WHO grade 4 (IDH-wildtype)

Here's the reasoning behind this diagnosis:
1. Histological features:
   - Diffusely infiltrative, hypercellular, pleomorphic astroglial tumor
   - Hyperchromatic and angulated nuclei
   - Prominent perivascular arrangement of cells
   - Frequent mitoses, including abnormal forms
   - Presence of multinucleate and large bizarre cells
   - Microvascular endothelial proliferation
   - Tumor necrosis (both pseudopalisading and geographic types)
2. Immunohistochemistry:
   - GFAP positive (confirming glial origin)
   - IDH1 (R132H) negative (suggesting IDH-wildtype)
   - H3K27 negative (ruling out diffuse midline glioma)
   - High Ki-67 proliferation index (>20%)

The combination of infiltrative growth pattern, high cellularity, nuclear atypia, frequent mitoses, microvascular proliferation, and necrosis are diagnostic features of glioblastoma. The absence of IDH1

R132H mutation (the most common IDH mutation) suggests this is likely an IDH-wildtype glioblastoma, which is more common in primary (de novo) glioblastomas.

The WHO grade 4 is assigned based on the presence of microvascular proliferation and/or necrosis, both of which are present in this case.

It's worth noting that the ATRX stain was unsatisfactory, and the result of the repeat test could provide additional information. However, given the other features, this is unlikely to change the overall diagnosis of glioblastoma.

Claude RAG:

Based on the histopathological description and immunohistochemistry results provided, and using only the information from the attached WHO diagnostic guidelines document, the diagnosis appears to be:

Glioblastoma, IDH-wildtype, CNS WHO grade 4

Here's the rationale for this diagnosis, referencing the relevant sections of the document:
1. Diffusely infiltrative pattern: This is characteristic of glioblastoma, as described in the "Histopathology" section for glioblastoma, which states: "Glioblastoma, IDH-wildtype, is typically a diffusely infiltrating, highly cellular glioma composed of astrocytic, usually poorly differentiated tumour cells that show nuclear atypia and often marked pleomorphism."
2. Microvascular proliferation and necrosis: The description mentions "microvascular endothelial proliferation and tumour necrosis of both pseudopalisading and geographic types." These are key diagnostic features of glioblastoma, as stated in the document: "Microvascular proliferation and necrosis, with or without perinecrotic palisading, are characteristic diagnostic features."
3. High mitotic activity: The description notes "Mitoses including abnormal mitotic figures are frequently seen," which aligns with the document's statement: "Mitotic activity is readily identifiable in most cases and is often brisk."
4. Immunohistochemistry:
   - GFAP positive: This supports the glial nature of the tumor.
   - IDH1 (R132H) negative: This suggests an IDH-wildtype status.
   - H3K27 negative: This helps exclude diffuse midline glioma, H3 K27–altered.
   - High Ki-67 index (>20%): This indicates a high proliferation rate, consistent with a high-grade glioma.
5. IDH-wildtype status: The document states, "Absence of immunoreactivity for IDH1 p.R132H is sufficient (i.e. without further sequencing) to diagnose IDH-wildtype glioblastoma in a patient aged ≥ 55 years at diagnosis who has a histologically classic glioblastoma not located in midline structures and no history of a pre-existing lower-grade glioma."

While the patient's age is not provided, the histological features strongly support a diagnosis of glioblastoma. The IDH-wildtype status is supported by the negative IDH1 (R132H) immunostaining.

The CNS WHO grade 4 designation is based on the presence of microvascular proliferation and necrosis, as outlined in the "Essential and desirable diagnostic criteria" section for glioblastoma, IDH-wildtype.

It's worth noting that additional molecular testing (such as for TERT promoter mutation, EGFR amplification, or +7/-10 chromosome copy-number alterations) could further confirm the diagnosis, but the histological and immunohistochemical features provided are sufficient for the diagnosis of Glioblastoma, IDH-wildtype, CNS WHO grade 4.

ChatGPT Response:

Based on the histopathological description provided, the diagnosis appears to be **glioblastoma (GBM), IDH-wildtype**. Here's the reasoning:

1. **Histological Features**:
   - **Hypercellular pleomorphic astroglial**: Indicates a glial origin with marked cellular diversity.
   - **Hyperchromatic and angulated nuclei in a fibrillary matrix**: Typical of high-grade gliomas.
   - **Perivascular arrangement of cells with dense processes centered on the blood vessels**: A characteristic feature of glioblastoma.
   - **Frequent mitoses, including abnormal mitotic figures**: Suggests a high proliferative activity.
   - **Sporadic multinucleate and large bizarre cells**: Seen in high-grade tumors.
   - **Microvascular endothelial proliferation and tumor necrosis (pseudopalisading and geographic types)**: Key features of glioblastoma.
2. **Immunohistochemistry**:
   - **GFAP positive**: Confirms the glial nature of the tumor.
   - **Mutant IDH1 (R132H) negative**: Suggests IDH-wildtype, which is commonly associated with primary glioblastoma.
   - **ATRX stain technically unsatisfactory**: While ATRX mutation status could be relevant, the current staining is inconclusive.
   - **H3K27 negative**: This excludes certain other gliomas like diffuse midline glioma, H3 K27M-mutant.
   - **Ki-67 proliferation index high (>20%)**: Indicates high proliferative potential, typical of high-grade tumors.

Given these findings, the most likely diagnosis is ****glioblastoma (GBM), IDH-wildtype****, which is classified as a **grade IV** tumor by the World Health Organization (WHO).

ChatGPT RAG response:

Based on the provided histopathological description and using the WHO guidelines, the diagnosis is most likely **Glioblastoma, IDH-wildtype** (CNS WHO grade 4). Here is the reasoning:

Histopathological Features
1. **Diffuse infiltrative hypercellular pleomorphic astroglial tumor**:
   - This indicates a high-grade astrocytic tumor.
2. **Hyperchromatic and angulated nuclei in a fibrillary matrix**:
   - These are typical features of malignant astrocytic tumors.
3. **Prominent perivascular arrangement of cells**:
   - This is often seen in high-grade gliomas, including glioblastomas.

4. **Frequent mitoses including abnormal mitotic figures**:
   - High mitotic activity is characteristic of high-grade tumors.
5. **Sporadic multinucleate and large bizarre cells**:
   - These are often seen in glioblastomas.
6. **Microvascular endothelial proliferation and tumor necrosis of both pseudopalisading and geographic types**:
   - These are hallmark features of glioblastoma (CNS WHO grade 4).

Immunohistochemistry
1. **GFAP positive**:
   - Confirms glial origin.
2. **IDH1 (R132H) negative**:
   - Indicates IDH-wildtype status, which is common in glioblastomas and associated with poorer prognosis compared to IDH-mutant gliomas.
3. **ATRX stain unsatisfactory**:
   - While ATRX loss is common in IDH-mutant astrocytomas, its status here is indeterminate, but given the other features and IDH-wildtype status, the lack of ATRX data does not preclude a glioblastoma diagnosis.
4. **H3K27 negative**:
   - Rules out H3 K27M mutation, which is associated with diffuse midline gliomas.
5. **Ki-67 proliferation index > 20%**:
   - Indicates high proliferative activity, supporting a high-grade diagnosis.

Conclusion
The described features and immunohistochemical profile strongly support the diagnosis of **Glioblastoma, IDH-wildtype (CNS WHO grade 4)**, characterized by high-grade histological features, lack of IDH mutation, and significant proliferative activity.

References:
- WHO Classification of Tumours of the Central Nervous System, 5th Edition【3†source】.

This diagnosis matches the criteria for glioblastoma as outlined in the WHO guidelines, including the presence of microvascular proliferation, necrosis, and the absence of IDH1 mutation.

G8: N16 698 - GBM IDHwt

This is a surgical resection and CUSA specimen containing an infiltrative tumour. The tumour cells display angular and hyperchromatic nuclei arranged within a fibrillary matrix. In some areas, the tumour nuclei are rounded. Mitotic figures are frequently observed. Multiple zones of microvascular endothelial proliferation are discernible. Sporadic regions of necrosis with pseudopalisading features are identified. There is no evidence of sarcomatous transformation.

Immunostaining shows tumour cells are negative for IDH1 mutant (R132H). ATRX is retained within the tumour cell nuclei. The Ki-67 index demonstrates a considerable proliferative activity, approximately 15 to 20%."

What do you think the diagnosis would be?

GT: Glioblastoma, IDH-wildtype

Llama Response:

Based on the histopathological description, I would suggest that the tumor is a **Glioblastoma, WHO Grade IV**.

Here's why:

* The infiltrative growth pattern, angular and hyperchromatic nuclei, and frequent mitotic figures are all consistent with a high-grade glioma.
* The presence of microvascular endothelial proliferation and necrosis with pseudopalisading features are characteristic of glioblastoma.
* The lack of sarcomatous transformation suggests that the tumor is a primary brain tumor rather than a metastasis.
* The negative IDH1 mutant (R132H) immunostain suggests that the tumor is not an IDH-mutant glioma, which is more commonly seen in lower-grade gliomas.
* The retained ATRX expression is also consistent with a high-grade glioma.
* The high Ki-67 index (15-20%) indicates a high proliferative activity, which is typical of glioblastoma.

Overall, the combination of morphological and immunohistochemical features suggests a diagnosis of Glioblastoma, WHO Grade IV.

Llama RAG response

Based on the provided histopathological description and immunostaining results, I would diagnose this tumour as **Glioblastoma, IDH-wildtype, WHO grade 4**.

Here's my reasoning:

* The tumour cells display angular and hyperchromatic nuclei, which is consistent with a high-grade glioma.
* The presence of frequent mitotic figures, microvascular endothelial proliferation, and necrosis with pseudopalisading features support a diagnosis of glioblastoma.
* The immunostaining results show that the tumour cells are negative for IDH1 mutant (R132H), which suggests that the tumour is IDH-wildtype.
* The retention of ATRX within the tumour cell nuclei does not contradict this diagnosis.
* The high Ki-67 index (15-20%) indicates a high proliferative activity, which is consistent with a high-grade glioma.

According to the attached document, specifically the section on "CNS Tumour Grading", glioblastoma is graded as WHO grade 4 (page 5). The document also mentions that IDH-wildtype glioblastomas are a distinct entity (page 11).

Therefore, based on the provided information, I conclude that the diagnosis is Glioblastoma, IDH-wildtype, WHO grade 4.

Claude Response:

Based on the histopathological description provided, the diagnosis is most likely:

Glioblastoma, IDH-wildtype, WHO grade 4

Reasoning:

1. Infiltrative nature: The description mentions an infiltrative tumor, which is characteristic of diffuse gliomas.
2. Cellular features: Angular and hyperchromatic nuclei within a fibrillary matrix are typical of astrocytic tumors.
3. High-grade features:
   - Frequent mitotic figures
   - Microvascular endothelial proliferation
   - Necrosis with pseudopalisading
4. Immunohistochemistry:
   - IDH1 mutant (R132H) negative: This suggests an IDH-wildtype tumor
   - ATRX retained: This is consistent with IDH-wildtype glioblastoma
   - High Ki-67 index (15-20%): Indicates considerable proliferative activity
5. Absence of sarcomatous transformation: Rules out gliosarcoma variant

The presence of microvascular proliferation and/or necrosis, along with the other high-grade features, supports a diagnosis of glioblastoma. The IDH-wildtype status is consistent with the most common form of glioblastoma in adults.

The WHO grade 4 designation is based on the presence of microvascular proliferation and/or necrosis in an astrocytic tumor, which are defining features of glioblastoma according to the current WHO classification of CNS tumors.

Claude RAG response

Based on the histopathological description provided and using only the information from the attached WHO diagnostic guidelines document, the diagnosis that best fits this tumor is:

Glioblastoma, IDH-wildtype (CNS WHO grade 4)

Here's the rationale for this diagnosis, referencing the relevant sections of the document:

1. Infiltrative nature: The description mentions an "infiltrative tumour," which is consistent with the definition of glioblastoma as a "diffusely infiltrating, highly cellular glioma" (Histopathology section under Glioblastoma, IDH-wildtype).

2. Nuclear features: The "angular and hyperchromatic nuclei" and "rounded" nuclei in some areas are consistent with the description of glioblastoma, which can show "poorly differentiated, fusiform, round, or pleomorphic cells" (Histopathology section).

3. Mitotic activity: "Mitotic figures are frequently observed," which aligns with "Mitotic activity is readily identifiable in most cases and is often brisk" (Histopathology section).

4. Microvascular proliferation: "Multiple zones of microvascular endothelial proliferation are discernible," which is a key diagnostic feature of glioblastoma (Histopathology section).

5. Necrosis: "Sporadic regions of necrosis with pseudopalisading features are identified," another characteristic feature of glioblastoma (Histopathology section).

6. IDH status: "Immunostaining shows tumour cells are negative for IDH1 mutant (R132H)," which is consistent with the IDH-wildtype status (Diagnostic molecular pathology section).

7. ATRX status: "ATRX is retained within the tumour cell nuclei," which is typical for IDH-wildtype glioblastoma (Immunophenotype section).

8. Ki-67 index: "The Ki-67 index demonstrates a considerable proliferative activity, approximately 15 to 20%," which is consistent with the high proliferative activity seen in glioblastomas (Proliferation subsection under Histopathology).

The essential diagnostic criteria for Glioblastoma, IDH-wildtype are met, as stated in the document: "Glioblastoma, IDH-wildtype is an IDH-wildtype and H3-wildtype diffusely astrocytic glioma. Furthermore, this tumour must display at least one or more of the following: microvascular proliferation, necrosis, TERT promotor mutation, EGFR gene amplification, +7/-10 chromosome copy-number alterations." (Essential and desirable diagnostic criteria section)

In this case, the tumor displays both microvascular proliferation and necrosis, meeting the criteria for the diagnosis of Glioblastoma, IDH-wildtype, CNS WHO grade 4.

ChatGPT Response:

Based on the histopathological description provided, the diagnosis is likely a Glioblastoma (GBM), IDH-wildtype. Here is the reasoning:

1. **Histopathological Features**:
   - **Infiltrative Tumour**: Glioblastomas are highly infiltrative.
   - **Angular and Hyperchromatic Nuclei**: Typical for malignant gliomas.
   - **Mitotic Figures**: Frequent mitoses are indicative of a high-grade tumour.
   - **Microvascular Endothelial Proliferation**: A hallmark of glioblastoma.
   - **Necrosis with Pseudopalisading**: Another hallmark feature of glioblastoma.
2. **Immunohistochemical Staining**:
   - **Negative for IDH1 mutant (R132H)**: IDH-wildtype, which is associated with primary (de novo) glioblastomas.
   - **Retention of ATRX**: Loss of ATRX is more common in lower-grade gliomas and IDH-mutant astrocytomas, so its retention supports the diagnosis of GBM, IDH-wildtype.
   - **Ki-67 Index of 15-20%**: Indicates a high proliferative activity, consistent with a high-grade tumour such as glioblastoma.
3. **Absence of Sarcomatous Transformation**: Rules out gliosarcoma, a variant of glioblastoma.

Given these features, the tumour is best classified as **Glioblastoma, WHO Grade 4, IDH-wildtype**.

ChatGPT RAG response

Based on the histopathological description provided and using the WHO diagnostic guidelines for CNS tumors:

1. **Infiltrative tumor**: This indicates the tumor spreads diffusely through the brain tissue, which is common in high-grade gliomas.
2. **Angular and hyperchromatic nuclei within a fibrillary matrix**: These are typical features of astrocytic tumors.
3. **Mitotic figures frequently observed**: High mitotic activity suggests a high-grade tumor.
4. **Microvascular endothelial proliferation**: This is a hallmark of glioblastoma.
5. **Necrosis with pseudopalisading**: Another characteristic feature of glioblastoma.
6. **Immunostaining negative for IDH1 mutant (R132H)**: Indicates an IDH-wildtype tumor.
7. **Retained ATRX**: Typically excludes astrocytoma with ATRX loss, pointing towards IDH-wildtype glioblastoma.
8. **Ki-67 index of 15 to 20%**: High proliferative activity consistent with a high-grade glioma.

Given these characteristics, the most likely diagnosis is **Glioblastoma, IDH-wildtype (CNS WHO grade 4)**.

References:
- **Microvascular proliferation and necrosis** are essential criteria for diagnosing glioblastoma according to the WHO guidelines【3†source】.
- **IDH-wildtype and retained ATRX** status further supports the diagnosis of glioblastoma IDH-wildtype as opposed to other astrocytic tumors【3†source】.
- **High mitotic activity and high Ki-67 index** align with the aggressive nature of glioblastoma【3†source】.

Thus, the diagnosis, based on the provided histopathological description and the WHO diagnostic criteria, is **Glioblastoma, IDH-wildtype, CNS WHO grade 4**.

G9: NH19 1880 - A2 IDHwt

The histology shows multiple small specimens of a glial tumour, which is moderately cellular and diffuse. The neoplastic cells possess small, rounded nuclei with moderate chromatin density and pleomorphism. These cells are interspersed within a fibrillary matrix and infiltrate into the adjacent CNS parenchyma. No distinctively high-grade features were identified on microscopic evaluation; notably, neither angiogenesis nor necrosis were present.

Immunohistochemical staining for IDH1 R132H mutant was negative. ATRX expression in the tumour cell nuclei was retained and the Ki-67 proliferation index was low, approximating 1%. Molecular analysis showed that TERT (C228T) was mutated. Testing for EGFR amplification was inconclusive.

What do you think the diagnosis would be?

GT: Glioblastoma, IDH-wildtype

Llama Response:

Based on the histopathological description, I would diagnose this tumour as a **Diffuse Astrocytoma, WHO Grade II**.

Here's my reasoning:

- The tumour is described as moderately cellular and diffuse, which is consistent with a low-grade astrocytoma.
- The presence of small, rounded nuclei with moderate chromatin density and pleomorphism suggests a low-grade glioma.
- The absence of high-grade features such as angiogenesis and necrosis further supports a low-grade diagnosis.
- The negative IDH1 R132H mutant immunohistochemical staining suggests that this is not an IDH-mutated astrocytoma, which is more commonly seen in high-grade gliomas.
- The retained ATRX expression in the tumour cell nuclei suggests that this tumour is not an astrocytoma with a 1p/19q co-deletion, which is more commonly seen in oligodendrogliomas.
- The low Ki-67 proliferation index (1%) is consistent with a low-grade glioma.
- The presence of a TERT (C228T) mutation is also consistent with a low-grade astrocytoma.
- The inconclusive EGFR amplification testing is not inconsistent with a diffuse astrocytoma diagnosis.

Overall, based on the combination of morphological and immunohistochemical features, I believe the most likely diagnosis is a Diffuse Astrocytoma, WHO Grade II.

Llama RAG response:

Based on the histopathological description and immunohistochemical staining results, I believe the diagnosis is an **IDH-mutant astrocytoma, grade 2**.

Here's the reasoning:

- The tumour is described as moderately cellular and diffuse, with small, rounded nuclei and pleomorphism, which is consistent with an astrocytic tumour (Introduction to CNS Tumours, p. 2).
- The lack of high-grade features, such as angiogenesis and necrosis, suggests a lower-grade tumour (CNS Tumour Grading, p. 5).
- The negative IDH1 R132H mutant staining suggests that the tumour is not an IDH-wildtype glioblastoma (Gene and Protein Nomenclature, p. 3).
- The retained ATRX expression in the tumour cell nuclei is consistent with an IDH-mutant astrocytoma (CNS Tumour Nomenclature, p. 2).
- The low Ki-67 proliferation index of 1% is consistent with a lower-grade tumour (CNS Tumour Grading, p. 5).
- The TERT (C228T) mutation is consistent with an IDH-mutant astrocytoma (Novel Diagnostic Technologies, p. 9).
- The inconclusive EGFR amplification testing does not affect the diagnosis.

According to the WHO classification, an IDH-mutant astrocytoma, grade 2, is a tumour that is characterized by a combination of histological and molecular features, including IDH mutation, retained ATRX expression, and a low Ki-67 proliferation index (CNS Tumour Grading, p. 5).

Reference: World Health Organisation Classification of Tumours, 5th Edition.

Claude Response:

Based on the histopathological description and molecular findings provided, the most likely diagnosis is:

Diffuse Astrocytoma, IDH-wildtype, WHO grade 2

Reasoning:
1. Histology:
   - Glial tumor, moderately cellular and diffuse
   - Infiltrative growth pattern into adjacent CNS parenchyma
   - Small, rounded nuclei with moderate chromatin density and pleomorphism
   - Fibrillary matrix
   - Absence of high-grade features (no angiogenesis or necrosis)
2. Immunohistochemistry and Molecular Findings:
   - IDH1 R132H mutation negative
   - ATRX expression retained
   - Low Ki-67 proliferation index (approximately 1%)
   - TERT (C228T) mutation present
   - EGFR amplification inconclusive

The diffuse growth pattern, infiltrative nature, and moderate cellularity are consistent with a diffuse glioma. The absence of high-grade features and low Ki-67 index suggest a lower-grade tumor (WHO grade 2).

The IDH-wildtype status is based on the negative IDH1 R132H immunohistochemistry. While this doesn't rule out other, less common IDH mutations, the presence of a TERT promoter mutation is more commonly associated with IDH-wildtype gliomas.

The retained ATRX expression and TERT mutation are more consistent with an astrocytic rather than oligodendroglial lineage.

It's important to note that the 2021 WHO classification of CNS tumors has introduced molecular criteria for adult-type diffuse gliomas. In some cases, IDH-wildtype diffuse astrocytomas with certain molecular features (like TERT promoter mutation) may be considered as having "molecular features of glioblastoma, WHO grade 4." However, given the low-grade histological appearance and low Ki-67 index in this case, a grade 2 designation is more appropriate.

If additional molecular testing is available (such as copy number profiling or CDKN2A/B status), it could further refine the diagnosis and grading.

Claude RAG response:

Based on the histopathological description and molecular findings provided, and using only the information in the attached WHO diagnostic guidelines document, I believe the most likely diagnosis is:

Glioblastoma, IDH-wildtype, CNS WHO grade 4

Here's my reasoning, with references to the attached document:

1. The tumor is described as a glial tumor with diffuse growth and infiltration into adjacent CNS parenchyma, which is consistent with a diffuse astrocytic glioma.
2. The IDH1 R132H immunohistochemistry was negative. According to the document, "Absence of immunoreactivity for IDH1 p.R132H is sufficient (i.e. without further sequencing) to diagnose IDH-wildtype glioblastoma in a patient aged ≥ 55 years at diagnosis who has a histologically classic glioblastoma not located in midline structures and no history of a pre-existing lower-grade glioma."
3. While the histology doesn't show typical high-grade features like microvascular proliferation or necrosis, the document states that for IDH-wildtype glioblastoma: "In an IDH- and H3-wildtype diffuse glioma, at least one of these features (i.e. microvascular proliferation or necrosis) is sufficient for the diagnosis of glioblastoma."
4. Crucially, the molecular analysis showed a TERT promoter mutation (C228T). The document states: "The presence of at least one of these aberrations [TERT promoter mutations, EGFR gene amplification, and a +7/−10 genotype] in an IDH- and H3-wildtype diffuse glioma allows for the diagnosis of IDH-wildtype glioblastoma even in the absence of microvascular proliferation and/or necrosis."
5. The retained ATRX expression is consistent with IDH-wildtype glioblastoma, as the document notes: "Nuclear immunostaining for ATRX is retained in the vast majority of tumours."

While the low Ki-67 index and lack of high-grade histological features might suggest a lower-grade tumor, the presence of the TERT promoter mutation in an IDH-wildtype diffuse glioma is sufficient for the diagnosis of glioblastoma according to the current WHO guidelines.

It's worth noting that if the patient is younger than 55, additional sequencing for less common IDH mutations and testing for H3 K27M and H3 G34 mutations might be warranted to definitively exclude other glioma types. However, based on the information provided, glioblastoma, IDH-wildtype appears to be the most appropriate diagnosis.

ChatGPT Response:

Based on the histopathological and molecular characteristics provided, the most likely diagnosis is **Diffuse Astrocytoma, IDH-wildtype**.

Here is the rationale for this diagnosis:
1. **Histopathological Features**:
   - **Moderately Cellular and Diffuse**: Suggests a diffuse glioma.
   - **Small, Rounded Nuclei with Moderate Chromatin Density and Pleomorphism**: Typical of lower-grade gliomas.
   - **Fibrillary Matrix and Infiltration into Adjacent CNS Parenchyma**: Common in astrocytic tumours.
   - **Absence of High-Grade Features (Angiogenesis, Necrosis)**: Suggests a lower-grade glioma (Grade II or III).
2. **Immunohistochemistry and Molecular Features**:
   - **IDH1 R132H Negative**: Suggests an IDH-wildtype tumour.
   - **ATRX Expression Retained**: Retained ATRX is more typical in IDH-wildtype tumours.
   - **Low Ki-67 Proliferation Index (1%)**: Indicates a lower proliferative rate, consistent with lower-grade tumours.
   - **TERT (C228T) Mutation**: TERT promoter mutations are often associated with IDH-wildtype diffuse astrocytomas.
   - **EGFR Amplification Inconclusive**: EGFR amplification is more commonly associated with higher-grade gliomas, but its inconclusiveness does not rule out the diagnosis.

Given these findings, the most appropriate diagnosis is ****Diffuse Astrocytoma, IDH-wildtype, WHO Grade II****. However, it is important to note that the presence of a TERT promoter mutation can sometimes be seen in higher-grade gliomas, and further clinical and radiological correlation is advised to fully determine the tumour grade and management plan.

ChatGPT RAG response:

Based on the histopathological description and immunohistochemical findings provided, the diagnosis can be deduced as follows:

1. **Histopathology**:
   - **Moderately cellular glial tumor** with small, rounded nuclei and moderate chromatin density.
   - Diffuse infiltration within the CNS parenchyma.

- Absence of high-grade features such as angiogenesis and necrosis.

2. **Immunohistochemistry and Molecular Findings**:
   - **IDH1 R132H mutant negative**.
   - **Retained ATRX expression**.
   - **Low Ki-67 proliferation index** (~1%).
   - **TERT (C228T) mutation present**.
   - **Inconclusive EGFR amplification**.

The provided histopathological features and molecular findings suggest the following diagnostic pathway:

- **IDH-wildtype gliomas** can be identified by the lack of IDH1 mutation.
- **Retained ATRX expression** indicates it is not an astrocytoma with ATRX mutation.
- **TERT promoter mutation** and the absence of high-grade features such as necrosis and microvascular proliferation are important diagnostic clues.

Given the lack of IDH mutation, retained ATRX expression, and presence of a TERT promoter mutation, along with the histopathological features described, the most likely diagnosis is **Diffuse Astrocytoma, IDH-wildtype, CNS WHO grade 2**.

Diagnostic Justification:
- **Diffuse Astrocytoma, IDH-wildtype (CNS WHO grade 2)** is characterized by moderate cellularity, lack of high-grade features (no necrosis or angiogenesis), and low proliferation index. It is also typically negative for IDH1 mutations.
- The presence of a TERT promoter mutation supports the diagnosis, as TERT promoter mutations are often associated with IDH-wildtype gliomas.

References:
- **Histopathological characteristics of diffuse astrocytomas, IDH-wildtype** are outlined in the document. These tumors are typically IDH1-wildtype, retain ATRX expression, and can present TERT promoter mutations【3†source】.
- **CNS WHO grading** for diffuse astrocytomas includes grading based on histological features and molecular characteristics【3†source】.

Thus, the diagnosis for this tumor based on the given information is likely **Diffuse Astrocytoma, IDH-wildtype, CNS WHO grade 2**.

G10: NH19 2048 - A3 IDHwt

Histological examination reveals small sections consisting primarily of white matter with an elevated cellular content, attributed to an infiltration of pleomorphic glial cells. These cells have ill-defined cytoplasmic borders with irregular and hyperchromatic nuclei. The tumour cells lie in patternless sheets within a background fibrillary stroma. Mitotic figures are evident, however microvascular proliferation and necrosis are not seen, within the limitations of this small sample.

Immunohistochemical testing for IDH1 (R132H) mutation was negative. ATRX nuclear expression was retained. The H3K27me3 immunostaining was inconsistent, with some tumour cells exhibiting minimal to negligible staining intensity while others displayed intense nuclear expression. The Ki67 proliferation index is substantial at approximately 10%, considering the modest cellularity.

Molecular testing revealed C228T TERT mutation and low-level amplification of EGFR.

What do you think the diagnosis would be?

GT: glioblastoma, IDH-wildtype

Llama Response:

What a fascinating case!

Based on the histopathological description, I would venture to say that the diagnosis is an **Anaplastic Astrocytoma, Grade III**.

Here's my reasoning:

1. The presence of pleomorphic glial cells with ill-defined cytoplasmic borders and irregular, hyperchromatic nuclei suggests a high-grade glioma.
2. The patternless sheets of tumour cells within a fibrillary stroma are consistent with an astrocytic tumour.
3. The lack of microvascular proliferation and necrosis, despite the presence of mitotic figures, suggests an anaplastic astrocytoma rather than a glioblastoma.
4. The negative IDH1 (R132H) mutation and retained ATRX nuclear expression argue against an IDH-mutant astrocytoma.
5. The inconsistent H3K27me3 immunostaining, with some tumour cells exhibiting minimal to negligible staining intensity, is consistent with an anaplastic astrocytoma.
6. The substantial Ki67 proliferation index of approximately 10% supports a high-grade tumour.
7. The presence of a C228T TERT mutation is commonly seen in high-grade gliomas, including anaplastic astrocytomas.
8. The low-level amplification of EGFR is also consistent with an anaplastic astrocytoma.

Given the above features, I would diagnose this tumour as an Anaplastic Astrocytoma, Grade III.

Llama RAG response:
Based on the histopathological description and immunohistochemical testing, I think the diagnosis is an **IDH-wildtype glioblastoma, grade 4**.

Here's why:
- The presence of pleomorphic glial cells with ill-defined cytoplasmic borders and hyperchromatic nuclei, along with the patternless sheets of tumour cells, suggests a high-grade glial neoplasm (WHO Classification of Tumours, p. 14).
- The absence of microvascular proliferation and necrosis may suggest a lower grade tumour, but the presence of mitotic figures and the Ki67 proliferation index of 10% suggest a more aggressive tumour (WHO Classification of Tumours, p. 14).
- The negative IDH1 (R132H) mutation test and retained ATRX nuclear expression suggest an IDH-wildtype glioblastoma (WHO Classification of Tumours, p. 15).
- The inconsistent H3K27me3 immunostaining is not inconsistent with an IDH-wildtype glioblastoma diagnosis (WHO Classification of Tumours, p. 15).
- The presence of a C228T TERT mutation and low-level amplification of EGFR are both seen in IDH-wildtype glioblastomas (WHO Classification of Tumours, p. 16).

Given the combination of these features, I believe the diagnosis is an IDH-wildtype glioblastoma, grade 4.

References:

* WHO Classification of Tumours, p. 14 (CNS tumour grading)
* WHO Classification of Tumours, p. 15 (IDH-wildtype glioblastoma)
* WHO Classification of Tumours, p. 16 (molecular parameters in IDH-wildtype glioblastoma)

Claude Response:
Based on the histopathological description and molecular findings provided, the most likely diagnosis is:

Diffuse Astrocytoma, IDH-wildtype, WHO grade 4 (also known as Glioblastoma, IDH-wildtype)

Here's the rationale:
1. Histology:
    - Infiltrative growth in white matter
    - Pleomorphic glial cells with hyperchromatic nuclei
    - Fibrillary background
    - Presence of mitotic figures
    - Absence of microvascular proliferation and necrosis (which could be due to sampling limitations)
2. Immunohistochemistry:
    - IDH1 (R132H) mutation negative
    - Retained ATRX nuclear expression
    - Variable H3K27me3 staining
    - High Ki67 proliferation index (10%)

3. Molecular findings:
- TERT promoter mutation (C228T)
- Low-level EGFR amplification

The combination of IDH-wildtype status, TERT promoter mutation, and EGFR amplification strongly suggests a diagnosis of IDH-wildtype glioblastoma, even in the absence of microvascular proliferation or necrosis in this sample. The 2021 WHO classification of CNS tumors allows for the diagnosis of IDH-wildtype glioblastoma based on molecular features, even when classic histological features are not present.

The high Ki67 index and the presence of mitotic figures support a high-grade designation. The variable H3K27me3 staining is not typical for a specific entity but can be seen in various high-grade gliomas.

While the histology alone might suggest a lower-grade astrocytoma, the molecular findings, particularly the TERT promoter mutation and EGFR amplification, are characteristic of IDH-wildtype glioblastoma and warrant the WHO grade 4 designation.

Claude RAG response:

Based on the histopathological description and molecular findings provided, and referring to the WHO diagnostic guidelines in the attached document, the most likely diagnosis is:

Glioblastoma, IDH-wildtype, CNS WHO grade 4

Reasoning:

1. Histology: The description indicates a diffusely infiltrating astrocytic glioma with pleomorphic glial cells and mitotic figures. While microvascular proliferation and necrosis are not seen, this could be due to the small sample size.
2. IDH status: The tumor is IDH-wildtype, as indicated by the negative IDH1 (R132H) immunohistochemistry. The document states: "IDH-wildtype glioblastomas lack immunostaining for IDH1 p.R132H" (Glioblastoma, IDH-wildtype section, Immunophenotype subsection).
3. ATRX status: Nuclear ATRX expression is retained, which is consistent with IDH-wildtype glioblastoma. The document notes: "Nuclear immunostaining for ATRX is retained in the vast majority of tumours" (Glioblastoma, IDH-wildtype section, Immunophenotype subsection).
4. Molecular findings: The presence of TERT promoter mutation (C228T) and EGFR amplification strongly support the diagnosis of IDH-wildtype glioblastoma. The document states: "Frequent and diagnostically relevant molecular alterations in IDH-wildtype glioblastomas include TERT promoter mutations, EGFR gene amplification, and a +7/−10 genotype. The presence of at least one of these aberrations in an IDH- and H3-wildtype diffuse glioma allows for the diagnosis of IDH-wildtype glioblastoma even in the absence of microvascular proliferation and/or necrosis." (Glioblastoma, IDH-wildtype section, Diagnostic molecular pathology subsection).
5. Ki67 index: The high Ki67 proliferation index (10%) is consistent with a high-grade glioma.
6. H3K27me3 staining: The inconsistent H3K27me3 staining does not specifically point to a H3 K27-altered diffuse midline glioma, which would show more uniform loss of H3K27me3 staining.

While the absence of microvascular proliferation and necrosis might suggest a lower grade, the molecular findings (TERT promoter mutation and EGFR amplification) are sufficient for the diagnosis of glioblastoma, IDH-wildtype, even in the absence of these histological features, according to the current WHO guidelines.

In conclusion, based on the integration of histological, immunohistochemical, and molecular findings as described in the WHO guidelines document, the diagnosis of Glioblastoma, IDH-wildtype, CNS WHO grade 4 is most appropriate for this tumor.

ChatGPT Response:

The histopathological and molecular findings are suggestive of a diffuse glioma, specifically a glioblastoma, IDH-wildtype. Here's a breakdown of the key features supporting this diagnosis:

1. **Histopathological Features**:
   - **Pleomorphic glial cells**: Indicative of high-grade gliomas such as glioblastoma.
   - **Irregular and hyperchromatic nuclei**: Common in high-grade gliomas.
   - **Mitotic figures**: Evidence of cell proliferation, typical of high-grade gliomas.
   - **Patternless sheets within a fibrillary stroma**: Consistent with glioblastoma morphology.
   - **Absence of microvascular proliferation and necrosis**: These features are common in glioblastoma but their absence does not exclude the diagnosis, especially in small samples.
2. **Immunohistochemistry**:
   - **IDH1 (R132H) negative**: Indicates an IDH-wildtype status.
   - **Retained ATRX expression**: This finding, while often seen in IDH-mutant gliomas, does not exclude glioblastoma as retained ATRX expression can occur in IDH-wildtype glioblastomas.
   - **H3K27me3 inconsistent staining**: This finding can be variable and is not definitive for a specific glioma subtype.
   - **Ki67 proliferation index of 10%**: Indicates a higher proliferative activity, consistent with high-grade glioma.
3. **Molecular Testing**:
   - **C228T TERT mutation**: Frequently found in glioblastomas, IDH-wildtype.
   - **Low-level amplification of EGFR**: Common in glioblastomas, particularly in IDH-wildtype cases.

Based on these findings, the most likely diagnosis is ****glioblastoma, IDH-wildtype****. The absence of necrosis and microvascular proliferation in the small sample does not rule out glioblastoma, as these features can be focal and may not be present in all sections. The molecular profile, particularly the presence of a TERT mutation and EGFR amplification, strongly supports this diagnosis.

Chat GPT RAG response:

Based on the provided histopathological description and immunohistochemical/molecular findings, I will determine the type and grade of the tumour using the WHO Classification of Tumours of the Central Nervous System document.

Histopathological Description and Immunohistochemical Findings:
- **Cellular Content**: Elevated cellularity with pleomorphic glial cells
- **Cell Borders and Nuclei**: Ill-defined cytoplasmic borders, irregular and hyperchromatic nuclei
- **Mitotic Figures**: Present
- **Microvascular Proliferation and Necrosis**: Absent in the sample
- **IDH1 (R132H)**: Negative
- **ATRX**: Nuclear expression retained
- **H3K27me3**: Inconsistent staining
- **Ki67 Proliferation Index**: Approximately 10%
- **Molecular Findings**: C228T TERT mutation, low-level amplification of EGFR

Analysis:
IDH-wildtype Glioblastoma:
- The tumour does not have an IDH1 mutation, as IDH1 (R132H) was negative.
- Retained ATRX nuclear expression is also consistent with IDH-wildtype status.
- The presence of a TERT promoter mutation (C228T) is a significant indicator.
- Low-level amplification of EGFR further supports this diagnosis.
- The absence of microvascular proliferation and necrosis is noted, but the presence of these features is not a strict requirement for diagnosis when specific molecular markers are present.
- The Ki67 index at approximately 10% indicates substantial proliferative activity.

According to the WHO guidelines:
- Glioblastoma, IDH-wildtype, can be diagnosed based on the presence of certain genetic markers even in the absence of histological features such as microvascular proliferation or necrosis.

Relevant WHO Criteria:
- Glioblastoma IDH-wildtype can be diagnosed in the presence of IDH-wildtype status and one or more of the following: microvascular proliferation, necrosis, TERT promoter mutation, EGFR amplification, or +7/−10 chromosome copy-number changes (CNS WHO grade 4)【3†source】.

Conclusion:
The diagnosis is **Glioblastoma, IDH-wildtype, CNS WHO grade 4**.

References:
- WHO Classification of Tumours of the Central Nervous System, 5th Edition
- Glioblastoma IDH-wildtype diagnostic criteria (includes molecular markers such as TERT promoter mutation and EGFR amplification)【3†source】.

Oligodendroglioma Cases

O1: NH18-667 - O2+FISH

Histology shows a large fragment of brain parenchyma composed of cortex and subcortical white matter. In sections of the white matter, the cellularity is mild- to moderately increased. This is due to an infiltration of monomorphic glial cells with indistinct cell boundaries, rounded nuclei and speckled chromatin. Perinuclear haloes are not a feature. Mitotic figures, microvascular proliferation and necrosis are not readily seen.

Immunohistochemistry
IDH1 (R132H): positive
ATRX: nuclear expression is retained in tumour nuclei
Ki67 (proliferation): low, up to 5%

FISH: 1p19q co-deletion

What do you think the diagnosis would be?

GT: Oligodendroglioma, IDH-mutant and 1p/19q-codeleted, CNS WHO grade 2

Llama Response:

A fascinating case!

Based on the histopathological description and the immunohistochemical and FISH findings, I would venture to say that the diagnosis is an ****Oligodendroglioma, WHO Grade II****.

Here's why:

- The presence of monomorphic glial cells with indistinct cell boundaries, rounded nuclei, and speckled chromatin suggests an oligodendroglial tumor.
- The absence of perinuclear haloes, mitotic figures, microvascular proliferation, and necrosis points towards a low-grade tumor.
- The positive IDH1 (R132H) immunostain supports the diagnosis of an oligodendroglioma, as this mutation is common in these tumors.
- The retained nuclear expression of ATRX in tumor nuclei is also consistent with an oligodendroglioma.
- The low Ki67 proliferation index (up to 5%) is indicative of a low-grade tumor.
- The 1p19q co-deletion, as detected by FISH, is a characteristic genetic hallmark of oligodendrogliomas.
- The combination of these features, along with the absence of high-grade features, suggests a WHO Grade II oligodendroglioma.

What do you think? Am I correct?

Llama RAG response:

Based on the histopathological description and immunohistochemical results, I believe the diagnosis is:

****Oligodendroglioma, IDH-mutant and 1p/19q codeleted, Grade 2****

Here's my reasoning, supported by the attached WHO Classification of Tumours document:

1. **Morphology**: The description of monomorphic glial cells with indistinct cell boundaries, rounded nuclei, and speckled chromatin suggests an oligodendroglial tumor (Page 14, CNS Tumour Nomenclature).
2. **IDH1 mutation**: The positive IDH1 (R132H) immunostain indicates an IDH-mutant tumor, which is characteristic of oligodendrogliomas (Page 14, CNS Tumour Nomenclature).
3. **Retention of ATRX expression**: The retained nuclear expression of ATRX in tumor nuclei is consistent with an oligodendroglioma (Page 15, CNS Tumour Nomenclature).
4. **Ki67 index**: The low Ki67 index (up to 5%) indicates a relatively low proliferative activity, which is consistent with a Grade 2 oligodendroglioma (Page 18, CNS Tumour Grading).
5. **1p/19q codeletion**: The presence of 1p19q codeletion, as detected by FISH, is a characteristic genetic feature of oligodendrogliomas (Page 14, CNS Tumour Nomenclature).

Given the combination of these features, I believe the diagnosis is an Oligodendroglioma, IDH-mutant and 1p/19q codeleted, Grade 2.

Please let me know if you have any questions or if you'd like me to clarify any aspect of the diagnosis!

Claude Response:

Based on the histopathological description and immunohistochemistry results provided, the diagnosis is most likely:

Oligodendroglioma, IDH-mutant and 1p/19q-codeleted, WHO grade 2

Here's the reasoning behind this diagnosis:

1. Histology:
    - Infiltrative growth in white matter
    - Mild to moderate increase in cellularity
    - Monomorphic glial cells with rounded nuclei and speckled chromatin
    - Absence of significant mitotic activity, microvascular proliferation, and necrosis
2. Immunohistochemistry and molecular findings:
    - IDH1 (R132H) mutation: positive
    - ATRX: retained nuclear expression (not mutated)
    - Ki67: low proliferation index (up to 5%)
    - FISH: 1p/19q co-deletion present

The combination of IDH mutation and 1p/19q co-deletion is diagnostic for oligodendroglioma according to the WHO classification. The low-grade features (absence of significant mitotic activity, microvascular proliferation, and necrosis) and low Ki67 index support a grade 2 designation.
This tumor fits the criteria for a WHO grade 2 oligodendroglioma, which is characterized by slow growth and better prognosis compared to higher-grade gliomas.

Claude RAG response:

Based on the histopathological description and immunohistochemistry results provided, and using only the information from the attached WHO diagnostic guidelines document, I believe the diagnosis is:

Oligodendroglioma, IDH-mutant and 1p/19q-codeleted, CNS WHO grade 2

Here's my reasoning, referencing the attached document:

1. IDH mutation: The tumor is IDH1 (R132H) positive, which indicates an IDH mutation. This is a key feature of oligodendroglioma according to the definition: "Oligodendroglioma, IDH-mutant and 1p/19q-codeleted, is a diffusely infiltrating glioma with IDH1 or IDH2 mutation and codeletion of chromosome arms 1p and 19q" (from the "Definition" section under "Oligodendroglioma, IDH-mutant and 1p/19q-codeleted").
2. 1p/19q codeletion: FISH shows 1p19q co-deletion, which is the second defining feature of oligodendroglioma.
3. Histology: The description of "monomorphic glial cells with indistinct cell boundaries, rounded nuclei and speckled chromatin" is consistent with the histopathological features of oligodendroglioma described in the document: "Classic oligodendroglioma cells have uniformly round nuclei that are slightly larger than those of normal oligodendrocytes and show an increase in chromatin density or a delicate salt-and-pepper pattern" (from the "Histopathology" section).
4. ATRX: Nuclear expression of ATRX is retained, which is consistent with oligodendroglioma. The document states: "IDH-mutant and 1p/19q-codeleted oligodendrogliomas retain nuclear expression of ATRX" (from the "Immunophenotype" subsection).
5. Grade: The absence of mitotic figures, microvascular proliferation, and necrosis, along with the low Ki67 proliferation index (up to 5%), suggests a lower grade. The document states for grade 2: "Mitotic activity is absent or uncommon in CNS WHO grade 2 tumours" and "The growth fraction as determined by the Ki-67 proliferation index is usually < 4% for CNS WHO grade 2 IDH-mutant astrocytomas" (from the "Proliferation" subsection under "Astrocytoma, IDH-mutant", which can be used as a reference for proliferation in grade 2 gliomas).

In conclusion, the combination of IDH mutation, 1p/19q codeletion, histological features, and low proliferation index all point to a diagnosis of Oligodendroglioma, IDH-mutant and 1p/19q-codeleted, CNS WHO grade 2.

ChatGPT Response:

Based on the histopathological description provided, the diagnosis is likely **oligodendroglioma, WHO Grade II**.

Here's how this conclusion is reached:
1. **Histopathological Features:**
   - The presence of monomorphic glial cells with indistinct cell boundaries and rounded nuclei suggests a glial tumor.
   - The absence of perinuclear halos makes it less typical for oligodendroglioma, but not definitive against it, especially in lower-grade tumors.
   - Lack of mitotic figures, microvascular proliferation, and necrosis aligns with a lower-grade (Grade II) glioma.
2. **Immunohistochemistry:**
   - **IDH1 (R132H) positive:** This mutation is common in lower-grade gliomas, including oligodendrogliomas.
   - **ATRX retention:** This suggests that the tumor is more likely an oligodendroglioma, as ATRX mutations are typically seen in astrocytomas.
   - **Ki67 (proliferation) low, up to 5%:** Indicates a low-grade tumor.
3. **Genetic Studies:**
   - **1p19q co-deletion:** This is a defining feature of oligodendrogliomas and distinguishes them from astrocytomas, which do not typically have this co-deletion.

Given the combination of histopathological features, immunohistochemical findings, and genetic results, the tumor is best classified as an ****oligodendroglioma, WHO Grade II****.

ChatGPT RAG response:

Based on the provided histopathological description and immunohistochemistry results, the diagnosis is **Oligodendroglioma, IDH-mutant and 1p/19q-codeleted, CNS WHO grade 2**.

Justification:
1. **Histological Features**:
   - Mild to moderate increase in cellularity due to infiltration of monomorphic glial cells.
   - Rounded nuclei and speckled chromatin.
   - Absence of perinuclear haloes.
   - No mitotic figures, microvascular proliferation, or necrosis observed.
2. **Immunohistochemistry**:
   - **IDH1 (R132H) positive**: Indicates the presence of an IDH mutation, which is a characteristic of oligodendrogliomas.
   - **ATRX retained**: Retained ATRX expression supports the diagnosis of oligodendroglioma over astrocytoma, as loss of ATRX expression is commonly associated with astrocytomas.
   - **Ki67 proliferation index low, up to 5%**: Supports a lower-grade tumor (CNS WHO grade 2).
3. **Genetic Testing**:
   - **1p/19q co-deletion**: Confirms the diagnosis of oligodendroglioma, as this genetic alteration is a defining feature.

References:
- The definition and features of **Oligodendroglioma, IDH-mutant and 1p/19q-codeleted** are described in the WHO Classification of Tumours of the Central Nervous System, fifth edition:

- "Oligodendroglioma IDH-mutant and 1p/19q-codeleted is a diffusely infiltrating glioma with IDH1 or IDH2 mutation and codeletion of chromosome arms 1p and 19q (CNS WHO grade 2 or 3)"【3†source】.
- Histologically, "Classic oligodendroglioma cells have uniformly round nuclei, a slight increase in chromatin density, and lack perinuclear haloes"【3†source】.
- Immunophenotype: "Most oligodendrogliomas demonstrate immunoreactivity with the antibody against IDH1 p.R132H" and "retained nuclear expression of ATRX"【3†source】
- "Mitotic activity is low or absent in CNS WHO grade 2 oligodendrogliomas" and "the Ki-67 (MIB1) proliferation index is usually low (< 5%) in CNS WHO grade 2 oligodendrogliomas"【3†source】.

These features and results align with the diagnosis of **Oligodendroglioma, IDH-mutant and 1p/19q-codeleted, CNS WHO grade 2**.

O2: N11 1318 - O2+mol

Several fragments of a moderately dense glial tumour plus fragments of CNS tissue, which are loosely infiltrated by tumour cells. The tumour contains small areas with a rounded tumour cell morphology and a loose cytoplasm. The nuclei here have a fine dotted chromatin. In other area, occasional perinuclear halos are seen. The rest of the tumour displays a peculiar astrocytic phenotype with frequent Rosenthal-like inclusions in astrocytes.
Immunostaining for mutant IDH-1 reveals that the small oligodendroglial component and the minor astrocytic component of the tumour labelled positive. The fragments of CNS tissue and also the areas with Rosenthal fibres do not express mutant IDH-1 protein. Molecular pathology revealed co-deletion of 1p and 19q.

What do you think the diagnosis would be?

GT: Oligodendroglioma, IDH-mutant and 1p/19q-codeleted, CNS WHO grade 2

Llama Response:

What a fascinating histopathological description!

Based on the description, I think the diagnosis is an **Oligodendroglioma, likely grade II**.

Here's why:
- The presence of small, rounded tumour cells with fine dotted chromatin and perinuclear halos is consistent with oligodendroglial morphology.
- The astrocytic phenotype with Rosenthal-like inclusions is also consistent with oligodendrogliomas, which can have a mixed glioma component.
- The positive immunostaining for mutant IDH-1 in the oligodendroglial component is a strong indicator of an oligodendroglioma, as IDH-1 mutations are common in these tumours.
- The co-deletion of 1p and 19q, a genetic hallmark of oligodendrogliomas, further supports this diagnosis.

Given the lack of high-grade features, such as necrosis, microvascular proliferation, or brisk mitotic activity, I would grade this tumour as low-grade (II) rather than anaplastic (III) or glioblastoma (IV).

What do you think? Am I correct?

Llama RAG response:

Based on the histopathological description and the attached document "World Health Organisation Classification of Tumours.docx", I believe the diagnosis is an **Oligodendroglioma, IDH-mutant and 1p/19q-codeleted, grade 2**.

References:

- The presence of small, rounded tumour cells with fine dotted chromatin and occasional perinuclear halos suggests an oligodendroglial component (Section "CNS Tumour Nomenclature" and "Grading within Types").
- The astrocytic phenotype with Rosenthal-like inclusions and the absence of mutant IDH-1 expression in these areas suggest a minor astrocytic component (Section "CNS Tumour Nomenclature").
- The immunostaining for mutant IDH-1 reveals that the small oligodendroglial component and the minor astrocytic component of the tumour labelled positive, which supports the diagnosis of an IDH-mutant oligodendroglioma (Section "Gene and Protein Nomenclature" and "Clinicopathological grading").
- The molecular pathology revealing co-deletion of 1p and 19q further supports the diagnosis of an oligodendroglioma, IDH-mutant and 1p/19q-codeleted (Section "Combined histological and molecular grading").
- Based on the description, I would grade this tumour as a grade 2, as it does not exhibit features of a more aggressive tumour (Section "Clinicopathological grading" and "Grading within Types").

Please note that this diagnosis is based solely on the attached document and the provided histopathological description, without any prior knowledge or internet research.

Claude Response:

Based on the histopathological description provided, the most likely diagnosis appears to be:

Oligodendroglioma, WHO grade 2

Here's the reasoning behind this diagnosis:
1. Glial tumor: The description mentions a "moderately dense glial tumour," indicating a tumor of glial origin.
2. Mixed cellular components:
   - Oligodendroglial component: "small areas with a rounded tumour cell morphology and a loose cytoplasm" and "occasional perinuclear halos" are characteristic of oligodendroglioma.
   - Astrocytic component: "peculiar astrocytic phenotype with frequent Rosenthal-like inclusions in astrocytes"
3. IDH-1 mutation: Both the oligodendroglial and astrocytic components are positive for mutant IDH-1, which is typical for lower-grade gliomas.
4. 1p/19q co-deletion: This is a defining molecular feature of oligodendrogliomas.
5. Infiltrative nature: The tumor is described as "loosely infiltrated," which is consistent with a diffuse glioma.

The presence of both IDH-1 mutation and 1p/19q co-deletion strongly supports the diagnosis of oligodendroglioma. The lack of mention of significant mitotic activity, microvascular proliferation, or necrosis suggests a lower-grade tumor (WHO grade 2) rather than an anaplastic oligodendroglioma (WHO grade 3).

The astrocytic component with Rosenthal fibers that doesn't express mutant IDH-1 might represent reactive changes in the surrounding brain tissue rather than a true tumor component.

It's worth noting that the final diagnosis should always be made by a qualified pathologist, taking into account all clinical, radiological, and pathological findings.

Claude RAG response:

Based on the histopathological description and molecular findings provided, and referring solely to the WHO diagnostic guidelines document, I believe the diagnosis is:

Oligodendroglioma, IDH-mutant and 1p/19q-codeleted, CNS WHO grade 2

Here's my reasoning, with references to the document:

1. IDH mutation: The tumor shows positive immunostaining for mutant IDH-1 in both oligodendroglial and astrocytic components. This is a key diagnostic feature of IDH-mutant gliomas (Reference: "Diagnostic molecular pathology" section under "Oligodendroglioma, IDH-mutant and 1p/19q-codeleted").
2. 1p/19q codeletion: Molecular pathology revealed co-deletion of 1p and 19q. This is the defining molecular feature of oligodendroglioma (Reference: "Definition" section under "Oligodendroglioma, IDH-mutant and 1p/19q-codeleted").
3. Histological features: The description mentions "small areas with a rounded tumour cell morphology and a loose cytoplasm" and "occasional perinuclear halos," which are consistent with oligodendroglial morphology (Reference: "Histopathology" section under "Oligodendroglioma, IDH-mutant and 1p/19q-codeleted").
4. Mixed morphology: The presence of both oligodendroglial and astrocytic components is mentioned. The document states: "Thus, irrespective of oligodendroglial, oligoastrocytic, astrocytic, or ambiguous features on histology, detection of combined IDH mutation and 1p/19q codeletion indicates an IDH-mutant and 1p/19q-codeleted oligodendroglioma" (Reference: "Histopathology" section under "Oligodendroglioma, IDH-mutant and 1p/19q-codeleted").
5. Grade: The description does not mention features of anaplasia such as significant mitotic activity, microvascular proliferation, or necrosis. Therefore, it's more consistent with a grade 2 tumor (Reference: "Grading" section under "Oligodendroglioma, IDH-mutant and 1p/19q-codeleted").

The presence of Rosenthal-like inclusions is somewhat atypical for oligodendroglioma, but the molecular features (IDH mutation and 1p/19q codeletion) are definitive for this diagnosis according to the current WHO classification.

ChatGPT Response:

Based on the provided histopathological description, the diagnosis is likely to be ****oligoastrocytoma****.

Key points supporting this diagnosis:

1. **Mixed Morphology**: The tumour exhibits both oligodendroglial and astrocytic features. The oligodendroglial component is described with small areas of rounded tumour cells, loose cytoplasm, fine dotted chromatin, and occasional perinuclear halos. The astrocytic component displays Rosenthal-like inclusions in astrocytes.

2. **IDH-1 Immunostaining**: Both the oligodendroglial and astrocytic components are positive for mutant IDH-1, suggesting a common origin and consistent with a diagnosis of a mixed glioma.
3. **1p/19q Co-deletion**: This genetic alteration is characteristic of oligodendroglial tumours, supporting the presence of an oligodendroglial component within the mixed glioma.

Grading: The description does not provide specific details on mitotic activity, necrosis, or microvascular proliferation, which are critical for grading. However, based on the described features, this tumour could be either a grade II (low-grade) or grade III (anaplastic) oligoastrocytoma. Further evaluation of these histological features would be necessary to determine the exact grade.

ChatGPT RAG response:

Based on the histopathological description and immunostaining results provided, the diagnosis appears to be **Oligodendroglioma, IDH-mutant and 1p/19q-codeleted, CNS WHO grade 2 or 3**. Here's how this diagnosis is determined using the WHO guidelines:

Diagnostic Features:

1. **Moderately Dense Glial Tumour**:
   - Indicates a glioma, a type of tumour that originates from glial cells in the central nervous system (CNS).
2. **Small Areas with Rounded Tumour Cell Morphology and Loose Cytoplasm, Nuclei with Fine Dotted Chromatin**:
   - This description matches the classic oligodendroglioma cells which have uniformly round nuclei with a fine chromatin pattern and clear cytoplasm creating a "fried-egg" appearance.
   - Reference: WHO Classification of Tumours, Oligodendroglioma Histopathology Section (Page 3)【3†source】.
3. **Occasional Perinuclear Halos**:
   - Perinuclear halos are characteristic of oligodendrogliomas.
   - Reference: WHO Classification of Tumours, Oligodendroglioma Histopathology Section (Page 3)【3†source】.
4. **Peculiar Astrocytic Phenotype with Rosenthal-like Inclusions**:
   - Rosenthal fibres are often associated with pilocytic astrocytomas but can be present in other types of gliomas, though they are not typically dominant in oligodendrogliomas.
5. **Immunostaining for Mutant IDH-1**:
   - Both the small oligodendroglial component and the astrocytic component of the tumour are positive for mutant IDH-1, indicating an IDH mutation.
   - This is consistent with oligodendroglioma, which requires IDH mutation for diagnosis.
   - Reference: WHO Classification of Tumours, Diagnostic Molecular Pathology (Page 3)【3†source】.
6. **Molecular Pathology Revealed Co-deletion of 1p and 19q**:
   - The presence of 1p/19q co-deletion is a defining feature of oligodendroglioma.
   - Reference: WHO Classification of Tumours, Oligodendroglioma Definition (Page 3)【3†source】.

Grading:
- The histopathological features such as the lack of significant mitotic activity, microvascular proliferation, or necrosis typically indicate a lower grade (CNS WHO grade 2).
- If there is evidence of increased mitotic activity or other features indicative of a more aggressive tumour, it may be CNS WHO grade 3.
- Reference: WHO Classification of Tumours, Oligodendroglioma Grading (Page 3)【3†source】.

Final Diagnosis:
The diagnosis is likely **Oligodendroglioma, IDH-mutant and 1p/19q-codeleted, CNS WHO grade 2 or 3**. The exact grade would depend on additional details regarding mitotic activity and other markers of malignancy not provided in the description.

If you need a precise grade, please provide additional details such as mitotic count, presence of microvascular proliferation, or necrosis.

O3: N17 272 - O2+mol

Multiple blocks from very large resection specimens. The majority of the specimen shows CNS tissue with variable gliosis and some white matter degeneration. Several of these sections are infiltrated by a fairly monomorphic, highly cellular glial tumour. The tumour cells have a monotonous appearance with round nuclei, and a thin rim of perinuclear clearing. There is a rich tumour vasculature with tumour vessels showing plump endothelium, that is not multilayered. Very rare mitotic figures are present but there is no necrosis. Microcystic changes are frequently seen in multiple areas of the tumour. Microcalcifications are noted in a significant proportion of the tumour.

Immunostaining for IDH1 (R132H) is strongly positive in the tumour cells. ATRX immunostaining shows retention of expression in all tumour cell nuclei. The Ki-67 labelling index of this tumour is very low, not exceeding 1%. Molecular testing confirmed that this tumour is IDH1 mutant and 1p19q co-deleted.

What do you think the diagnosis would be?

GT: Oligodendroglioma, IDH-mutant and 1p/19q-codeleted, CNS WHO grade 2

Llama Response:

Based on the histopathological description, I believe the diagnosis is **Oligodendroglioma, WHO Grade II**.

Here's why:

- The monomorphic, highly cellular glial tumour with round nuclei and perinuclear clearing suggests an oligodendroglial tumour.
- The presence of microcystic changes and microcalcifications supports this diagnosis.
- The lack of necrosis, mitotic activity, and endothelial multilayering suggests a low-grade tumour.
- The strong positivity for IDH1 (R132H) and retention of ATRX expression are consistent with oligodendroglioma.
- The 1p19q co-deletion and IDH1 mutation further support the diagnosis of oligodendroglioma.
- The low Ki-67 labelling index (<1%) is also consistent with a low-grade tumour.

Given the lack of high-grade features, I would classify this tumour as a WHO Grade II oligodendroglioma.

Llama RAG:

Based on the histopathological description and the results of immunostaining and molecular testing, I believe the diagnosis is **Oligodendroglioma, IDH-mutant and 1p/19q-codeleted, Grade 2.**

Here's the reasoning:

1. The description of the tumour cells as "monomorphic" and "highly cellular" with "round nuclei" and "thin rim of perinuclear clearing" suggests an oligodendroglial morphology (Introduction to CNS Tumours, page 2).

2. The presence of microcystic changes and microcalcifications is consistent with oligodendroglioma (CNS Tumour Nomenclature, page 3).
3. The strong positivity for IDH1 (R132H) immunostaining and the molecular confirmation of IDH1 mutation support the diagnosis of an IDH-mutant tumour (Gene and Protein Nomenclature, page 4).
4. The retention of ATRX expression in all tumour cell nuclei is also consistent with an oligodendroglioma (CNS Tumour Nomenclature, page 3).
5. The low Ki-67 labelling index (less than 1%) suggests a low-grade tumour (CNS Tumour Grading, page 6).
6. The presence of 1p/19q codeletion, which is a characteristic genetic feature of oligodendroglioma, further supports the diagnosis (Novel Diagnostic Technologies, page 10).

Given the morphology, immunophenotype, and molecular features, I believe the diagnosis is Oligodendroglioma, IDH-mutant and 1p/19q-codeleted, Grade 2.

Claude Response:

Based on the histopathological description and molecular findings provided, the diagnosis is most likely:

Oligodendroglioma, IDH-mutant and 1p19q-codeleted, WHO grade 2

Key features supporting this diagnosis:
1. Histology:
   - Monotonous appearance with round nuclei and perinuclear clearing ("fried egg" appearance typical of oligodendrogliomas)
   - Rich vasculature with plump endothelium (characteristic of oligodendrogliomas)
   - Microcystic changes and microcalcifications (common in oligodendrogliomas)
   - Very low mitotic activity and absence of necrosis (consistent with grade 2)
2. Immunohistochemistry:
   - IDH1 (R132H) mutation positive
   - ATRX retention (distinguishes from astrocytomas)
   - Low Ki-67 labeling index (<1%, consistent with grade 2)
3. Molecular testing:
   - IDH1 mutation confirmed
   - 1p19q codeletion confirmed (diagnostic for oligodendroglioma)

The combination of IDH mutation and 1p19q codeletion is diagnostic for oligodendroglioma according to the WHO classification. The low-grade features (low mitotic activity, absence of necrosis, low Ki-67 index) support a grade 2 designation.

This diagnosis represents a WHO grade 2 tumor, which is considered a lower-grade glioma with a generally better prognosis compared to higher-grade gliomas.

Claude RAG Response:

Based on the histopathological description and molecular findings provided, the diagnosis appears to be:

Oligodendroglioma, IDH-mutant and 1p/19q-codeleted, CNS WHO grade 2

This diagnosis is supported by the following features from the WHO diagnostic guidelines document:

1. Histology:
    - Monotonous appearance with round nuclei and perinuclear clearing ("The tumour cells have a monotonous appearance with round nuclei, and a thin rim of perinuclear clearing.") This is consistent with the classic oligodendroglioma appearance described in the document: "Classic oligodendroglioma cells have uniformly round nuclei that are slightly larger than those of normal oligodendrocytes" and "In formalin-fixed, paraffin-embedded tissue, tumour cells often appear as rounded cells with well-defined cell membranes and clear cytoplasm around the central spherical nucleus."
2. Vascular pattern:
    - Rich tumour vasculature ("There is a rich tumour vasculature with tumour vessels showing plump endothelium") aligns with the description: "Oligodendrogliomas typically show a dense network of branching capillaries resembling chicken wire."
3. Microcalcifications:
    - "Microcalcifications are noted in a significant proportion of the tumour." This is a common feature of oligodendrogliomas, as stated in the document: "Microcalcifications are frequent, found within the tumour itself or in the invaded brain."
4. Microcystic changes:
    - "Microcystic changes are frequently seen in multiple areas of the tumour." This is consistent with the description: "Areas characterized by extracellular mucin deposition and/or microcyst formation are frequent."
5. Molecular findings:
    - IDH1 mutation: "Immunostaining for IDH1 (R132H) is strongly positive in the tumour cells."
    - 1p/19q codeletion: "Molecular testing confirmed that this tumour is IDH1 mutant and 1p19q co-deleted."
    - These molecular findings are essential for the diagnosis of oligodendroglioma, as stated in the document: "Oligodendrogliomas are molecularly defined by IDH1 or IDH2 mutations and 1p/19q codeletion."
6. ATRX status:
    - "ATRX immunostaining shows retention of expression in all tumour cell nuclei." This is consistent with oligodendroglioma, as the document states: "IDH-mutant and 1p/19q-codeleted oligodendrogliomas retain nuclear expression of ATRX."
7. Grade:
    - The tumor is classified as grade 2 due to the following features:
    - Very rare mitotic figures
    - Absence of necrosis
    - Low Ki-67 labeling index (not exceeding 1%)

These features align with the description of CNS WHO grade 2 oligodendroglioma in the document: "Mitotic activity is absent or uncommon in CNS WHO grade 2 tumours" and "The Ki-67 (MIB1) proliferation index is usually low (< 5%) in CNS WHO grade 2 oligodendrogliomas."

In conclusion, the histological features, molecular findings, and low proliferation index are all consistent with the diagnosis of Oligodendroglioma, IDH-mutant and 1p/19q-codeleted, CNS WHO grade 2.

ChatGPT Response:

Based on the histopathological description and the molecular findings provided, the tumour is most consistent with an **Oligodendroglioma, IDH-mutant and 1p/19q co-deleted**. Here's a summary of the key features leading to this diagnosis:

1. **Histopathological Features**:
    - **Monomorphic, highly cellular glial tumour** with round nuclei and a thin rim of perinuclear clearing.
    - **Rich tumour vasculature** with plump endothelium.
    - **Microcystic changes** and **microcalcifications**.
    - **Very rare mitotic figures** and no necrosis.
2. **Immunohistochemical and Molecular Findings**:
    - **Strongly positive IDH1 (R132H) staining**, indicating an IDH1 mutation.
    - **Retention of ATRX expression**.
    - **Very low Ki-67 labelling index** (not exceeding 1%).
    - **Molecular testing confirming IDH1 mutation and 1p/19q co-deletion**.

These features align with the characteristics of oligodendroglioma, which typically shows a combination of IDH mutation and 1p/19q co-deletion. The absence of necrosis and a low mitotic index suggest a lower grade, making this likely a **Grade II oligodendroglioma** according to the World Health Organization (WHO) classification of central nervous system tumours.

ChatGPT RAG response:

Based on the provided histopathological description and immunohistochemical findings, the tumour can be diagnosed as **Oligodendroglioma, IDH-mutant and 1p/19q-codeleted, CNS WHO grade 2**.

Here is the rationale for the diagnosis:
1. **Histopathology**: The description includes features typical of oligodendroglioma:
    - Monomorphic, highly cellular glial tumour with round nuclei.
    - Thin rim of perinuclear clearing (creating a honeycomb or fried-egg appearance).
    - Rich tumour vasculature without multilayered endothelium.
    - Microcystic changes and microcalcifications, which are common in oligodendrogliomas.
2. **Immunohistochemistry**:
    - Strong positivity for IDH1 (R132H), indicating an IDH1 mutation.
    - Retention of ATRX expression, which is consistent with oligodendroglioma as they typically retain ATRX expression unlike astrocytomas which often show loss of ATRX.
3. **Molecular Testing**:
    - Confirmation of IDH1 mutation and 1p/19q co-deletion, which molecularly defines oligodendroglioma.
4. **Low Proliferation Index**:

- The Ki-67 labelling index is very low (not exceeding 1%), suggesting a lower-grade tumour.

The provided findings fit well with **Oligodendroglioma, IDH-mutant and 1p/19q-codeleted, CNS WHO grade 2**, as described in the WHO classification:

- **Histological Features**: Classic oligodendroglioma cells with perinuclear clearing, microcalcifications, and microcystic changes.
- **Immunophenotype**: Positive IDH1 R132H mutation, retained ATRX expression.
- **Molecular Profile**: IDH1 mutation and 1p/19q co-deletion confirm the diagnosis.

Reference: WHO Classification of Tumours, Central Nervous System Tumours, 5th Edition【3†source】.

O4: NH19 979 - O2+mol

Histology shows multiple small CUSA specimens of a moderately cellular tumour. The tumour is composed of monomorphic cells with round nuclei, dark chromatin and scanty cytoplasm, however perinuclear haloes are not noted. The tumour cells are arranged in patternless sheets without clear rosette or perivascular pseudorosette formations. There is microcalcification in the stroma. Branching capillary networks are identified in places, but microvascular proliferation is not observed. Neither necrosis nor mitotic figures are readily seen.
Immunostaining for mutant IDH1 (R132H) is negative and ATRX shows retained nuclear expression, although this is technically weak. Further testing of both has been requested. The Ki67 proliferation fraction is only mildly increased, estimated at 3%.

Molecular pathology results:
IDH1/2 sequencing: IDH2 mutation
1p19q: co-deleted

What do you think the diagnosis would be?

GT: Oligodendroglioma, IDH-mutant and 1p/19q-codeleted, CNS WHO grade 2

Llama Response:

Based on the histopathological description and molecular pathology results, I would diagnose this tumour as an **Oligodendroglioma, grade II**.

The key features that support this diagnosis are:

- Monomorphic cells with round nuclei and scanty cytoplasm, arranged in patternless sheets
- Absence of perinuclear haloes and rosette or perivascular pseudorosette formations
- Presence of microcalcification in the stroma
- Branching capillary networks and lack of microvascular proliferation
- Low Ki67 proliferation fraction (3%)
- IDH2 mutation, which is a common genetic alteration in oligodendrogliomas
- Co-deletion of 1p19q, which is a hallmark of oligodendrogliomas

The lack of necrosis, mitotic figures, and microvascular proliferation suggests a low-grade tumour, which is consistent with a grade II oligodendroglioma.

Llama RAG response:

Based on the histopathological description and molecular pathology results, I think the diagnosis would be **Oligodendroglioma, IDH-mutant and 1p/19q-codeleted, Grade 2**.

Here's my reasoning:

1. The histological description of a moderately cellular tumour with monomorphic cells, round nuclei, and scanty cytoplasm, arranged in patternless sheets, is consistent with an oligodendroglial tumour.

2. The lack of perinuclear haloes and the presence of microcalcification in the stroma also support an oligodendroglial tumour diagnosis.
3. The negative IDH1 (R132H) immunostain and retained ATRX nuclear expression (although technically weak) suggest that the tumour is an IDH-mutant oligodendroglioma.
4. The IDH2 mutation detected by sequencing further supports this diagnosis.
5. The 1p/19q codeletion is a characteristic genetic feature of oligodendrogliomas.
6. The low Ki67 proliferation fraction (3%) is consistent with a Grade 2 tumour.

References:

- CNS Tumour Grading: The grading of CNS tumours has moved closer to how grading is done for non-CNS neoplasms, but it has retained some key aspects of traditional CNS tumour grading. (Page 5)
- Oligodendroglioma: IDH-mutant and 1p/19q-codeleted oligodendrogliomas are classified as Grade 2. (Not explicitly stated in the document, but implied by the discussion of grading and the characteristics of oligodendrogliomas)
- Molecular Parameters: IDH mutations and 1p/19q codeletion are key molecular parameters in the classification of oligodendrogliomas. (Pages 10-11)

Claude Response:

Based on the histopathological description and molecular pathology results provided, the diagnosis is most likely:

Oligodendroglioma, IDH-mutant and 1p/19q-codeleted, WHO grade 2

Reasoning:
1. Histology:
   - Moderately cellular tumor with monomorphic cells
   - Round nuclei with dark chromatin
   - Patternless sheets arrangement
   - Presence of microcalcification
   - Branching capillary networks
   - Absence of significant mitotic activity, necrosis, and microvascular proliferation
2. Immunohistochemistry:
   - Low Ki67 proliferation fraction (3%)
   - Negative for IDH1 R132H mutation (but this doesn't rule out other IDH mutations)
   - Retained ATRX expression (though weak)
3. Molecular pathology:
   - IDH2 mutation present
   - 1p/19q co-deletion present

The combination of an IDH mutation (in this case IDH2) and 1p/19q co-deletion is diagnostic for oligodendroglioma according to the WHO classification. The absence of significant mitotic activity,

microvascular proliferation, and necrosis, along with the low Ki67 index, supports a grade 2 designation rather than a grade 3 anaplastic oligodendroglioma.

The histological features, particularly the monomorphic cells with round nuclei and microcalcification, are also consistent with oligodendroglioma, although classic "fried egg" appearance with perinuclear halos is not noted in this case.

Claude RAG response:

Based on the histopathological description and molecular pathology results provided, the diagnosis appears to be:

Oligodendroglioma, IDH-mutant and 1p/19q-codeleted, CNS WHO grade 2

Here's the reasoning based on the WHO diagnostic guidelines document:

1. Histology:
   - The tumor shows features consistent with oligodendroglioma, including monomorphic cells with round nuclei and dark chromatin, although perinuclear haloes (a common feature) are not noted.
   - Microcalcification and branching capillary networks are present, which are typical of oligodendroglioma.
   - The absence of microvascular proliferation, necrosis, and readily visible mitotic figures supports a lower grade.
2. Molecular pathology:
   - IDH2 mutation is present, which fulfills the IDH-mutant criterion.
   - 1p/19q co-deletion is present, which is a defining feature of oligodendroglioma.
3. Immunohistochemistry:
   - Retained nuclear ATRX expression is consistent with oligodendroglioma (loss of ATRX is more typical of astrocytoma).
   - The low Ki67 proliferation fraction (3%) supports a lower grade designation.

According to the document, the essential diagnostic criteria for Oligodendroglioma, IDH-mutant and 1p/19q-codeleted include:
"This tumour must have IDH1 codon 132 or IDH2 codon 172 missense mutation and combined whole-arm deletions of 1p and 19q." (Reference: "Essential and desirable diagnostic criteria" section under "Oligodendroglioma, IDH-mutant and 1p/19q-codeleted")

The grading as CNS WHO grade 2 is supported by the absence of features indicating higher grade, such as:
"CNS WHO grade 2 tumours are characterised by a diffusely infiltrative astrocytic glioma with an IDH1 or IDH2 mutation that is well differentiated and lacks histological features of anaplasia. Mitotic activity is not detected or very low." (Reference: "Grading" section under "Astrocytoma, IDH-mutant")

While this refers to astrocytoma, similar principles apply to oligodendroglioma grading.

It's worth noting that further testing of IDH1 and ATRX has been requested, which could potentially modify this diagnosis if unexpected results are obtained. However, based on the information provided,

Oligodendroglioma, IDH-mutant and 1p/19q-codeleted, CNS WHO grade 2 appears to be the most appropriate diagnosis.

ChatGPT Response:

The histopathological and molecular features described are indicative of an oligodendroglioma. Here's how the features correlate with this diagnosis:

1. **Histopathology**:
   - **Moderately cellular tumour**: Oligodendrogliomas typically have moderate cellularity.
   - **Monomorphic cells with round nuclei and dark chromatin**: This is a common feature of oligodendrogliomas.
   - **Scanty cytoplasm**: Also typical of oligodendrogliomas.
   - **Patternless sheets of cells**: Oligodendrogliomas can have a patternless appearance.
   - **Microcalcifications**: A characteristic feature often seen in oligodendrogliomas.
   - **Branching capillary networks**: These "chicken-wire" vascular patterns are a classic finding in oligodendrogliomas.
   - **Lack of necrosis and mitotic figures**: These findings suggest a lower-grade tumour.
2. **Immunohistochemistry**:
   - **Mutant IDH1 (R132H) negative**: IDH2 mutations can be seen in oligodendrogliomas.
   - **Retained ATRX expression**: ATRX is typically retained in oligodendrogliomas.
   - **Ki67 proliferation fraction mildly increased at 3%**: This suggests a low proliferative index, consistent with a lower-grade tumour.
3. **Molecular pathology**:
   - **IDH2 mutation**: IDH mutations (either IDH1 or IDH2) are common in oligodendrogliomas.
   - **1p/19q co-deletion**: This is a defining feature of oligodendrogliomas and is used to distinguish them from other gliomas.

Given the histological and molecular findings, the tumour is consistent with an **oligodendroglioma, IDH-mutant and 1p/19q co-deleted**. The absence of significant mitotic activity, necrosis, and a low Ki67 index suggests a lower-grade tumour, likely a **Grade II oligodendroglioma** according to the World Health Organization (WHO) classification of central nervous system tumours.

ChatGPT RAG response:

Based on the histopathological and molecular findings provided, the diagnosis can be reached using the guidelines in the WHO Classification of Tumours for Central Nervous System Tumours.

Histopathological and Molecular Features:
1. **Histology**:
   - Moderately cellular tumour
   - Monomorphic cells with round nuclei, dark chromatin, and scanty cytoplasm
   - Patternless sheets without clear rosette or perivascular pseudorosette formations
   - Microcalcification in the stroma
   - Branching capillary networks identified, but no microvascular proliferation

- Absence of necrosis and mitotic figures
- Ki67 proliferation fraction estimated at 3%

2. **Immunostaining**:
- Mutant IDH1 (R132H) negative
- ATRX shows retained nuclear expression (weak but technically retained)

3. **Molecular Pathology**:
- IDH1/2 sequencing: IDH2 mutation present
- 1p/19q co-deletion present

Diagnosis:
Based on the above information, the diagnosis is consistent with **Oligodendroglioma, IDH-mutant, and 1p/19q-codeleted**.

Grading:
Given the absence of mitotic figures, necrosis, and microvascular proliferation, along with a low Ki67 proliferation index (3%), this tumour would be classified as a **CNS WHO grade 2 oligodendroglioma**.

References:
- The presence of IDH2 mutation and 1p/19q co-deletion strongly supports the diagnosis of oligodendroglioma (WHO Classification of Tumours, section on Oligodendroglioma, IDH-mutant and 1p/19q-codeleted).
- The histopathological description fits well with the classic features of oligodendroglioma, including the monomorphic cells and branching capillary networks (WHO Classification of Tumours, section on Histopathology of Oligodendroglioma).
- The retained ATRX expression (though weak) and negative IDH1 immunostaining align with oligodendroglioma's molecular profile when 1p/19q co-deletion is present (WHO Classification of Tumours, Diagnostic molecular pathology section).

Thus, the most appropriate diagnosis and grade for the tumour based on the provided information and WHO guidelines is **Oligodendroglioma, IDH-mutant, and 1p/19q-codeleted, CNS WHO grade 2**.

O5: NH15 24 - O2+FISH

This is a large specimen including surgical aspirate. Identified tumour shows oligodendroglial features. No mitoses, no microvascular proliferation or necrosis are seen. IHC for mutant IDH is positive and ATRX is retained in all tumour cells. FISH confirms 1p/19q co-deletion.

What do you think the diagnosis would be?

GT: Oligodendroglioma, IDH-mutant and 1p/19q-codeleted, CNS WHO grade 2

Llama Response:

Based on the histopathological description, I think the diagnosis is **Oligodendroglioma, WHO Grade II**.

The presence of oligodendroglial features, lack of mitoses, microvascular proliferation, and necrosis, and the positive IHC for mutant IDH and retained ATRX stain, are all consistent with an oligodendroglioma. The FISH confirmation of 1p/19q co-deletion is also a characteristic feature of oligodendrogliomas.

The lack of high-grade features and the retained ATRX stain suggest a low-grade tumor, which is consistent with a WHO Grade II designation.

Llama RAG response:

Based on the provided histopathological description, I believe the diagnosis is **Oligodendroglioma, IDH-mutant, 1p/19q-codeleted, WHO grade 2**.

Here's the reasoning:

- Oligodendroglial features are seen in the tumour, which suggests an oligodendroglioma (Page 5, CNS Tumour Nomenclature).
- The absence of mitoses, microvascular proliferation, and necrosis indicates a lower-grade tumour.
- The positivity for mutant IDH and retention of ATRX in all tumour cells supports an IDH-mutant oligodendroglioma diagnosis (Page 10, Clinicopathological grading).
- The presence of 1p/19q co-deletion, confirmed by FISH, is a characteristic molecular feature of oligodendrogliomas (Page 10, Clinicopathological grading).
- Given the presence of oligodendroglial features, IDH mutation, and 1p/19q co-deletion, the tumour is classified as an oligodendroglioma, IDH-mutant, 1p/19q-codeleted.
- The absence of high-grade features (mitoses, microvascular proliferation, and necrosis) suggests a WHO grade 2 tumour (Page 11, Clinicopathological grading).

Please note that this diagnosis is based solely on the provided histopathological description and the attached WHO classification of tumours document.

Claude Response:

Based on the histopathological description and molecular findings provided, the diagnosis is most likely:

Oligodendroglioma, IDH-mutant and 1p/19q-codeleted, WHO grade 2

Reasoning:

1. Oligodendroglial features: The description mentions that the tumor shows oligodendroglial features, which is characteristic of oligodendrogliomas.
2. Absence of high-grade features: There are no mitoses, microvascular proliferation, or necrosis mentioned, which are typically associated with higher-grade tumors.
3. IDH mutation: The immunohistochemistry (IHC) for mutant IDH is positive, which is a key molecular feature of oligodendrogliomas.
4. ATRX retention: ATRX is retained in all tumor cells, which is consistent with oligodendrogliomas (ATRX loss is more commonly associated with astrocytomas).
5. 1p/19q co-deletion: FISH confirms 1p/19q co-deletion, which is a defining molecular feature of oligodendrogliomas.
6. Grade 2: The absence of mitotic activity, microvascular proliferation, and necrosis suggests a lower-grade tumor. In the absence of any mentioned anaplastic features, this would be classified as a WHO grade 2 tumor.

The combination of oligodendroglial morphology, IDH mutation, and 1p/19q co-deletion, along with the absence of high-grade features, strongly supports the diagnosis of a WHO grade 2 oligodendroglioma according to the current WHO classification of CNS tumors.

Claude RAG response:

Based on the histopathological description provided and using only the information from the attached WHO diagnostic guidelines document, I believe the diagnosis for this tumor is:

Oligodendroglioma, IDH-mutant and 1p/19q-codeleted, CNS WHO grade 2

Here's my reasoning, referencing the relevant sections of the document:

1. Oligodendroglial features: The tumor shows oligodendroglial features, which is consistent with the histopathology of oligodendroglioma as described in the document.
2. IDH mutation: The immunohistochemistry (IHC) for mutant IDH is positive. This is a key diagnostic criterion for oligodendroglioma, as stated in the document: "Oligodendrogliomas are molecularly defined by IDH1 or IDH2 mutations" (Diagnostic molecular pathology section).
3. 1p/19q codeletion: FISH confirms 1p/19q co-deletion, which is another essential criterion for oligodendroglioma diagnosis. The document states: "Diagnosis of oligodendrogliomas requires demonstration of IDH mutation by IDH1 p.R132H immunohistochemistry and/or sequencing of the IDH1 or IDH2 gene, as well as demonstration of 1p/19q codeletion" (Diagnostic molecular pathology section).
4. ATRX retention: The document mentions that "IDH-mutant and 1p/19q-codeleted oligodendrogliomas retain nuclear expression of ATRX" (Immunophenotype section), which is consistent with the description provided.
5. Grading: The absence of mitoses, microvascular proliferation, and necrosis supports a lower grade. The document states for CNS WHO grade 2: "Mitotic activity is not detected or very low.

Microvascular proliferation, necrosis, and homozygous deletions of CDKN2A and/or CDKN2B are absent" (Grading section).

The "Essential and desirable diagnostic criteria" section of the document confirms that this tumor meets the essential criteria for Oligodendroglioma, IDH-mutant and 1p/19q-codeleted: "This tumour must have IDH1 codon 132 or IDH2 codon 172 missense mutation and combined whole-arm deletions of 1p and 19q."

Therefore, based solely on the information provided in the WHO diagnostic guidelines document, the most appropriate diagnosis for this tumor is Oligodendroglioma, IDH-mutant and 1p/19q-codeleted, CNS WHO grade 2.

ChatGPT Response:

Based on the histopathological description provided, the diagnosis is likely ****Oligodendroglioma, IDH-mutant and 1p/19q-codeleted****.

Key points from the description that support this diagnosis:
- **Oligodendroglial features**: Suggests the tumor has characteristics of oligodendroglioma.
- **No mitoses, no microvascular proliferation or necrosis**: Indicates a lower grade tumor, specifically Grade II, as higher grades would show more aggressive features.
- **IHC for mutant IDH is positive**: Confirms the presence of an IDH mutation, which is a typical finding in oligodendrogliomas and astrocytomas.
- **ATRX is retained**: Consistent with oligodendroglioma, as ATRX loss is usually seen in astrocytomas.
- **FISH confirms 1p/19q co-deletion**: This genetic hallmark is definitive for oligodendroglioma.

Considering the absence of mitotic activity, microvascular proliferation, or necrosis, the tumor would be classified as a ****Grade II Oligodendroglioma**** according to the World Health Organization (WHO) classification.

ChatGPT RAG response:

Based on the histopathological description provided and referencing the attached WHO guidelines for CNS tumours, the diagnosis can be determined as follows:

1. **Tumour Features:**
   - **Oligodendroglial features**: Indicates the tumour has characteristics typical of oligodendrogliomas.
   - **No mitoses, no microvascular proliferation, or necrosis**: These features suggest a lower grade tumour.
   - **IHC for mutant IDH is positive**: Indicates the presence of an IDH mutation.
   - **ATRX is retained in all tumour cells**: Retained ATRX expression is typical for oligodendrogliomas (as opposed to IDH-mutant astrocytomas where ATRX is often lost).

- **FISH confirms 1p/19q co-deletion**: The presence of this co-deletion is a defining feature of oligodendrogliomas.

2. **Diagnosis:**
   - **Type**: Given the presence of oligodendroglial features, positive IDH mutation, retained ATRX expression, and 1p/19q co-deletion, the tumour is classified as an **Oligodendroglioma, IDH-mutant and 1p/19q-codeleted**.
   - **Grade**: The absence of mitoses, microvascular proliferation, and necrosis suggests this is a lower grade tumour. Specifically, these features correspond to a **CNS WHO grade 2** oligodendroglioma.

Diagnosis:
Oligodendroglioma, IDH-mutant and 1p/19q-codeleted, CNS WHO grade 2

References:
- WHO Classification of Tumours, Central Nervous System Tumours, 5th Edition:
- **Definition**: "Oligodendroglioma IDH-mutant and 1p/19q-codeleted is a diffusely infiltrating glioma with IDH1 or IDH2 mutation and codeletion of chromosome arms 1p and 19q (CNS WHO grade 2 or 3)."
- **Grading**: "Oligodendrogliomas comprise a continuous spectrum of tumours ranging from well-differentiated slow-growing neoplasms to frankly malignant tumours with rapid growth. In prior editions of the WHO classification of CNS tumours two grades were distinguished: oligodendroglioma CNS WHO grade 2 and oligodendroglioma CNS WHO grade 3. CNS WHO grade retained prognostic significance in patients with IDH-mutant and 1p/19q-codeleted oligodendrogliomas but the criteria for distinction between grades were not well defined. Histological features that have been linked to higher grade are high cellularity, marked cytological atypia, brisk mitotic activity, pathological microvascular proliferation, and necrosis with or without palisading."【3†source】.

O6: NH16 375 - O3+mol

These are fragments of a glial tumour with oligodendroglial morphology. The tumour is mostly moderately cellular consisting of monomorphic cells, which have round to oval nuclei and mild pleomorphism. Perinuclear clearing is prominent. Areas of calcification are identified.
In places, more densely cellular areas composed of pleomorphic cells with hyperchromatic nuclei are noted. In these areas, up to 6 mitotic figures per HPF are noted and atypical mitotic figures are also observed. Frequent apoptotic cells and areas of necrosis are identified. Vessels with atypical endothelium are noted but no true vascular proliferations with multilayered endothelium are identified.
On immunohistochemistry mutant IDH1 (R132H) is positive and ATRX is retained. H3.3 (K27M) is negative. A sample was sent for DNA analysis and revealed IDH1 mutation, 1p19q co-deletion, TERT promoter mutation and histone H3F3 wild-type.

What do you think the diagnosis would be?

GT: Oligodendroglioma, IDH-mutant and 1p/19q-codeleted, CNS WHO grade 3

Llama Response:

What a delightful challenge!

Based on the histopathological description, I would diagnose this tumour as an **Anaplastic Oligodendroglioma, likely Grade III**.

Here's my reasoning:

1. The tumour's oligodendroglial morphology, with round to oval nuclei and perinuclear clearing, points towards an oligodendroglioma.
2. The presence of areas with higher cellularity, pleomorphism, and mitotic activity (up to 6 mitotic figures per HPF) suggests an anaplastic component, which is consistent with a higher-grade oligodendroglioma.
3. The presence of calcification, frequent apoptotic cells, and areas of necrosis further supports the diagnosis of an anaplastic oligodendroglioma.
4. The immunohistochemical profile, with positive staining for mutant IDH1 (R132H) and retained ATRX, is consistent with an oligodendroglioma. The negative staining for H3.3 (K27M) helps to rule out a high-grade glioma with a histone H3 mutation.
5. The molecular genetic findings, including IDH1 mutation, 1p19q co-deletion, and TERT promoter mutation, are characteristic of oligodendrogliomas, particularly anaplastic oligodendrogliomas.

Given the presence of anaplastic features, mitotic activity, and necrosis, I would classify this tumour as an Anaplastic Oligodendroglioma, likely Grade III.

Please let me know if I'm correct or not!

Llama RAG response:

Based on the provided histopathological description and molecular analysis, I believe the diagnosis is an **Oligodendroglioma, IDH-mutant and 1p/19q-codeleted, Grade 3.**

Here's the reasoning:

- The tumour has oligodendroglial morphology, which is consistent with an oligodendroglioma.
- The presence of calcification is a common feature of oligodendrogliomas (WHO Classification of Tumours, 5th edition, page 24).
- The more densely cellular areas with pleomorphic cells, mitotic activity, and necrosis suggest a higher grade tumour.
- The immunohistochemistry results show positivity for mutant IDH1 (R132H), which is a characteristic feature of oligodendrogliomas (WHO Classification of Tumours, 5th edition, page 24).
- The DNA analysis reveals an IDH1 mutation, 1p/19q co-deletion, and TERT promoter mutation, which are all consistent with an oligodendroglioma (WHO Classification of Tumours, 5th edition, page 24).
- The retained ATRX expression and wild-type H3.3 (K27M) further support the diagnosis (WHO Classification of Tumours, 5th edition, page 24).
- The grade is determined by the presence of mitotic activity, necrosis, and the overall histological features, which are consistent with a Grade 3 oligodendroglioma (WHO Classification of Tumours, 5th edition, page 25).

References:
* WHO Classification of Tumours, 5th edition, pages 24-25.

Please note that this diagnosis is based solely on the provided information and the attached WHO Classification of Tumours document.

Claude Response:

Based on the histopathological description and molecular findings provided, the diagnosis is most likely:

Anaplastic Oligodendroglioma, WHO grade 3

Reasoning:

1. Oligodendroglial morphology: The description mentions "oligodendroglial morphology" and "perinuclear clearing," which is characteristic of oligodendrogliomas.
2. Mixed cellularity: There are areas of moderate cellularity and areas of higher cellularity with more aggressive features.
3. Anaplastic features: The presence of increased mitotic activity (up to 6 per HPF), atypical mitoses, frequent apoptosis, and areas of necrosis are indicative of anaplastic (grade 3) features.
4. Molecular findings:
    - IDH1 mutation (both immunohistochemistry and DNA analysis)
    - 1p19q co-deletion
    - TERT promoter mutation
    - These molecular alterations are characteristic of oligodendrogliomas.

5. Other findings:
- ATRX retention is consistent with oligodendroglioma (ATRX loss is more typical in astrocytomas)
- H3.3 (K27M) negativity rules out diffuse midline glioma
- Histone H3F3 wild-type further supports the oligodendroglioma diagnosis

The combination of oligodendroglial morphology, anaplastic features, and the specific molecular profile (especially IDH mutation and 1p19q co-deletion) is diagnostic for an Anaplastic Oligodendroglioma, WHO grade 3.

Claude RAG response:

Based on the histopathological description and molecular findings provided, and using only the information from the attached WHO diagnostic guidelines document, the diagnosis appears to be:

Oligodendroglioma, IDH-mutant and 1p/19q-codeleted, CNS WHO grade 3

Here's the reasoning behind this diagnosis, with references to the relevant sections of the document:

1. Histology:
   - The tumor shows oligodendroglial morphology with perinuclear clearing, which is characteristic of oligodendrogliomas. The document states: "Classic oligodendroglioma cells have uniformly round nuclei that are slightly larger than those of normal oligodendrocytes and show an increase in chromatin density or a delicate salt-and-pepper pattern." (Histopathology section of Oligodendroglioma, IDH-mutant and 1p/19q-codeleted)
2. Molecular findings:
   - IDH1 mutation (both immunohistochemistry and DNA analysis)
   - 1p/19q co-deletion
   - TERT promoter mutation
   - These findings are consistent with the essential diagnostic criteria for oligodendroglioma: "Oligodendroglioma, IDH-mutant and 1p/19q-codeleted must display features of a diffusely infiltrating glioma. In addition, this tumour must have IDH1 codon 132 or IDH2 codon 172 missense mutation and combined whole-arm deletions of 1p and 19q." (Essential and desirable diagnostic criteria section)
3. Grade: The tumor shows features of anaplasia, including:
   - Increased cellularity in some areas
   - Pleomorphism
   - High mitotic activity (up to 6 mitoses per HPF)
   - Atypical mitoses
   - Apoptosis
   - Necrosis
   - These features are consistent with CNS WHO grade 3, as described in the document: "CNS WHO grade 3 astrocytomas are characterised by a diffusely infiltrative astrocytic glioma with an IDH1 or IDH2 mutation that exhibits focal or dispersed anaplasia and displays significant mitotic activity." (Grading subsection of Astrocytoma, IDH-mutant)

   While this description is for astrocytomas, the grading principles are similar for oligodendrogliomas.
4. Exclusion of other entities:

- ATRX is retained, which is consistent with oligodendroglioma and helps exclude astrocytoma: "Most IDH-mutant astrocytomas show even more widespread (> 50%) p53 expression." (Diagnostic molecular pathology subsection of Astrocytoma, IDH-mutant)
- H3.3 (K27M) is negative, excluding diffuse midline glioma, H3 K27–altered.

In conclusion, the combination of oligodendroglial morphology, IDH mutation, 1p/19q co-deletion, and features of anaplasia support the diagnosis of Oligodendroglioma, IDH-mutant and 1p/19q-codeleted, CNS WHO grade 3.

ChatGPT Response:

Based on the histopathological description provided, the most likely diagnosis is an ****Oligodendroglioma, WHO Grade III (anaplastic oligodendroglioma)****. Here's the rationale:

Morphology and Histopathology
- **Oligodendroglial morphology**: The tumour shows characteristics typical of oligodendrogliomas, including monomorphic cells with round to oval nuclei and mild pleomorphism.
- **Perinuclear clearing**: A common feature in oligodendrogliomas, giving a "fried egg" appearance.
- **Calcification**: Another characteristic feature often seen in oligodendrogliomas.
- **Densely cellular areas with pleomorphic cells and hyperchromatic nuclei**: Suggests a higher grade tumour.
- **Mitotic figures**: The presence of up to 6 mitotic figures per high power field (HPF) and atypical mitotic figures indicate increased proliferative activity, consistent with a higher grade (grade III).
- **Apoptotic cells and areas of necrosis**: Necrosis is a feature of higher-grade tumours.
- **Atypical endothelium** without true vascular proliferation: Vascular abnormalities are common in higher-grade gliomas.

Immunohistochemistry and Molecular Features
- **Mutant IDH1 (R132H) positive**: Suggests an IDH-mutant glioma, commonly seen in oligodendrogliomas.
- **ATRX retained**: Retention of ATRX is often seen in oligodendrogliomas, whereas loss is more common in astrocytomas.
- **H3.3 (K27M) negative**: Excludes diffuse midline gliomas.
- **1p19q co-deletion**: A hallmark of oligodendrogliomas.
- **TERT promoter mutation**: Frequently found in oligodendrogliomas.
- **Histone H3F3 wild-type**: Confirms the exclusion of H3 K27M mutant diffuse midline glioma.

Conclusion
- The combination of histopathological features and molecular findings, including the IDH1 mutation, 1p19q co-deletion, and TERT promoter mutation, are diagnostic of an **Oligodendroglioma**. The presence of high mitotic activity, pleomorphism, apoptotic cells, and necrosis suggests a higher-grade tumour, making it a **Grade III (anaplastic oligodendroglioma)**.

ChatGPT RAG response:

Based on the provided histopathological description and the information from the WHO guidelines, the diagnosis for the described tumor can be determined as follows:

1. **Histopathological and Immunohistochemical Features:**

- **Oligodendroglial morphology**: The tumor consists of cells with round to oval nuclei and perinuclear clearing, which is characteristic of oligodendrogliomas.
- **Calcification**: Commonly seen in oligodendrogliomas.
- **High cellularity and pleomorphism**: More densely cellular areas with pleomorphic cells and hyperchromatic nuclei.
- **Mitotic figures**: Up to 6 mitotic figures per HPF and atypical mitotic figures indicate a higher grade tumor.
- **Necrosis and apoptosis**: Areas of necrosis and frequent apoptotic cells are identified.
- **Vascular features**: Vessels with atypical endothelium but no true vascular proliferations.
- **Immunohistochemistry**: Positive for IDH1 (R132H) mutation and retained ATRX expression. H3.3 (K27M) is negative.
- **DNA analysis**: Revealed IDH1 mutation, 1p/19q co-deletion, TERT promoter mutation, and histone H3F3 wild-type.

2. **Diagnostic Criteria (WHO 2021 Classification):**
   - The presence of an **IDH1 mutation** and **1p/19q co-deletion** confirms the diagnosis of an oligodendroglioma.
   - **TERT promoter mutation** and **retained ATRX expression** are consistent with oligodendroglioma.
   - The histological features of high mitotic activity, necrosis, and hypercellularity indicate a higher grade tumor.

Based on these features, the diagnosis is **Oligodendroglioma, IDH-mutant and 1p/19q-codeleted, CNS WHO grade 3**. This conclusion is supported by the following sections of the WHO guidelines:

- **Oligodendroglioma IDH-mutant and 1p/19q-codeleted**: Defined as a diffusely infiltrating glioma with IDH1 or IDH2 mutation and 1p/19q codeletion (CNS WHO grade 2 or 3)【3†source】.
- **Grading**: CNS WHO grade 3 oligodendrogliomas usually show high cellularity, marked cytological atypia, brisk mitotic activity, and necrosis【3†source】.

Thus, the final diagnosis is:

****Oligodendroglioma, IDH-mutant and 1p/19q-codeleted, CNS WHO grade 3****.

O7: NH16 921 - O3+FISH

Multiple large fragments of CNS tissue showing a monomorphic, hypercellular glial tumour. Tumour cells have monomorphic nuclei with fine, granular chromatin and perinuclear halos. Mitotic figures are commonly seen. There are several areas of geographic necrosis and frequent microvascular proliferations in the tumour.
Immunostaining for mutant IDH1 is positive. ATRX shows retention of staining in all tumour nuclei. The Ki-67 immunolabelling for proliferation index is estimated at 20%. 1p19q is co-deleted on FISH.

What do you think the diagnosis would be?

GT: Oligodendroglioma, IDH-mutant and 1p/19q-codeleted, CNS WHO grade 3

Llama Response:

A great histopathological description!

Based on the features you've described, I think the diagnosis is:

****Oligodendroglioma, WHO Grade III (Anaplastic Oligodendroglioma)****

Here's why:

- The monomorphic, hypercellular glial tumour with perinuclear halos suggests an oligodendroglial neoplasm.
- The presence of mitotic figures, geographic necrosis, and microvascular proliferations indicates a high-grade tumour, which is consistent with an anaplastic oligodendroglioma (WHO Grade III).
- The positive immunostaining for mutant IDH1 is supportive of an oligodendroglioma, as IDH1 mutations are common in this type of tumour.
- The retention of ATRX staining in all tumour nuclei is also consistent with an oligodendroglioma.
- The Ki-67 proliferation index of 20% is moderately high, which is consistent with a high-grade tumour.
- The co-deletion of 1p19q on FISH is a characteristic genetic feature of oligodendrogliomas, particularly those with IDH1 mutations.

Therefore, based on the histopathological and immunohistochemical features, I believe the diagnosis is an Anaplastic Oligodendroglioma (WHO Grade III).

Llama RAG response:

Based on the provided histopathological description, I believe the diagnosis would be **Oligodendroglioma, IDH-mutant and 1p/19q-codeleted, Grade 3**.

Here's the reasoning and references to the attached document:

- The tumour's monomorphic, hypercellular glial morphology and geographic necrosis suggest a high-grade glioma. (p. 8, CNS Tumour Grading)

- The presence of perinuclear halos and fine, granular chromatin in the tumour cells is consistent with an oligodendroglioma. (p. 10, CNS Tumour Nomenclature)
- The positive immunostaining for mutant IDH1 suggests an IDH-mutant tumour. (p. 11, Gene and Protein Nomenclature)
- The retention of ATRX staining in all tumour nuclei supports an oligodendroglioma diagnosis. (p. 12, CNS Tumour Grading)
- The Ki-67 proliferation index of 20% is consistent with a high-grade glioma. (p. 13, Clinicopathological Grading)
- The 1p19q codeletion on FISH is a characteristic feature of oligodendrogliomas. (p. 14, Molecular Parameters)
- Given the presence of mitotic figures, geographic necrosis, and microvascular proliferations, the tumour is likely Grade 3. (p. 15, Grading within Types)

Based on the integrated diagnosis, I conclude that the tumour is an **Oligodendroglioma, IDH-mutant and 1p/19q-codeleted, Grade 3**.

Claude Response:

Based on the histopathological description and immunohistochemical findings provided, the most likely diagnosis is:

Oligodendroglioma, WHO grade 3 (Anaplastic Oligodendroglioma)

Here's the rationale:
1. The description indicates a glial tumor with monomorphic cells, perinuclear halos, and fine chromatin, which are classic features of oligodendrogliomas.
2. The presence of frequent mitotic figures, geographic necrosis, and microvascular proliferation suggest a high-grade (anaplastic) tumor.
3. Positive IDH1 mutation is common in oligodendrogliomas.
4. ATRX retention is consistent with oligodendroglioma (ATRX loss is more typical of astrocytomas).
5. The high Ki-67 proliferation index (20%) supports a high-grade diagnosis.
6. The presence of 1p19q co-deletion on FISH is diagnostic for oligodendroglioma.
7. The combination of IDH mutation and 1p19q co-deletion is characteristic of oligodendrogliomas according to the 2016 WHO classification of CNS tumors.

The presence of necrosis and microvascular proliferation, along with the high Ki-67 index, supports a grade 3 (anaplastic) classification rather than a grade 2.

Therefore, the most likely diagnosis is an Anaplastic Oligodendroglioma, WHO grade 3.

Claude RAG response:

Based on the histopathological description and immunohistochemical findings provided, and using only the information from the attached WHO diagnostic guidelines document, I believe the diagnosis for this tumor is:

Oligodendroglioma, IDH-mutant and 1p/19q-codeleted, CNS WHO grade 3

Here's my reasoning, with references to the attached document:

1. Histology:

- The description of "monomorphic, hypercellular glial tumour" with "monomorphic nuclei with fine, granular chromatin and perinuclear halos" is consistent with the histological features of oligodendroglioma described in the document (see "Histopathology" section under "Oligodendroglioma, IDH-mutant and 1p/19q-codeleted").

2. Molecular features:

- Positive IDH1 mutation: "Oligodendrogliomas are molecularly defined by IDH1 or IDH2 mutations" (see "Diagnostic molecular pathology" section).
- 1p/19q co-deletion: "Diagnosis of oligodendrogliomas requires demonstration of IDH mutation by IDH1 p.R132H immunohistochemistry and/or sequencing of the IDH1 or IDH2 gene, as well as demonstration of 1p/19q codeletion" (see "Diagnostic molecular pathology" section).
- ATRX retention: "IDH-mutant and 1p/19q-codeleted oligodendrogliomas retain nuclear expression of ATRX" (see "Immunophenotype" section).

3. Grade: The tumor is classified as CNS WHO grade 3 due to the following features:

- "Common mitotic figures" (see "Grading" section)
- "Geographic necrosis" (see "Grading" section)
- "Frequent microvascular proliferations" (see "Grading" section)
- High Ki-67 proliferation index (20%): "In CNS WHO grade 3 tumours, the Ki-67 proliferation index is usually in the range of 4–10%, but it can overlap with values for CNS WHO grade 2 tumours at one end of the range and CNS WHO grade 4 tumours at the other" (see "Proliferation" section under "Astrocytoma, IDH-mutant").

The presence of necrosis and microvascular proliferation, along with the high mitotic activity and Ki-67 index, supports the diagnosis of a grade 3 tumor rather than a grade 2.

In conclusion, the combination of histological features, IDH mutation, 1p/19q co-deletion, and high-grade features (necrosis, microvascular proliferation, high mitotic activity) leads to the diagnosis of Oligodendroglioma, IDH-mutant and 1p/19q-codeleted, CNS WHO grade 3.

ChatGPT Response:

Based on the histopathological description provided, the diagnosis appears to be an **Oligodendroglioma, IDH-mutant and 1p/19q-codeleted, Grade III (Anaplastic Oligodendroglioma)**.

Here are the key features supporting this diagnosis:

1. **Monomorphic, hypercellular glial tumour**: This is typical of oligodendrogliomas.

2. **Monomorphic nuclei with fine, granular chromatin and perinuclear halos**: These are characteristic features of oligodendroglioma cells.
3. **Mitotic figures are commonly seen**: Increased mitotic activity is suggestive of a higher-grade tumour.
4. **Geographic necrosis and microvascular proliferations**: These are indicative of anaplastic changes, often seen in higher-grade tumours.
5. **Positive immunostaining for mutant IDH1**: This mutation is commonly seen in oligodendrogliomas.
6. **Retention of ATRX staining**: ATRX retention suggests the tumour is likely an oligodendroglioma rather than astrocytoma, where ATRX loss is more common.
7. **Ki-67 proliferation index of 20%**: This is relatively high, supporting a higher-grade tumour.
8. **1p/19q co-deletion on FISH**: This genetic alteration is diagnostic of oligodendroglioma.

Given these features, the tumour is best classified as an ****anaplastic oligodendroglioma (Grade III)**** due to the presence of necrosis, increased mitotic activity, and microvascular proliferation, along with the genetic and molecular markers typical of oligodendrogliomas.

ChatGPT RAG response:

Based on the histopathological description and immunohistochemical findings, the diagnosis is most likely ****Oligodendroglioma, IDH-mutant and 1p/19q-codeleted, CNS WHO Grade 3****.

Rationale:
1. **Histopathological Features**:
- **Monomorphic, hypercellular glial tumour**: Characteristic of oligodendroglioma.
- **Monomorphic nuclei with fine, granular chromatin and perinuclear halos**: Describes the classic "fried egg" appearance of oligodendroglioma cells.
- **Commonly seen mitotic figures**: Suggests a higher-grade tumour.
- **Geographic necrosis and microvascular proliferations**: Indicate a grade 3 tumour.

2. **Immunohistochemical Findings**:
- **Positive staining for mutant IDH1**: Confirms the presence of an IDH1 mutation, which is common in oligodendrogliomas.
- **Retention of ATRX staining**: Supports the diagnosis of oligodendroglioma, as ATRX loss is typically seen in astrocytomas.
- **Ki-67 proliferation index at 20%**: High proliferation index consistent with a grade 3 tumour.
- **1p/19q co-deletion on FISH**: Molecular hallmark of oligodendroglioma.

Supporting WHO Guidelines:
According to the WHO Classification of CNS Tumours (5th Edition):
- **Definition of Oligodendroglioma, IDH-mutant and 1p/19q-codeleted**: A diffusely infiltrating glioma with IDH1 or IDH2 mutation and 1p/19q codeletion. It can be graded as CNS WHO grade 2 or 3【3†source】.
- **Grading Criteria**: CNS WHO grade 3 oligodendrogliomas show several histological features of higher-grade malignancy such as high cellularity, marked cytological atypia, brisk mitotic activity, pathological microvascular proliferation, and necrosis【3†source】.

Given the presence of mitotic figures, geographic necrosis, frequent microvascular proliferations, and a high Ki-67 index, the tumour is best classified as ****Oligodendroglioma, IDH-mutant and 1p/19q-codeleted, CNS WHO Grade 3****.

O8: NH19 1552 - O3+FISH

Sections show fragments of grey and white matter with areas of diffuse infiltration by tumour. The tumour displays oligodendroglial features including rounded nuclei and perinuclear halos. Vascularity of the tumour is prominent, composed of a fine capillary network but no definite vascular proliferation is seen. The tumour cells show moderate pleomorphism, scattered infrequently with mitoses. There is no evidence of tumour necrosis.

Immunohistochemistry for IDH 1 mutation is positive and ATRX expression is retained in tumour cell nuclei. The Ki67 proliferation index is low, reaching approximately 5% in areas. FISH shows 1p19q co-deletion

What do you think the diagnosis would be?

GT: Oligodendroglioma, IDH-mutant and 1p/19q-codeleted, CNS WHO grade 3

Llama Response:

Based on the histopathological description, I would diagnose this tumour as an **Oligodendroglioma, WHO Grade II**.

The presence of oligodendroglial features, such as rounded nuclei and perinuclear halos, suggests an oligodendroglial tumour. The moderate pleomorphism and scattered mitoses indicate a low-grade tumour. The positive IDH1 mutation and retained ATRX expression support this diagnosis. The 1p19q co-deletion, as detected by FISH, is a characteristic genetic hallmark of oligodendrogliomas.

The low Ki67 proliferation index (approximately 5%) and lack of tumour necrosis also support a low-grade tumour diagnosis. Overall, the combination of morphology, immunohistochemistry, and molecular features points towards an Oligodendroglioma, WHO Grade II.

Llama RAG response:

Based on the provided histopathological description, I believe the diagnosis is **Oligodendroglioma, IDH-mutant, Grade 2**.

Here's how I reached this diagnosis using the attached document:

1. The presence of oligodendroglial features, such as rounded nuclei and perinuclear halos, suggests an oligodendroglial tumor (Section: CNS Tumour Nomenclature).
2. The positive IDH1 mutation immunohistochemistry and retained ATRX expression in tumor cell nuclei support an IDH-mutant oligodendroglioma diagnosis (Section: Gene and Protein Nomenclature).
3. The 1p19q co-deletion detected by FISH is a characteristic feature of oligodendrogliomas (Section: Novel diagnostic technologies).
4. The tumor's moderate pleomorphism, scattered mitoses, and low Ki67 proliferation index (approximately 5%) suggest a Grade 2 tumor (Section: Clinicopathological grading).

5. The absence of tumor necrosis and lack of vascular proliferation also support a Grade 2 diagnosis.

Please let me know if I'm correct or if I need to revisit my diagnosis!

Claude Response:

Based on the histopathological description and immunohistochemistry results provided, the diagnosis is most likely:

Oligodendroglioma, IDH-mutant and 1p/19q-codeleted, WHO grade 2

Reasoning:

1. Oligodendroglial features: The description mentions "rounded nuclei and perinuclear halos," which are classic features of oligodendrogliomas.
2. Diffuse infiltration: This is characteristic of diffuse gliomas.
3. Moderate pleomorphism and infrequent mitoses: These features suggest a lower-grade tumor.
4. Absence of necrosis and definite vascular proliferation: These features are typically associated with higher-grade tumors and are not present here.
5. IDH1 mutation positive: This is consistent with the IDH-mutant classification.
6. 1p/19q co-deletion: This is a defining feature of oligodendrogliomas.
7. ATRX expression retained: This is consistent with oligodendrogliomas (ATRX loss is more associated with astrocytomas).
8. Low Ki67 proliferation index (approximately 5%): This supports a lower-grade tumor.

The absence of high mitotic activity, vascular proliferation, and necrosis, combined with the low Ki67 index, supports a WHO grade 2 classification rather than a higher grade.

It's important to note that the final diagnosis should be made by a qualified pathologist, taking into account all clinical and radiological information in addition to the histopathological findings.

Claude RAG response:

Based on the histopathological description and immunohistochemistry results provided, and using only the information from the attached WHO diagnostic guidelines document, I believe the diagnosis is:

Oligodendroglioma, IDH-mutant and 1p/19q-codeleted, CNS WHO grade 2

Reasoning and references from the document:

1. Histological features:
   - The tumor displays oligodendroglial features, including rounded nuclei and perinuclear halos, which are characteristic of oligodendrogliomas. The document states: "Classic oligodendroglioma cells have uniformly round nuclei that are slightly larger than those of normal oligodendrocytes... In formalin-fixed, paraffin-embedded tissue, tumour cells often appear as rounded cells with well-defined cell membranes and clear cytoplasm around the central spherical nucleus. This creates

the typical honeycomb or fried-egg appearance" (Section: Oligodendroglioma, IDH-mutant and 1p/19q-codeleted, Histopathology).

2. Molecular features:
   - IDH1 mutation is positive, which is a key feature of this tumor type: "Oligodendrogliomas are molecularly defined by IDH1 or IDH2 mutations and 1p/19q codeletion" (Section: Diagnostic molecular pathology).
   - FISH shows 1p19q co-deletion, which is the second key molecular feature required for this diagnosis.
   - ATRX expression is retained, which is consistent with oligodendroglioma: "Most IDH-mutant and 1p/19q-codeleted oligodendrogliomas carry TERT promoter mutations; however, detection of a TERT promoter mutation in an IDH-mutant glioma is not sufficient for an oligodendroglioma diagnosis" (Section: Diagnostic molecular pathology).
3. Grading:
   - The tumor is classified as grade 2 based on the following features:
   - Moderate pleomorphism and infrequent mitoses
   - No microvascular proliferation or necrosis
   - Low Ki67 proliferation index (approximately 5%)
   - The document states: "Mitotic activity is low or absent in CNS WHO grade 2 oligodendrogliomas... Accordingly, the Ki-67 (MIB1) proliferation index is usually low (< 5%) in CNS WHO grade 2 oligodendrogliomas" (Section: Proliferation).
4. Essential diagnostic criteria:
   - The tumor meets the essential diagnostic criteria outlined in the document: "Oligodendroglioma, IDH-mutant and 1p/19q-codeleted must display features of a diffusely infiltrating glioma. In addition, this tumour must have IDH1 codon 132 or IDH2 codon 172 missense mutation and combined whole-arm deletions of 1p and 19q" (Section: Essential and desirable diagnostic criteria).

In conclusion, based solely on the information provided in the WHO diagnostic guidelines document, the histological features, molecular profile, and grading characteristics are consistent with a diagnosis of Oligodendroglioma, IDH-mutant and 1p/19q-codeleted, CNS WHO grade 2.

ChatGPT Response:

Based on the histopathological description provided, the diagnosis is likely **oligodendroglioma, WHO Grade II**. Here is the reasoning:

1. **Histopathological Features**:

- **Oligodendroglial features**: The presence of rounded nuclei and perinuclear halos is characteristic of oligodendrogliomas.
- **Vascularity**: A fine capillary network without definite vascular proliferation fits with the typical vascular pattern seen in oligodendrogliomas.
- **Moderate pleomorphism** and **infrequent mitoses**: These features suggest a lower-grade tumor.
- **No tumor necrosis**: The absence of necrosis further supports a lower grade, as necrosis is more often seen in higher-grade gliomas.

2. **Immunohistochemical and Molecular Findings**:

- **IDH1 mutation positive**: This is a common mutation in oligodendrogliomas.
- **ATRX expression retained**: Retained ATRX expression helps differentiate oligodendroglioma from astrocytomas, where ATRX loss is more common.
- **Low Ki67 proliferation index (~5%)**: This suggests a lower proliferative activity, consistent with a lower-grade tumor.
- **1p/19q co-deletion**: This genetic alteration is a defining feature of oligodendrogliomas and is used to distinguish them from other types of gliomas.

Taken together, these findings strongly suggest that the tumor is an **oligodendroglioma, WHO Grade II**.

ChatGPT RAG response:

Based on the provided histopathological description and immunohistochemical findings, the diagnosis is most consistent with **Oligodendroglioma, IDH-mutant and 1p/19q-codeleted, CNS WHO grade 2**.

Here's how the provided details fit this diagnosis according to the WHO guidelines:
1. **Histopathological Features**:

- **Oligodendroglial Features**: The tumour displays oligodendroglial features including rounded nuclei and perinuclear halos【3†source】.
- **Vascularity**: Prominent fine capillary network but no definite vascular proliferation is seen. This aligns with the characteristic vascular pattern seen in oligodendrogliomas【3†source】.
- **Moderate Pleomorphism and Low Mitotic Activity**: The tumour shows moderate pleomorphism with infrequent mitoses and a low Ki67 proliferation index (approximately 5%)【3†source】.

2. **Immunohistochemistry**: - **IDH1 Mutation Positive**: The positivity for IDH1 mutation indicates an IDH-mutant glioma【3†source】.

- **Retained ATRX Expression**: Retained ATRX expression supports the diagnosis of oligodendroglioma, as loss of ATRX would suggest an astrocytic tumour rather than an oligodendroglial one【3†source】.

3. **Molecular Findings**:

- **1p/19q Codeletion**: FISH showing 1p/19q codeletion is a definitive diagnostic criterion for oligodendroglioma IDH-mutant and 1p/19q-codeleted【3†source】.

4. **Absence of High-Grade Features**:

- **No Necrosis or Microvascular Proliferation**: The absence of tumour necrosis and microvascular proliferation is consistent with a lower grade tumour【3†source】.

References:

- **Essential Diagnostic Criteria**: For Oligodendroglioma, IDH-mutant and 1p/19q-codeleted, the tumour must display features of a diffusely infiltrating glioma, IDH1 or IDH2 mutation, and combined whole-arm deletions of 1p and 19q【3†source】.

- **Grading Criteria**: CNS WHO grade 2 oligodendrogliomas are characterized by lower mitotic activity, absence of microvascular proliferation, and absence of necrosis【3†source】.

Thus, the histopathological, immunohistochemical, and molecular findings collectively point towards **Oligodendroglioma, IDH-mutant and 1p/19q-codeleted, CNS WHO grade 2**.

O9: N18 2861 - O3+mol

Histology shows several large pieces of a pleomorphic cellular tumour composed of fascicles of cells with elongated and rounded nuclei and variably prominent cytoplasm. Ischaemic coagulative type tumour necrosis is present and there is brisk mitotic activity in the tumour. Lymphocytes are present in clusters in the stroma.
Immunostaining for mutant IDH1 (R132H) is positive. ATRX shows retained nuclear expression. Molecular analysis demonstrated IDH1 G395A mutant, LOH at 1p and 19q (co-deletion) and TERT C250T promoter mutation. H3f3 is wildtype (retained).

What do you think the diagnosis would be?

GT: Oligodendroglioma, IDH-mutant and 1p/19q-codeleted, CNS WHO grade 3

Llama Response:

What a fascinating case!

Based on the histopathological description, immunostaining results, and molecular analysis, I would diagnose this tumour as a:

****Grade IV Glioblastoma, IDH-mutant****

Here's my reasoning:

1. The histopathological description suggests a high-grade glioma, with features such as brisk mitotic activity, necrosis, and a pleomorphic cellular pattern.
2. The positive immunostaining for mutant IDH1 (R132H) is a hallmark of IDH-mutant gliomas.
3. The molecular analysis results are consistent with a glioblastoma:
    - IDH1 G395A mutation is a common mutation in glioblastomas.
    - LOH at 1p and 19q (co-deletion) is a characteristic genetic feature of oligodendrogliomas, but can also be seen in a subset of glioblastomas.
    - TERT C250T promoter mutation is a common mutation in glioblastomas.
    - The retained ATRX expression suggests that this is not an astrocytoma.
    - The wildtype H3F3A suggests that this is not a paediatric-type high-grade glioma.
4. The presence of lymphocytes in clusters in the stroma is consistent with the immune infiltrate often seen in glioblastomas.

Given the high-grade features, IDH mutation, and molecular profile, I would diagnose this tumour as a Grade IV Glioblastoma, IDH-mutant.

Llama RAG response:

Based on the provided histopathological description and molecular analysis, I believe the diagnosis is an **IDH-mutant astrocytoma**, specifically a **Grade 3 IDH-mutant astrocytoma**.

Here's my reasoning:

- The histopathological description mentions a pleomorphic cellular tumour with fascicles of cells, which is consistent with an astrocytoma.
- The presence of ischaemic coagulative type tumour necrosis and brisk mitotic activity suggests a higher-grade tumour.
- The positive immunostaining for mutant IDH1 (R132H) and retained ATRX nuclear expression are consistent with an IDH-mutant astrocytoma.
- The molecular analysis demonstrating IDH1 G395A mutant, LOH at 1p and 19q (co-deletion), and TERT C250T promoter mutation further supports this diagnosis.
- The wildtype H3F3A expression (retained) helps to rule out other possibilities.

According to the attached document, specifically the section on "CNS Tumour Grading" and "Combined histological and molecular grading", the presence of IDH mutation, LOH at 1p and 19q, and TERT promoter mutation suggests a higher-grade tumour. Given the brisk mitotic activity and ischaemic coagulative type tumour necrosis, I would assign a grade of 3 to this tumour.

References:

- Page 5, "CNS Tumour Grading" section: "IDH-mutant astrocytomas extend from grade 2 to grade 4..."
- Page 6, "Combined histological and molecular grading" section: "Examples in the fifth edition include CDKN2A and/or CDKN2B homozygous deletion in IDH-mutant astrocytomas, as well as TERT promoter mutation, EGFR amplification, and +7/−10 copy-number changes in IDH-wildtype glioblastoma."

Please let me know if I'm correct or not!

Claude Response:

Based on the histopathological description and molecular findings provided, the most likely diagnosis is:

Anaplastic (Grade III) Oligodendroglioma, IDH-mutant and 1p/19q co-deleted

Reasons for this diagnosis:

1. Histology:
   - Pleomorphic cellular tumor with fascicles of cells
   - Presence of necrosis
   - Brisk mitotic activity
   - Lymphocytic infiltration in the stroma
2. Immunohistochemistry:
   - Positive for mutant IDH1 (R132H)
   - Retained ATRX nuclear expression (which is typical for oligodendrogliomas, as opposed to astrocytomas which often lose ATRX expression)
3. Molecular findings:

- IDH1 G395A mutation (consistent with the immunostaining result)
- 1p/19q co-deletion (characteristic of oligodendrogliomas)
- TERT promoter mutation (common in oligodendrogliomas)
- Wild-type H3F3A (excludes histone H3-mutant gliomas)

The presence of necrosis and brisk mitotic activity suggests a higher-grade tumor, supporting the diagnosis of an anaplastic (Grade III) oligodendroglioma rather than a lower-grade (Grade II) oligodendroglioma.

The combination of IDH mutation and 1p/19q co-deletion is diagnostic for oligodendroglioma according to the current WHO classification of CNS tumors. The additional findings, including the TERT promoter mutation and retained ATRX expression, further support this diagnosis.

Claude RAG response:

Based on the histological description and molecular findings provided, I believe the diagnosis would be:

Oligodendroglioma, IDH-mutant and 1p/19q-codeleted, CNS WHO grade 3

Here's my reasoning, referencing the attached WHO diagnostic guidelines:

1. IDH mutation: The tumor is positive for IDH1 R132H immunostaining and molecular analysis confirms IDH1 G395A mutation. This is consistent with the definition of IDH-mutant gliomas (see "Essential and desirable diagnostic criteria" for Oligodendroglioma).
2. 1p/19q codeletion: Molecular analysis shows LOH (loss of heterozygosity) at 1p and 19q, indicating codeletion. This is a defining feature of oligodendroglioma (see "Definition" and "Essential and desirable diagnostic criteria" for Oligodendroglioma).
3. ATRX status: Nuclear expression of ATRX is retained, which is typical for oligodendrogliomas and helps differentiate from astrocytomas (see "Immunophenotype" section for Oligodendroglioma).
4. TERT promoter mutation: The C250T TERT promoter mutation is present, which is common in oligodendrogliomas (see "Diagnostic molecular pathology" section for Oligodendroglioma).
5. Histological features: The description mentions pleomorphism, fascicles of cells with elongated and rounded nuclei, which can be seen in oligodendrogliomas. The presence of lymphocytes is also noted in the WHO description (see "Histopathology" section for Oligodendroglioma).
6. Grade 3 features: The presence of "ischaemic coagulative type tumour necrosis" and "brisk mitotic activity" are indicative of a higher grade (grade 3) oligodendroglioma (see "Grading" section for Oligodendroglioma).

The combination of IDH mutation, 1p/19q codeletion, and the histological features described, along with the molecular findings, strongly support the diagnosis of an Oligodendroglioma, IDH-mutant and 1p/19q-codeleted, CNS WHO grade 3.

ChatGPT Response:

Based on the histopathological description and molecular findings provided, the diagnosis is likely to be **anaplastic oligodendroglioma, IDH-mutant and 1p/19q co-deleted**. Here's a breakdown of how this conclusion is reached:

1. **Histological Features**:
   - **Pleomorphic cellular tumour with fascicles of cells**: This is consistent with a high-grade glioma.
   - **Ischaemic coagulative necrosis** and **brisk mitotic activity**: These features suggest a high-grade tumour, indicating anaplastic characteristics.
   - **Lymphocytes in clusters in the stroma**: This can be seen in various gliomas but does not narrow it down specifically.
2. **Immunohistochemistry**:
   - **Positive for mutant IDH1 (R132H)**: This mutation is commonly seen in lower-grade gliomas and glioblastomas.
   - **ATRX retained**: Retained ATRX expression helps differentiate oligodendrogliomas (which retain ATRX) from astrocytomas (which often lose ATRX expression).
3. **Molecular Findings**:
   - **IDH1 G395A mutant**: Confirms the presence of an IDH1 mutation.
   - **LOH at 1p and 19q (co-deletion)**: This is characteristic of oligodendrogliomas.
   - **TERT C250T promoter mutation**: This mutation is often found in gliomas, including oligodendrogliomas and glioblastomas.
   - **H3f3 wildtype (retained)**: This indicates that the tumour does not have the H3 K27M mutation, which is often seen in diffuse midline gliomas.

Combining these features, the presence of IDH mutation and 1p/19q co-deletion are key diagnostic criteria for oligodendrogliomas. The high-grade features (necrosis, high mitotic activity) and molecular characteristics (TERT mutation) suggest it is an **anaplastic (WHO grade III)** form.

Therefore, the most likely diagnosis is **anaplastic oligodendroglioma, IDH-mutant and 1p/19q co-deleted**.

ChatGPT RAG response:

Based on the histopathological and molecular findings you have provided, the diagnosis appears to be **Oligodendroglioma, IDH-mutant and 1p/19q-codeleted, CNS WHO grade 3**.

Here is the rationale based on the WHO diagnostic guidelines from the provided document:
1. **Histological Description**:
   - **Pleomorphic cellular tumour**: Characteristic of higher-grade gliomas.
   - **Fascicles of cells with elongated and rounded nuclei and variably prominent cytoplasm**: These features can be seen in oligodendrogliomas.
   - **Ischaemic coagulative type tumour necrosis and brisk mitotic activity**: Indicative of a higher-grade tumour, consistent with CNS WHO grade 3.
   - **Lymphocytes present in clusters in the stroma**: This can be seen in various types of gliomas but does not specifically alter the diagnosis.
2. **Immunostaining and Molecular Analysis**:
   - **Positive for mutant IDH1 (R132H)**: This confirms the tumour is IDH-mutant.
   - **Retained ATRX expression**: This supports the diagnosis of oligodendroglioma over astrocytoma, as loss of ATRX is more common in IDH-mutant astrocytomas.

- **LOH at 1p and 19q (co-deletion)**: This molecular feature is definitive for oligodendroglioma.
- **TERT C250T promoter mutation**: This is frequently found in oligodendrogliomas.
- **H3f3 wildtype**: Excludes certain other types of high-grade gliomas.

Conclusion:
The presence of IDH1 mutation, 1p/19q co-deletion, and retained ATRX expression strongly supports the diagnosis of oligodendroglioma. The histological features of necrosis and brisk mitotic activity indicate a higher grade, leading to the diagnosis of **Oligodendroglioma, IDH-mutant and 1p/19q-codeleted, CNS WHO grade 3**.

References

- WHO Classification of Tumours. Central Nervous System Tumours, 5th Edition. See sections on Oligodendroglioma IDH-mutant and 1p/19q-codeleted, histopathology, immunophenotype, and grading.

O10: NH14 1648 - O3+mol

This is a stereotaxic biopsy specimen containing several pieces of hypercellular glial tumour. The tumour nuclei are monomorphic but perinuclear clearing is rare. Fresh haemorrhages are present, as are areas with incipient microvascular proliferations. Immunostaining is strongly positive for mutant IDH1 (R132H) in tumour cell nuclei. Similarly, ATRX immunostaining is strong in tumour cell nuclei. Molecular testing shows loss of heterozygosity for chromosomes 1p and 19q.

What do you think the diagnosis would be?

GT: Oligodendroglioma, IDH-mutant and 1p/19q-codeleted, CNS WHO grade 3

Llama Response:

A great challenge!

Based on the histopathological description, I think the diagnosis is:

****Oligodendroglioma, IDH-mutant, 1p/19q-codeleted, Grade II****

Here's why:
- The presence of hypercellular glial tumor with monomorphic nuclei suggests a glial neoplasm.
- The presence of fresh hemorrhages and incipient microvascular proliferations is consistent with an oligodendroglioma.
- The strong positivity for mutant IDH1 (R132H) and ATRX in tumor cell nuclei supports the diagnosis of an IDH-mutant glioma, which is characteristic of oligodendrogliomas.
- The loss of heterozygosity for chromosomes 1p and 19q is a hallmark of oligodendrogliomas, particularly those with IDH mutations.
- The lack of significant nuclear atypia and perinuclear clearing suggests a lower-grade tumor, which is consistent with a Grade II oligodendroglioma.

Please let me know if I'm correct!

Llama RAG response:

Based on the provided histopathological description, I believe the diagnosis would be **Oligodendroglioma, IDH-mutant and 1p/19q-codeleted, Grade 2**.

Here's my reasoning:
- The presence of perinuclear clearing is not a dominant feature, which suggests that the tumour is not an Astrocytoma (Page 5, CNS Tumour Nomenclature).
- The strong positivity for mutant IDH1 (R132H) in tumour cell nuclei suggests that the tumour is an IDH-mutant glioma (Page 5, CNS Tumour Nomenclature).
- The loss of heterozygosity for chromosomes 1p and 19q is consistent with an Oligodendroglioma (Page 5, CNS Tumour Nomenclature).

- The presence of fresh haemorrhages and areas with incipient microvascular proliferations suggests that the tumour is a Grade 2 glioma (Page 10, Grading within Types).
- The strong ATRX immunostaining in tumour cell nuclei is consistent with an Oligodendroglioma (Page 5, CNS Tumour Nomenclature).

Therefore, based on the attached document, I believe the diagnosis is Oligodendroglioma, IDH-mutant and 1p/19q-codeleted, Grade 2.

Claude Response:

Based on the histopathological description and molecular findings provided, the most likely diagnosis is:

Oligodendroglioma, WHO grade 2

Reasoning:
1. The description mentions a hypercellular glial tumor, which is consistent with a glioma.
2. The presence of monomorphic nuclei with rare perinuclear clearing is typical of oligodendrogliomas.
3. The tumor is IDH1 (R132H) mutant, which is a characteristic feature of lower-grade gliomas, including oligodendrogliomas.
4. ATRX immunostaining is strong in tumor cell nuclei, which is consistent with retained ATRX expression. This is typical for oligodendrogliomas (as opposed to astrocytomas, which often show ATRX loss).
5. The molecular testing shows loss of heterozygosity for chromosomes 1p and 19q (1p/19q codeletion), which is the defining molecular feature of oligodendrogliomas.
6. The presence of fresh hemorrhages and areas with incipient microvascular proliferations suggests some progression, but these features alone are not sufficient to classify it as a grade 3 tumor.

Given these features, particularly the 1p/19q codeletion and IDH1 mutation, the most appropriate diagnosis is a WHO grade 2 oligodendroglioma. If there were more prominent microvascular proliferation or significant mitotic activity (which is not mentioned in the description), it could potentially be classified as a grade 3 anaplastic oligodendroglioma. However, based on the information provided, a grade 2 classification seems most appropriate.

Claude RAG response:

Based on the histopathological description and molecular findings provided, and using only the information from the attached WHO diagnostic guidelines document, I believe the diagnosis for this tumor is:

Oligodendroglioma, IDH-mutant and 1p/19q-codeleted, CNS WHO grade 3

Here's my reasoning, supported by references from the document:
1. IDH mutation: The tumor is strongly positive for mutant IDH1 (R132H) in tumor cell nuclei. This is a key diagnostic feature of IDH-mutant oligodendrogliomas, as stated in the document: "Oligodendrogliomas are molecularly defined by IDH1 or IDH2 mutations and 1p/19q codeletion."

2. 1p/19q codeletion: Molecular testing shows loss of heterozygosity for chromosomes 1p and 19q, which is another defining feature of oligodendroglioma. The document states: "Diagnosis of oligodendrogliomas requires demonstration of IDH mutation by IDH1 p.R132H immunohistochemistry and/or sequencing of the IDH1 or IDH2 gene, as well as demonstration of 1p/19q codeletion."
3. ATRX retention: The tumor shows strong ATRX immunostaining in tumor cell nuclei. This is consistent with oligodendroglioma, as the document notes: "IDH-mutant and 1p/19q-codeleted oligodendrogliomas retain nuclear expression of ATRX."
4. Grade 3 features: The presence of incipient microvascular proliferations suggests a higher grade. The document states for CNS WHO grade 3 oligodendrogliomas: "CNS WHO grade 3 astrocytomas are characterised by a diffusely infiltrative astrocytic glioma with an IDH1 or IDH2 mutation that exhibits focal or dispersed anaplasia and displays significant mitotic activity. Microvascular proliferation, necrosis, and homozygous deletions of CDKN2A and/or CDKN2B are absent." While the description doesn't mention significant mitotic activity, the presence of incipient microvascular proliferations suggests a more aggressive tumor.
5. Histological features: While the description mentions that "perinuclear clearing is rare," which is atypical for classic oligodendroglioma, the document emphasizes that molecular features take precedence over histological appearance: "Thus, irrespective of oligodendroglial, oligoastrocytic, astrocytic, or ambiguous features on histology, detection of combined IDH mutation and 1p/19q codeletion indicates an IDH-mutant and 1p/19q-codeleted oligodendroglioma."

In conclusion, the combination of IDH mutation, 1p/19q codeletion, and retained ATRX expression, along with the presence of incipient microvascular proliferations, supports the diagnosis of Oligodendroglioma, IDH-mutant and 1p/19q-codeleted, CNS WHO grade 3.

ChatGPT Response:

The histopathological and molecular findings described are consistent with an ****oligodendroglioma, IDH-mutant and 1p/19q codeleted****.

Key points supporting this diagnosis:
1. **Hypercellular glial tumour with monomorphic nuclei**: This is a typical histological feature of oligodendrogliomas.
2. **Perinuclear clearing is rare**: This indicates that the "fried egg" appearance, which is characteristic but not always present in oligodendrogliomas, is not prominent here.
3. **Fresh haemorrhages and incipient microvascular proliferations**: These features can be seen in higher-grade gliomas but can also be present in oligodendrogliomas, especially in anaplastic (higher-grade) forms.
4. **Immunostaining positive for mutant IDH1 (R132H)**: This mutation is common in oligodendrogliomas and astrocytomas.
5. **Strong ATRX immunostaining**: ATRX retention is consistent with oligodendrogliomas, as ATRX loss is more commonly associated with astrocytomas.
6. **Loss of heterozygosity for chromosomes 1p and 19q**: This is a hallmark of oligodendrogliomas and is essential for the diagnosis.

Based on these findings, the diagnosis is likely **oligodendroglioma, IDH-mutant and 1p/19q-codeleted**. The presence of microvascular proliferation suggests that it may be a higher grade, possibly an **anaplastic oligodendroglioma (WHO Grade III)**, but confirmation of grade would typically require additional information such as mitotic activity and necrosis.

ChatGPT RAG response:

Based on the provided histopathological description and the WHO guidelines, the tumor can be diagnosed as an **oligodendroglioma, IDH-mutant and 1p/19q-codeleted**, with the following reasoning:

1. **Hypercellular glial tumor**: The presence of a hypercellular glial tumor is consistent with a glioma.
2. **Monomorphic tumor nuclei**: This feature fits with oligodendroglioma, which typically has monomorphic nuclei.
3. **Perinuclear clearing is rare**: While oligodendrogliomas can have perinuclear clearing (the "fried-egg" appearance), it is not always prominent in all cases.
4. **Fresh hemorrhages and incipient microvascular proliferations**: These findings suggest the presence of higher-grade features but are not definitive for grade determination on their own.
5. **Strong positive immunostaining for mutant IDH1 (R132H)**: This indicates that the tumor is an IDH-mutant glioma.
6. **Strong ATRX immunostaining in tumor cell nuclei**: Retained ATRX expression is characteristic of oligodendrogliomas, as ATRX mutations are typically seen in astrocytomas.
7. **Loss of heterozygosity for chromosomes 1p and 19q**: This is a defining molecular feature of oligodendroglioma.

Considering these features together, the most appropriate diagnosis is **oligodendroglioma, IDH-mutant and 1p/19q-codeleted**. The presence of IDH mutation and 1p/19q codeletion molecularly confirms this diagnosis.

For the grade, the presence of hypercellularity, microvascular proliferation, and hemorrhage suggests a higher grade. Given these findings, this tumor likely corresponds to a **CNS WHO grade 3 oligodendroglioma**, as higher mitotic activity and microvascular proliferation are indicative of a higher-grade tumor.

Final Diagnosis:
Oligodendroglioma, IDH-mutant and 1p/19q-codeleted, CNS WHO grade 3

References:
- "Oligodendroglioma, IDH-mutant and 1p/19q-codeleted" in WHO Classification of Tumours, Central Nervous System Tumours, 5th Edition【3†source】.
- Diagnostic criteria for oligodendroglioma, including IDH mutation and 1p/19q codeletion【3†source】.
